# Supplementary material for: linc-ADAIN, a human adipose lincRNA, regulates adipogenesis by modulating KLF5 and IL-8 mRNA stability
Source: Cell Rep. Author manuscript; Available in PMC 2024 Aug 20. (PMC11334222; doi:10.1016/j.celrep.2024.114240)
Supplement: Supplementary Material [file NIHMS2009043-supplement-Supplementary_Material.zip › 1-s2.0-S2211124724005680-mmc1.pdf]

Cell Reports, Volume *43*

Supplemental information

linc-ADAIN, a human adipose lincRNA, regulates adipogenesis by modulating KLF5 and IL-8 mRNA stability

Marcella E. O'Reilly, Sebastian Ho, Johana Coronel, Lucie Zhu, Wen Liu, Chenyi Xue, Eunyoung Kim, Esther Cynn, Caio V. Matias, Rajesh Kumar Soni, Chen Wang, Iuliana Ionita-Laza, Robert C. Bauer, Leila Ross, Yiying Zhang, Silvia Corvera, Susan K. Fried, and Muredach P. Reilly

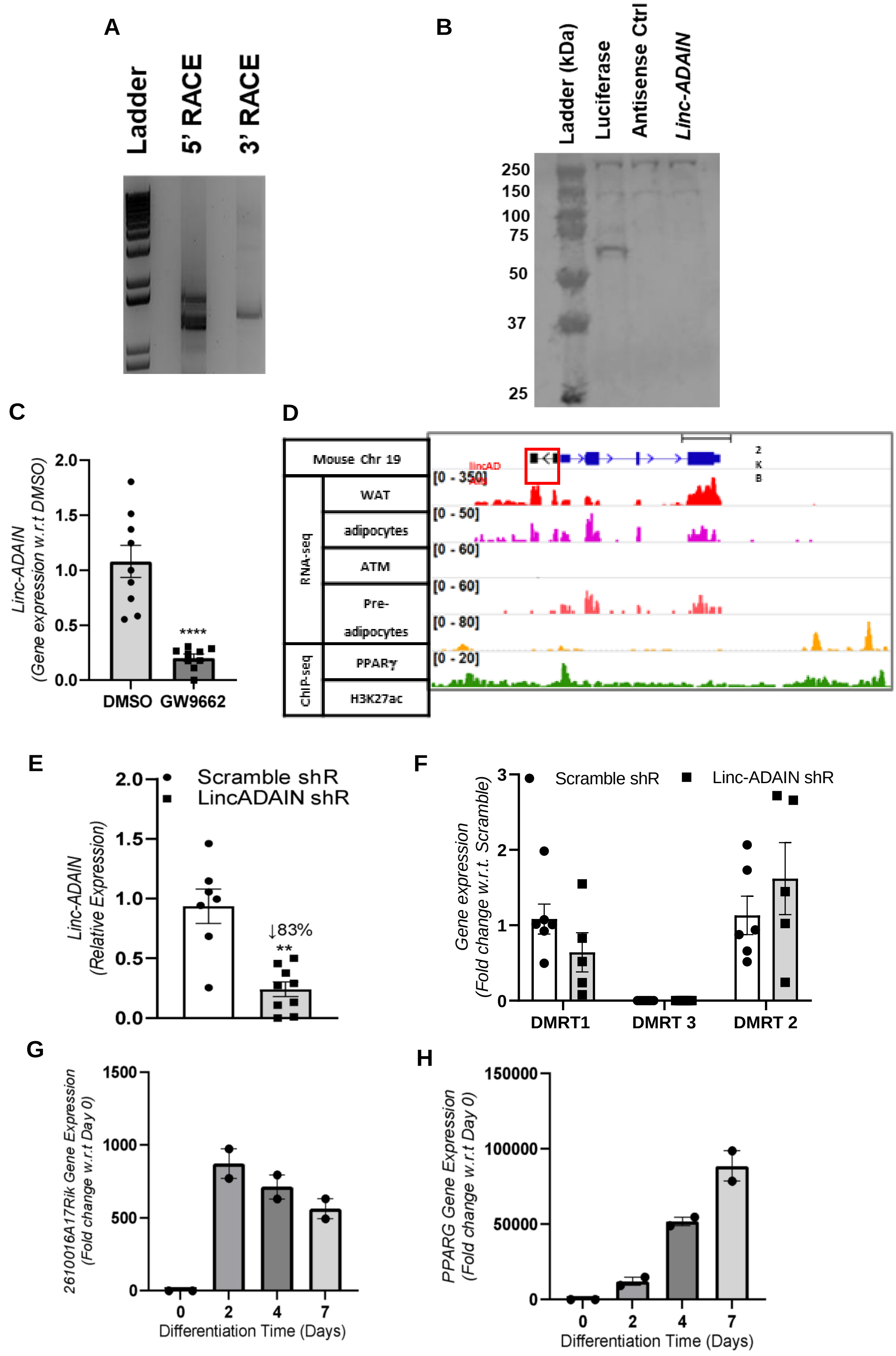

A
Ladder
5' RACE
3' RACE
B
Ladder (kDa)
Luciferase
Antisense Ctrl
Linc-ADAIN
250
150
100
75
50
37
25
C
Linc-ADAIN (Gene expression w.r.t DMSO)
2.0
1.5
1.0
0.5
0.0
****
DMSO
GW9662
D
Mouse Chr 19
lincADAIN
RNA-seq
WAT
adipocytes
ATM
Pre-adipocytes
ChIP-seq
PPARγ
H3K27ac
[0 - 350]
[0 - 50]
[0 - 60]
[0 - 60]
[0 - 80]
[0 - 20]
2 KB
E
Scramble shR
LincADAIN shR
Linc-ADAIN (Relative Expression)
2.0
1.5
1.0
0.5
0.0
↓83%
**
F
Scramble shR
Linc-ADAIN shR
Gene expression (Fold change w.r.t. Scramble)
3
2
1
0
DMRT1
DMRT 3
DMRT 2
G
2610016A17Rik Gene Expression (Fold change w.r.t Day 0)
1500
1000
500
0
0
2
4
7
Differentiation Time (Days)
H
PPARG Gene Expression (Fold change w.r.t Day 0)
150000
100000
50000
0
0
2
4
7
Differentiation Time (Days)

Supplement Figure 1. *Linc-ADAIN* does not code for protein and the characteristics of *linc-ADAIN's* syntenic locus in mouse. The 3' RACE products of *linc-ADAIN* in human ASC-adipocytes (A), An *in vitro* transcription/translation assay using a pcDNA3.1 expressing *linc-ADAIN* or antisense *linc-ADAIN* and a luciferase vector as the positive control (B). Suppression of linc-ADAIN by PPARγ antagonist (GW9662 10μM (C). Regulatory features in the mouse syntenic region of *linc-ADAIN*, RNA-seq coverage (mouse adipose and adipocytes), transcription factor binding and active histone modification markers (D). qPCR of linc-ADAIN (E) and nearby protein coding genes, DMRT1, DMRT3, DMRT2 (F) upon linc-ADAIN KD via shRNA vs Scramble control in mature ASC hTERT adipocytes (N=3) **p<0.01 w.r.t Scramble by Two Mann Whitney U Test. 3T3-L1 adipocytes were differentiated in vitro and RNA extracted at the times indicated. Gene expression for mouse 2610016A17Rik. (G) and PPARG (H) was measured by qPCR at the times indicated. (N=2) Data presented as ± SEM.

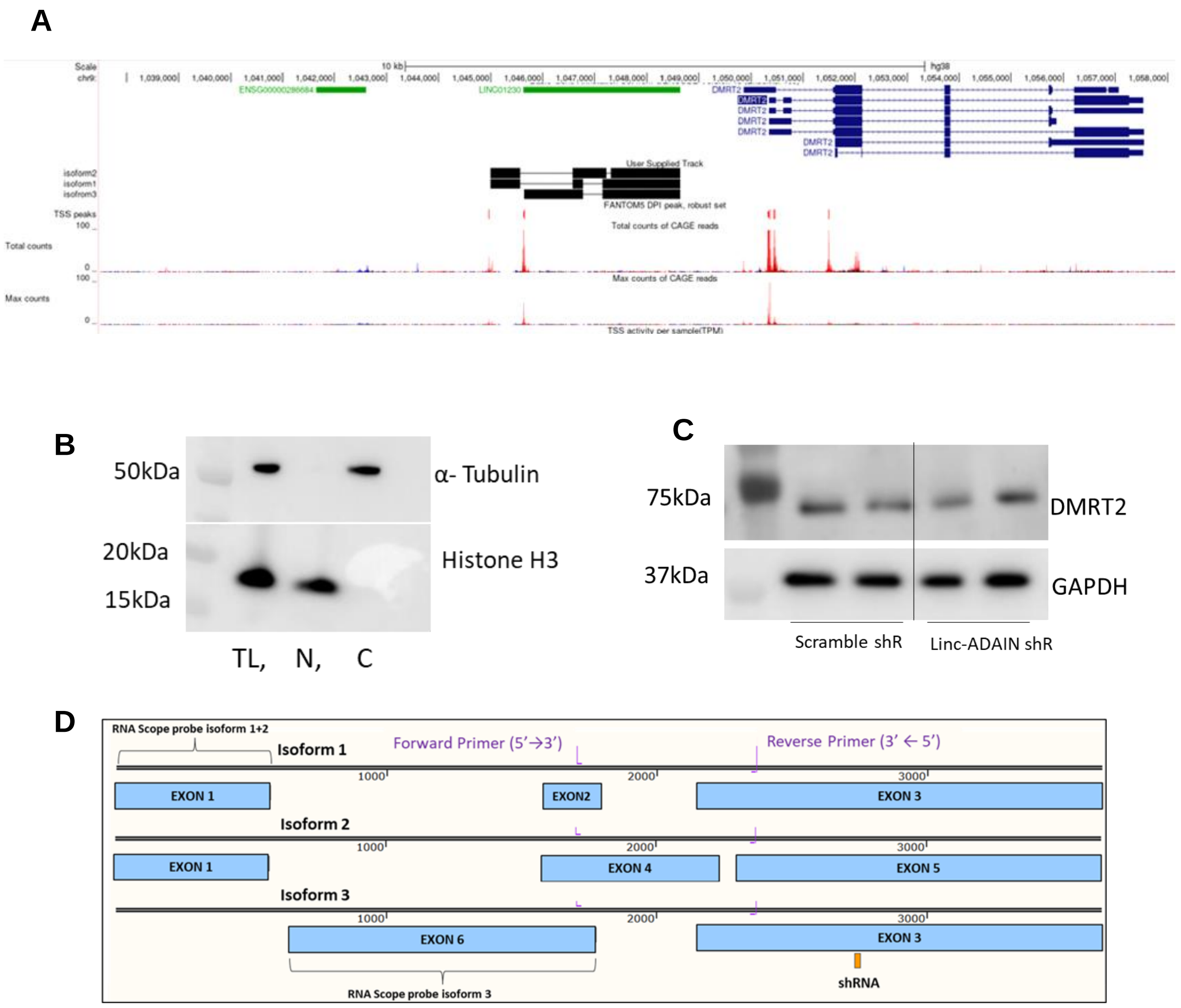

Supplement Figure 2. *Linc-ADAIN's* genome locus and map of isoforms. Genome Browser view of *linc-ADAIN* locus showing 5'TSS and total CAGE reads (A). WB showing fractionation of adipocytes into nuclear [N] and cytoplasm [B] fractions, probing for α-tubulin (cytoplasm) and histone H3 (nuclear) (B). WB of DMRT2 expression upon *linc-ADAIN* KD via shRNA in adipocytes (C). Map of *linc-ADAIN Isoforms*, exons, qPCR primer location, RNA scope probe location and shRNA target sequence (D).

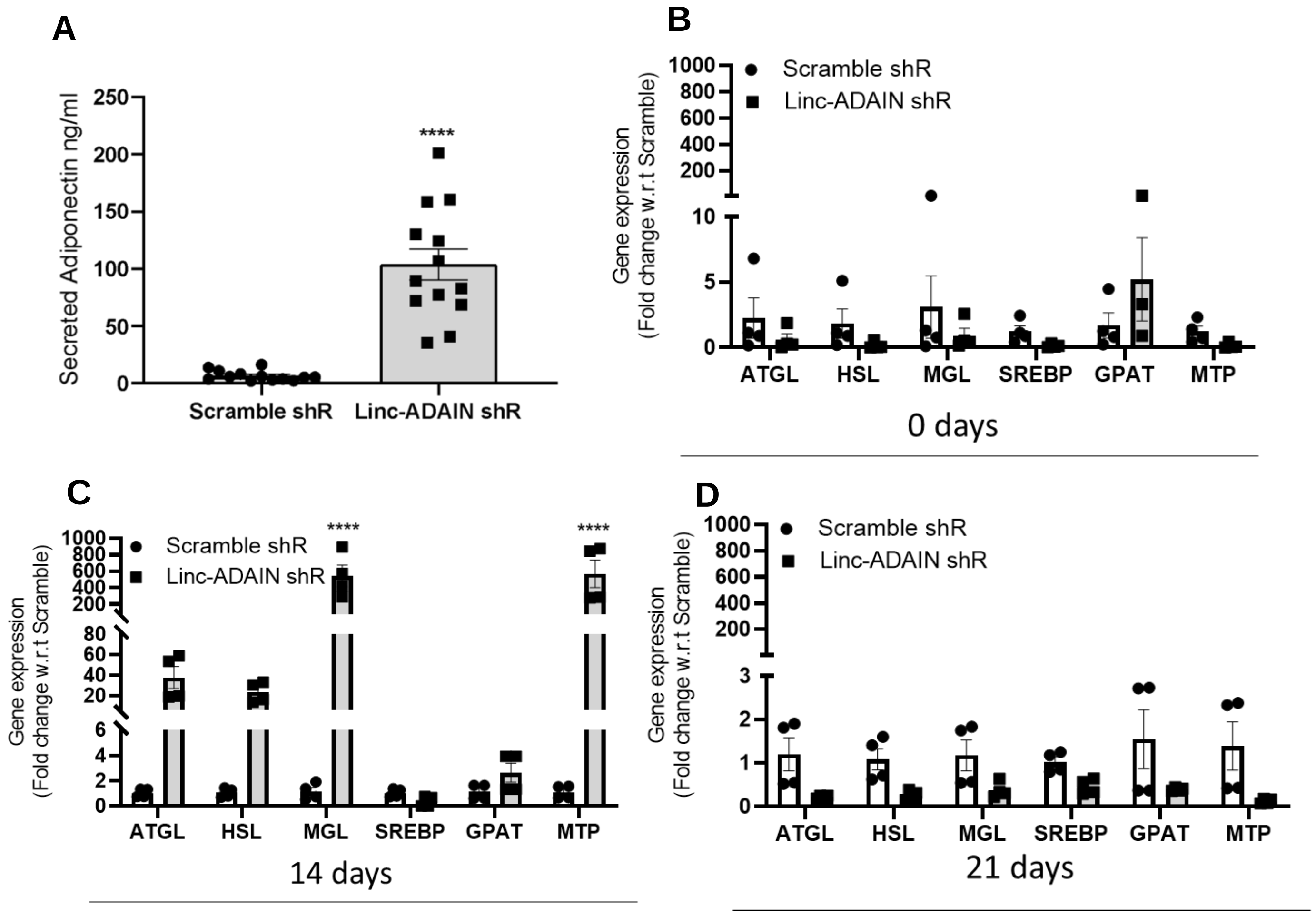

Human cytokine array

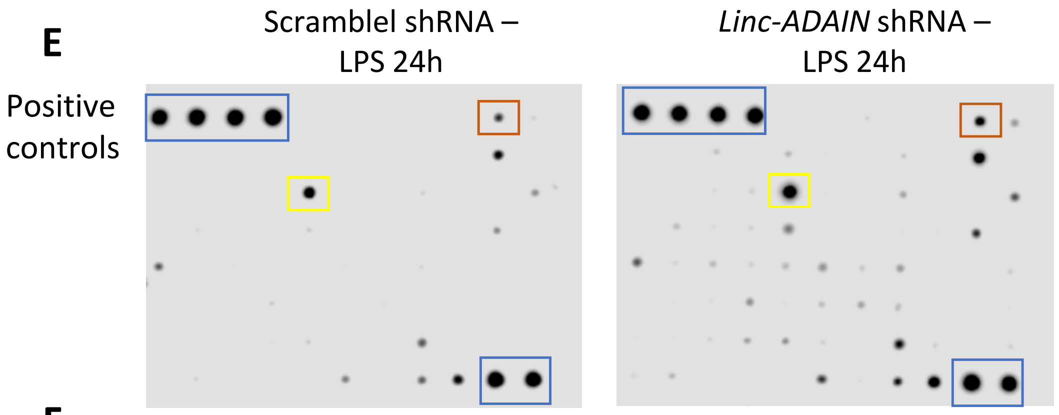

F

	A	B	C	D	E	F	G	H	I	J	K
1	POS	POS	POS	POS	NEG	NEG	ENA-78 (CXCL5)	G-CSF	GM-CSF	GRO a/b/g	GRO alpha (CXCL1)
2	I-309 (CCL1)	IL-1 alpha (IL-1 F1)	IL-1 beta (IL-1 F2)	IL-2	IL-3	IL-4	IL-5	IL-6	IL-7	IL-8 (CXCL8)	IL-10
3	IL-12 p40/p70	IL-13	IL-15	IFN-gamma	MCP-1 (CCL2)	MCP-2 (CCL8)	MCP-3 (CCL7)	M-CSF	MDC (CCL22)	MIG (CXCL9)	MIP-1 beta (CCL4)
4	MIP-1 delta (CCL15)	RANTES (CCL5)	SCF	SDF-1 alpha	TARC (CCL17)	TGF beta 1	TNF alpha	TNF beta (TNFSF1B)	EGF	IGF-1	Angiogenin
5	OSM	TPO	VEGF-A	PDGF-BB	Leptin	BDNF	BLC (CXCL13)	Ck beta 8-1 (CCL23)	Eotaxin-1 (CCL11)	Eotaxin-2 (CCL24)	Eotaxin-3 (CCL26)
6	FGF-4	FGF-6	FGF-7 (KGF)	FGF-9	FLT-3 Ligand	Fractalkine (CX3CL1)	GCP-2 (CXCL6)	GDNF	HGF	IGFBP-1	IGFBP-2
7	IGFBP-3	IGFBP-4	IL-16	IP-10 (CXCL10)	LIF	LIGHT (TNFSF14)	MCP-4 (CCL13)	MIF	MIP-3 alpha (CCL20)	NAP-2 (CXCL7)	NT-3
8	NT-4	OPN (SPP1)	OPG (TNFRSF11B)	PARC	PLGF	TGF beta 2	TGF beta 3	TIMP-1	TIMP-2	POS	POS

Supplement Figure 3. Effect of *linc-ADAIN* knock-down on adipogenic genes and secretion of cytokines from adipocytes. ASC hTERT adipocytes with scramble or *linc-ADAIN* shRNA expression with Adiponectin ELISA (A), ****$p<0.0001$ w.r.t to scramble shRNA by Mann Whitney U test. ASC hTERTs expressing Scramble or *linc-ADAIN* shRNA were grown in culture and RNA samples extracted at Day 0 (B), before differentiation or cells were differentiated to adipocytes and RNA extracted at Day 14 (C) and Day 21 (D) and genes ATGL, HSL, MGL, SREBP, GPAT and MTP measured by qPCR. ***$p<0.0001$ w.r.t Scramble shR (N=4). ASC hTERTs expressing Scramble or linc-ADAIN shRNA were differentiated to adipocytes and at day 14, 100ng of LPS was added to each group for 24hrs. Media was collected and applied to a Human Cytokine Antibody Array C5 (RayBio C Series) to detect changes in adipocyte cytokine secretion. Yellow box highlighs MCP-1, the orange box highlights IL-8 (E) Map of Cytokine Array (F). Data presented as ± SEM.

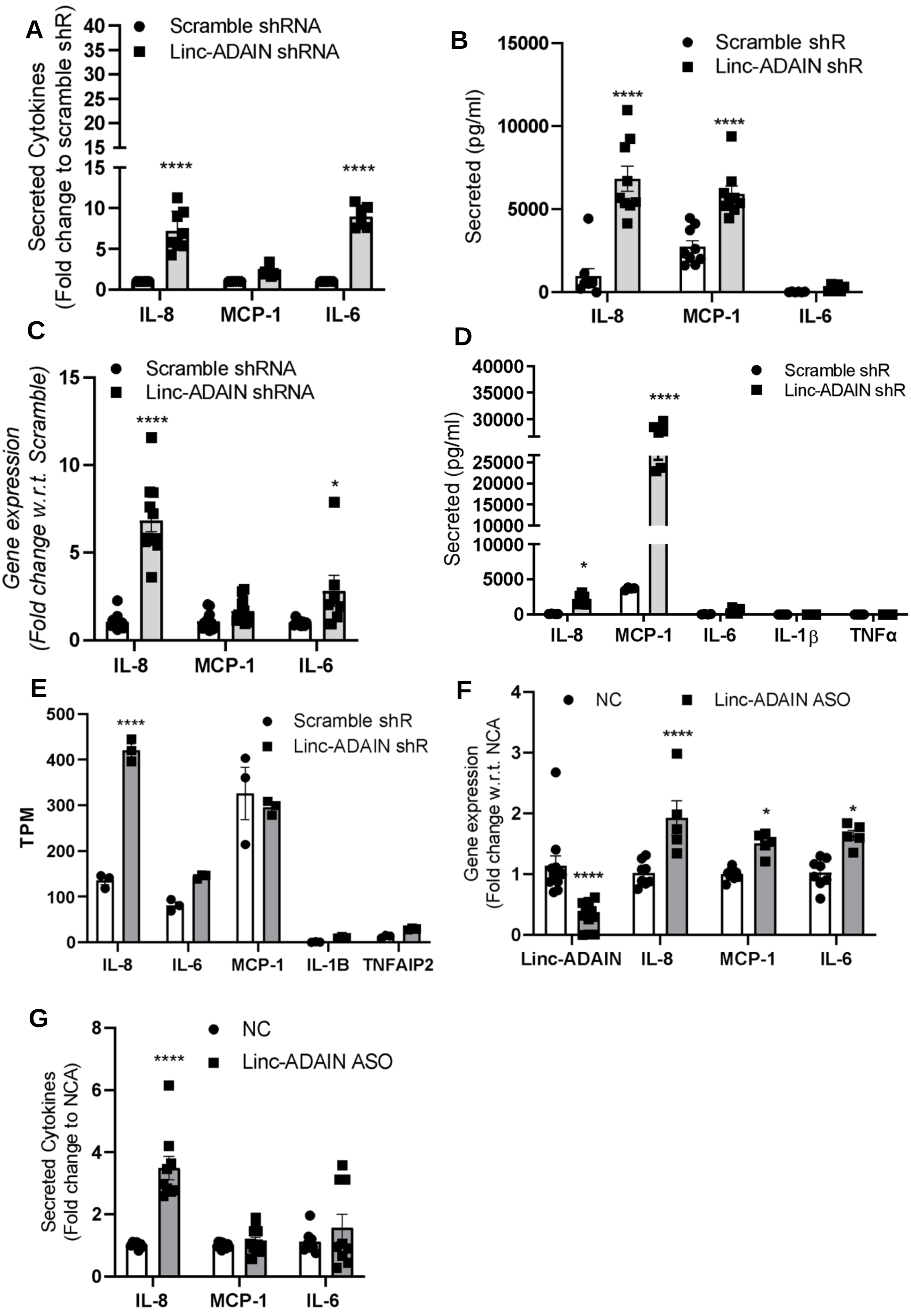
A
Secreted Cytokines
(Fold change to scramble shR)
Scramble shRNA
Linc-ADAIN shRNA
40
35
30
25
20
15
15
10
5
0
****
****
IL-8
MCP-1
IL-6
B
Secreted (pg/ml)
Scramble shR
Linc-ADAIN shR
15000
10000
5000
0
****
****
IL-8
MCP-1
IL-6
C
Gene expression
(Fold change w.r.t. Scramble)
Scramble shRNA
Linc-ADAIN shRNA
15
10
5
0
****
*
IL-8
MCP-1
IL-6
D
Secreted (pg/ml)
Scramble shR
Linc-ADAIN shR
40000
35000
30000
25000
20000
15000
10000
10000
5000
0
*
****
IL-8
MCP-1
IL-6
IL-1β
TNFα
E
TPM
Scramble shR
Linc-ADAIN shR
500
400
300
200
100
0
****
IL-8
IL-6
MCP-1
IL-1B
TNFAIP2
F
Gene expression
(Fold change w.r.t. NCA)
NC
Linc-ADAIN ASO
4
3
2
1
0
****
****
*
*
Linc-ADAIN
IL-8
MCP-1
IL-6
G
Secreted Cytokines
(Fold change to NCA)
NC
Linc-ADAIN ASO
8
6
4
2
0
****
IL-8
MCP-1
IL-6

Supplement Figure 4. Effect of *linc-ADAIN* knock-down in adipocytes on cytokine expression by ELISA, qPCR and RNA-sequencing. *Linc-ADAIN* knockdown increases cytokine secretion. Fold change of MCP-1, IL-8 and IL-6 via secretion ELISA (A) and expression of mRNA levels by qPCR (C) (N=3 in triplicate. Protein secretion of MCP-1, IL-8 and IL-6 via ELISA as pg/ml (D). **$p<0.01$, ****$p<0.0001$ w.r.t. scramble shR by Two-Way ANOVA (B) Luminex adipocyte panel of adipocyte media as pg/ml (N=3 in duplicate) (D). Transcripts per million (TPM) of cytokine mRNAs via RNA-seq upon *linc-ADAIN* KD (N=1 in triplicate) (E). Expression of *linc-ADAIN*, *IL-8*, *MCP-1* and *IL-6* by qPCR (F) and corresponding secretion via ELISA (G) upon knockdown of *linc-ADAIN* using an Antisense Oligonucleotide (ASO) throughout differentiation in primary human ASC adipocytes (N=3 in duplicate) ****$p<0.0001$, *$p<0.05$ w.r.t. NC (negative control) by Two-Way ANOVA. Data presented as ± SEM.

A

Molecular and Cellular Functions		
Name	p-value range	# Molecules
Cellular Movement	1.71E-07 - 7.96E-53	608
Cell Death and Survival	1.25E-07 - 2.34E-30	698
Cellular Development	1.02E-07 - 2.02E-22	641
Cellular Growth and Proliferation	5.82E-08 - 2.02E-22	618
Cell-To-Cell Signaling and Interaction	1.51E-07 - 2.44E-19	346

B

Term	Count	P-Value	Fold Enrichment	Bonferroni
GO:0007155~cell adhesion	145	2.63E-21	2.24	1.74E-17
GO:0001525~angiogenesis	72	8.31E-13	2.44	5.50E-09
GO:0006954~inflammatory response	94	2.40E-10	1.95	1.59E-06
GO:0030335~positive regulation of cell migration	66	3.06E-09	2.14	2.02E-05
GO:0007165~signal transduction	216	5.73E-09	1.46	3.79E-05

Supplement Figure 5: Pathway analyses of RNA-seq data of scramble vs linc-ADAIN shRNA ASC adipocytes. Top molecular and cellular functions analyzed by Ingenuity Pathway Analysis (A) Top enriched gene ontology (GO) biological process pathways analyzed by DAVID (B).

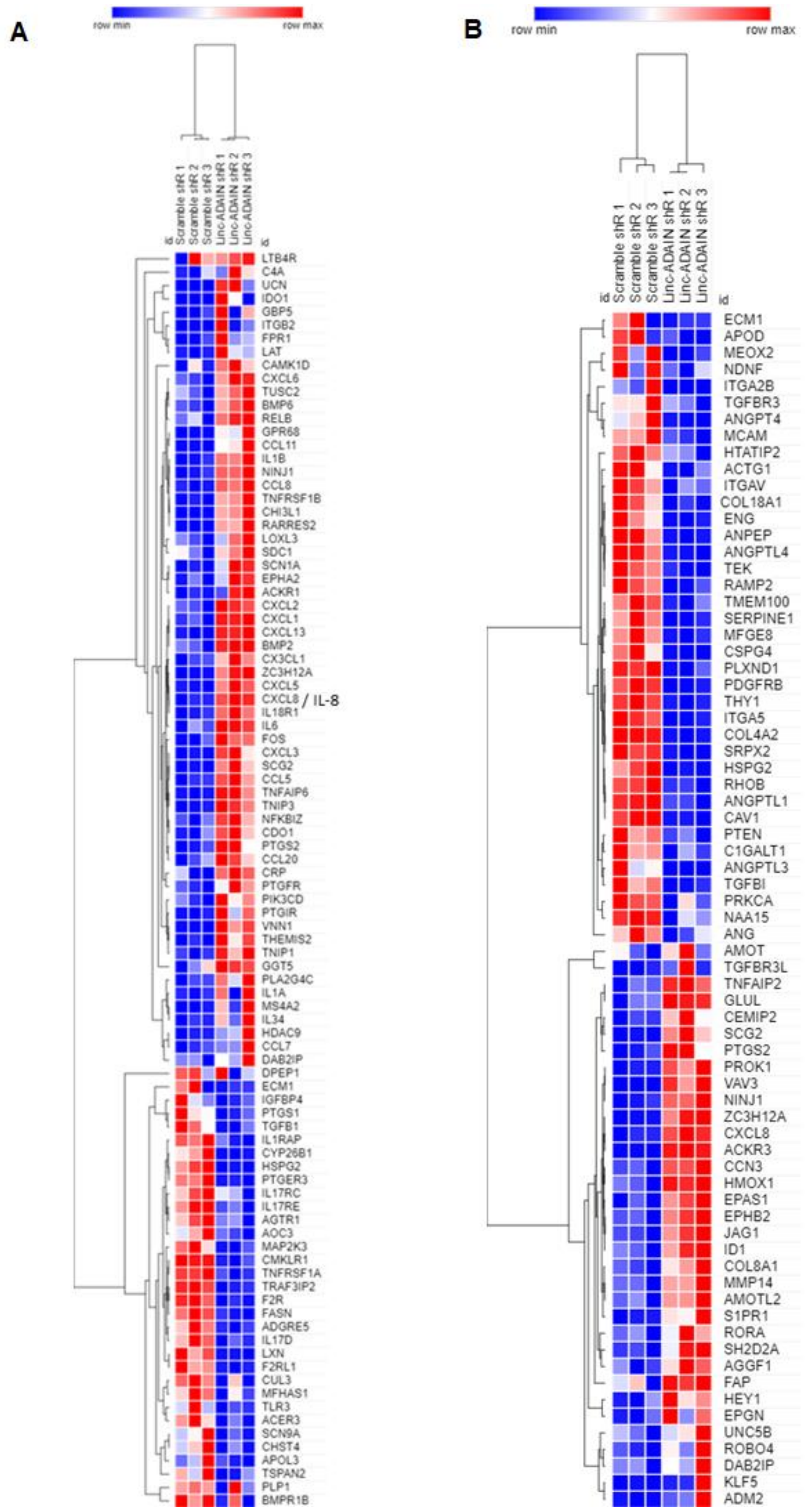

Supplement Figure 6: Differential gene expression analyses of RNA-seq data of scramble vs *linc-ADAIN* shRNA ASC hTERT adipocytes. Heatmap of genes in GO:0006954-inflammatory response (A) and GO:0001525- angiogenesis (B) pathways.

A

		Predicted by RBP map											
		IL8			MCP1			IL6			KLF5		
**Protein**	**Protein Location**	**Z-score**	**P-value**	**# of binding motifs**	**Z-score**	**P-value**	**# of binding motifs**	**Z-score**	**P-value**	**# of binding motifs**	**Z-score**	**P-value**	**# of binding motifs**
**HuR**	Nucleus and Cytoplasm	3.923	0.000	63	3.593	0.000	33	3.418	0.000	40	3.89	0.000	86
**IGF2BP2**	Cytoplasm	3.914	0.000	87	3.086	0.001	21	3.557	0.000	37	4.438	0.000	97
**G3BP2**	Cytoplasm	3.147	0.000	8	2.733	0.003	4	3.04	0.001	9	2.48	0.006	18
**FXR2**	Cytoplasm	3.2	0.000	6	2.657	0.003	1	2.614	0.004	7	2.9	0.001	13
**MATR3**	Nucleus	3	0.001	48	2.554	0.005	16	2.581	0.004	22	3.5	0.000	22
**FMR1**	Cytoplasm	3.403	0.000	6	3	0.001	2	3.194	0.000	8	3.625	0.000	9
**SFPQ**	Nucleus	3.518	0.000	63	3.494	0.0002	22	3.611	0.0001	44	3.723	0.000	86

B

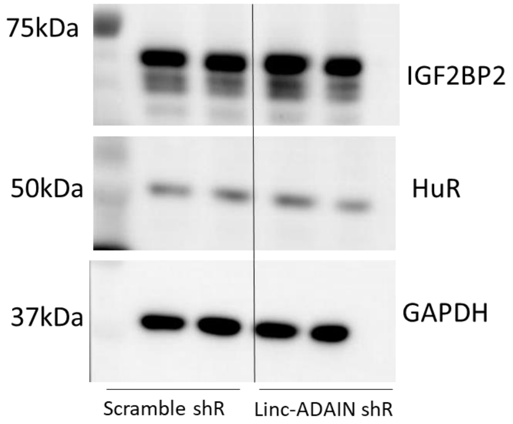

Supplement Figure 7. Predicted RNA binding proteins to *linc-ADAIN, IL-8, MCP-1, IL-6* and *KLF5* RNA. Predicted binding of HuR, IGF2BP2, G3BP2, FXR2, MATR3, FMR1 and SFPQ with *IL-8*, *MCP-1*, *IL-6* and *KLF5* mRNA, using RBPmap (A). Western blot showing total protein expression of IGF2BP2 (top band) and HuR in scramble and *linc-ADAIN* shRNA ASC hTERT adipocytes at day 14 (B).

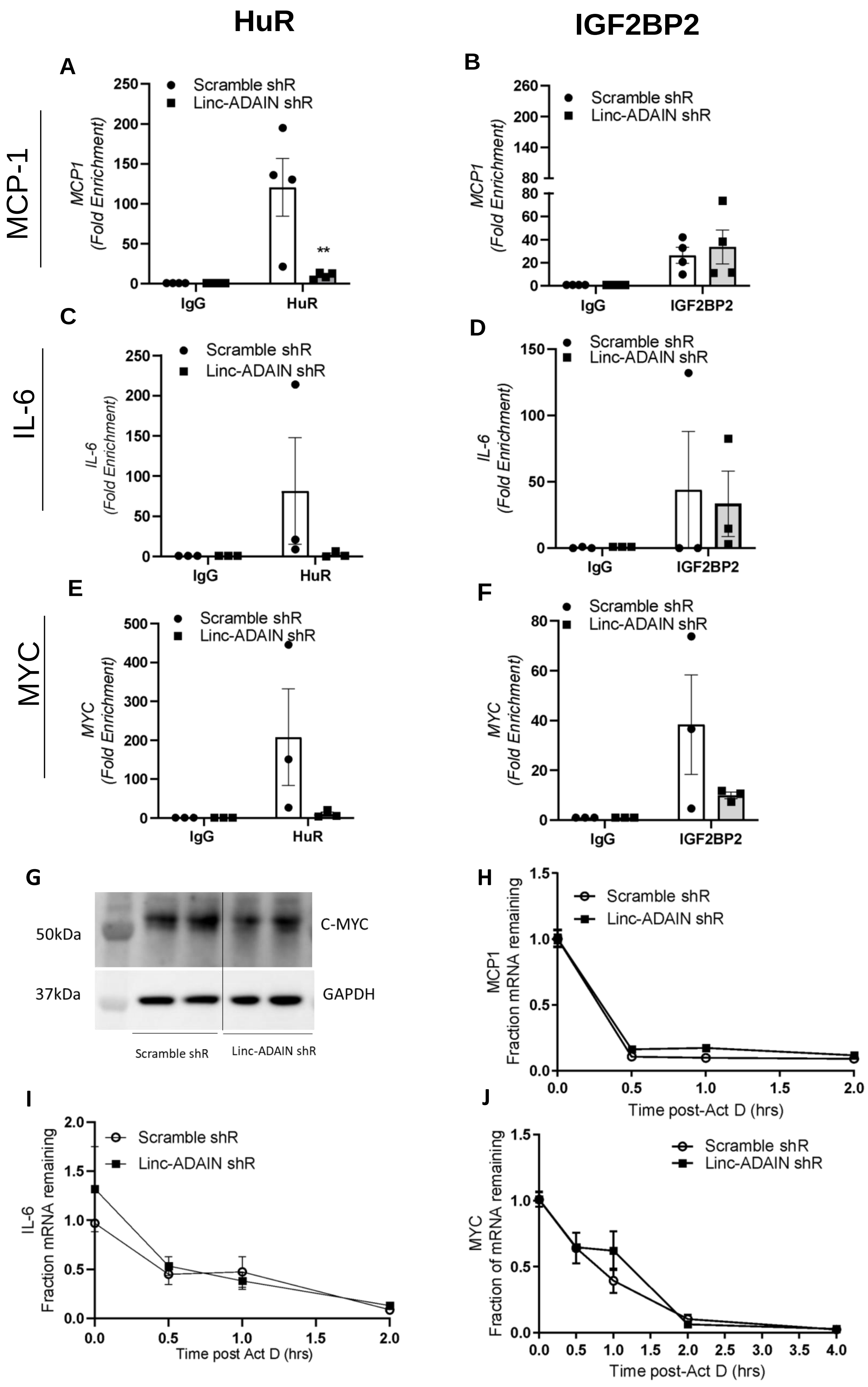
HuR
IGF2BP2
MCP-1
IL-6
MYC
A
B
C
D
E
F
G
H
I
J
Scramble shR
Linc-ADAIN shR
MCP1 (Fold Enrichment)
IL-6 (Fold Enrichment)
MYC (Fold Enrichment)
IgG
HuR
IGF2BP2
**
50kDa
37kDa
C-MYC
GAPDH
Scramble shR
Linc-ADAIN shR
MCP1 Fraction mRNA remaining
IL-6 Fraction mRNA remaining
MYC Fraction of mRNA remaining
Time post-Act D (hrs)
Time post Act D (hrs)

Supplement Figure 8. Knockdown of *linc-ADAIN* has no effect on MCP-1, IL-6 or MYC mRNA stability in adipocytes. RIP assays measure interaction of *MCP-1, IL6* and *MYC* RNA with HuR (A,C,E) and IGF2PBP2 (B,D,F) (N=3). Western blot of c-MYC and GAPDH (G) in total cell lysate of scramble and *linc-ADAIN* shRNA expressing ASC hTERT adipocytes. Scramble and *linc-ADAIN* shRNA expressing ASC adipocytes are treated with Actinomycin-D to halt transcription and gene expression measured at 0.5, 1,2,4 hrs post treatment of *MCP-1* (H), *IL-6* (I) and MYC (J) (N=3). *$p<0.05$, ****$p<0.0001$ w.r.t. Scramble shRNA by Two-Way ANOVA. Data presented as ± SEM.

A

IGF2BP2 RIP -seq HEK293T (Huang et al., 2018)				RNA-seq (O'Reilly)	
	input_fpkm	IP_fpkm	log2_fold_change	log2FoldChange	padj
KLF5	2.17207	266.459	6.9387	2.342851569	2.45E-12
CKB	6.79268	731.398	6.75053	-1.338784679	0.002436
SHC3	0.980199	86.3956	6.46174	2.765955332	2.82E-63
MDK	1.30221	103.159	6.30776	1.623701433	0.010746
MARCKSL1	0.735022	50.7861	6.1105	-1.546004986	2.46E-21
PEMT	0.906179	47.6256	5.7158	-1.279895348	1.35E-16
KIF7	0.554491	24.9756	5.49321	-1.473954504	9.03E-40
SESN2	0.647789	25.3862	5.29238	1.84900647	1.64E-30
SLC7A5	1.20549	37.9904	4.97795	1.485927587	1.4E-07
HOXB9	0.788867	24.6233	4.9641	2.773095577	0.021855
AMOT	3.02988	88.2888	4.8649	2.080302909	0.013361
UCHL1	1.70578	44.2523	4.69725	2.308569022	3.08E-09
PLK1	2.68766	65.6484	4.61034	1.36567595	0.004373
SAMD11	2.07017	48.3633	4.54609	-1.445887132	1.28E-17
COL4A2	1.84746	42.4879	4.52343	-1.451249202	1.29E-94
PGD	1.9669	43.4925	4.46677	-1.41934195	1.32E-39
PTPRF	6.90417	152.596	4.46611	-1.826218547	2.2E-22
COL4A1	0.523423	10.2679	4.29402	-1.540018336	4.66E-96

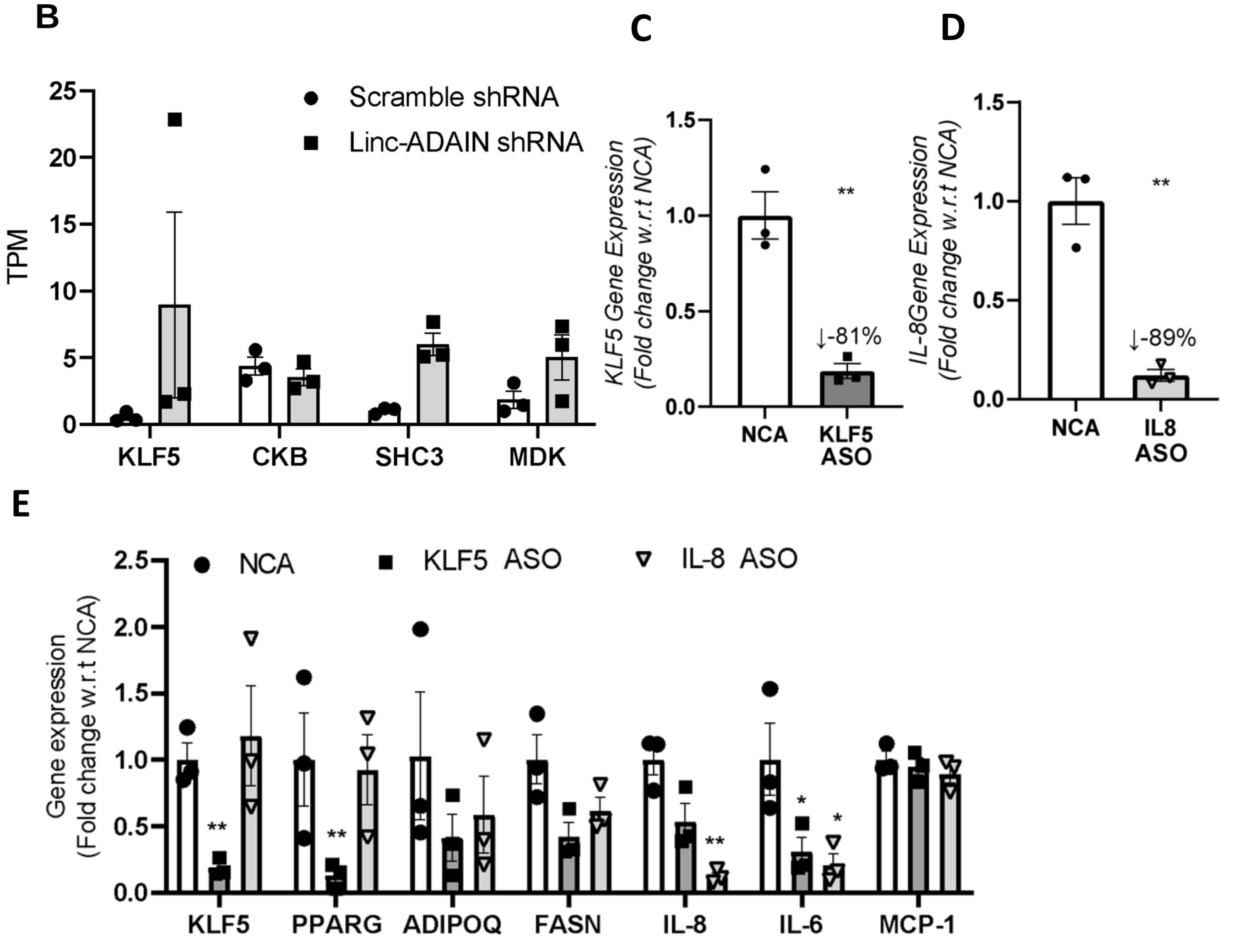

Supplement Figure 9. KLF5 interacts IGF2BP2 and its expressed increased upon *linc-ADAIN* knock-down in adipocytes. We cross referenced IGF2BP2 –RIP seq data set from HEK293T cells differential gene expression data from *linc-ADAIN* vs Scramble shRNA bulk RNA-seq data. Table shows the mRNAs with biggest fold change in IGF2BP2 vs IgG in HEK cells and biggest differential expression in bulk RNA-seq data from *linc-ADAIN* KD experiment. KLF5 was the top target (A). Transcripts per million (TPM) of top 4 mRNAs, *KLF5, CKB, SHC3, MDK* expression in RNA-seq data from scramble vs *linc-ADAIN* shRNA KD in ASC adipocytes (B). Adipocyte Stromal Cell hTERTs (ASC-hTERTs) were differentiated to day 6 then treated with ASO Gapmers designed against IL-8 and KLF5 and a Negative Control A (NCA), at day 6,9 and 12 of adipocyte differentiation. RNA was extracted at Day 14 and genes measured by qPCR KLF5, (C) IL-8, (D) KLF5, PPARG, FASN, IL-8, IL-6 (E) (N=3). **p<0.01, *p>0.05 w.r.t. NCA. Data presented as ± SEM.

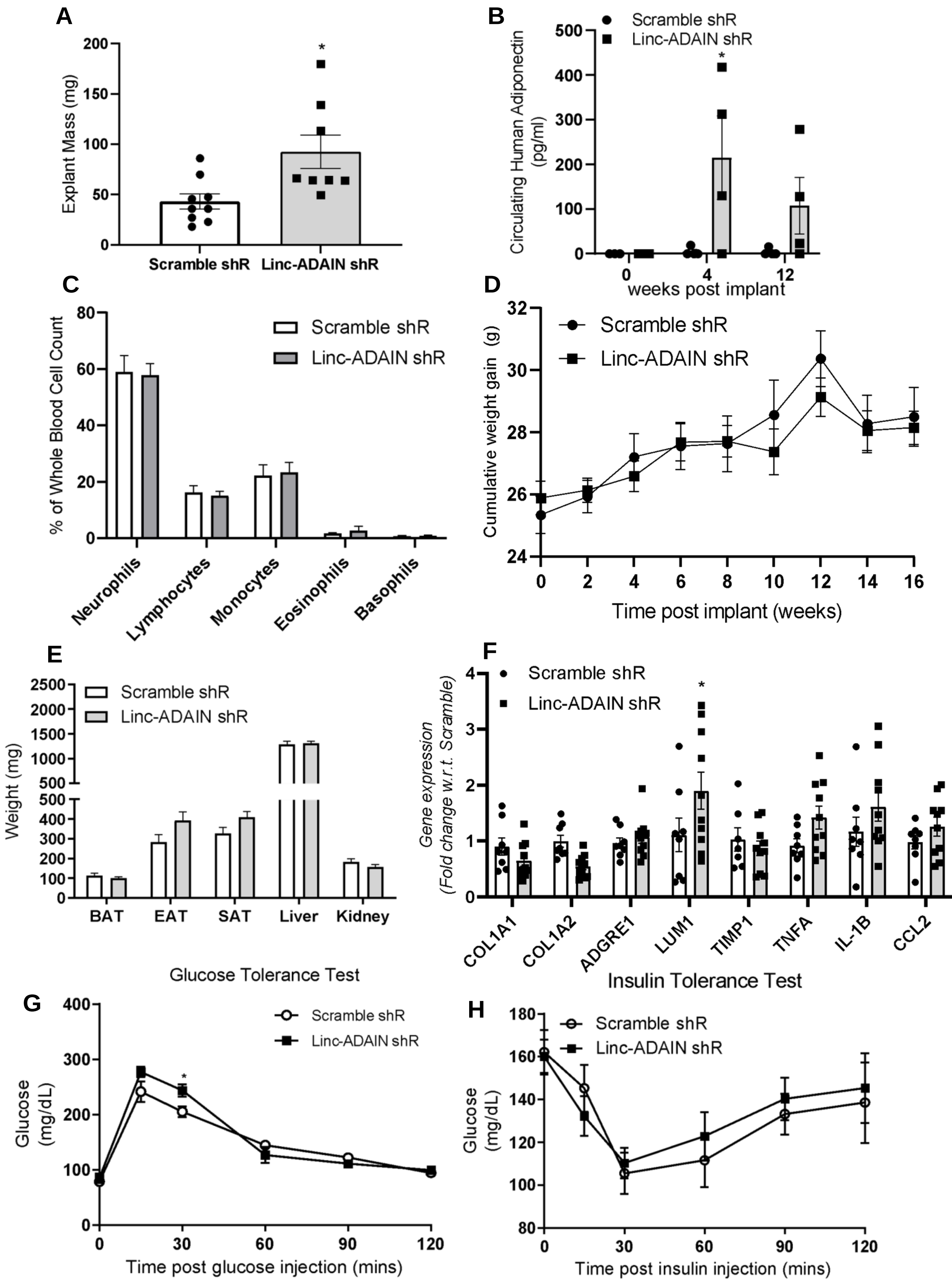
A
Explant Mass (mg)
Scramble shR
Linc-ADAIN shR
*
B
Scramble shR
Linc-ADAIN shR
Circulating Human Adiponectin (pg/ml)
weeks post implant
*
C
% of Whole Blood Cell Count
Scramble shR
Linc-ADAIN shR
Neurophils
Lymphocytes
Monocytes
Eosinophils
Basophils
D
Scramble shR
Linc-ADAIN shR
Cumulative weight gain (g)
Time post implant (weeks)
E
Scramble shR
Linc-ADAIN shR
Weight (mg)
BAT
EAT
SAT
Liver
Kidney
F
Scramble shR
Linc-ADAIN shR
Gene expression (Fold change w.r.t. Scramble)
*
COL1A1
COL1A2
ADGRE1
LUM1
TIMP1
TNFA
IL-1B
CCL2
Glucose Tolerance Test
G
Scramble shR
Linc-ADAIN shR
*
Glucose (mg/dL)
Time post glucose injection (mins)
Insulin Tolerance Test
H
Scramble shR
Linc-ADAIN shR
Glucose (mg/dL)
Time post insulin injection (mins)

Supplement Figure 10. *Linc-ADAIN* shRNA adipocyte implants into NSG mice had little effect on metabolic markers or fatty liver markers compared to Scramble shRNA. Human adipose explant weight after 16 weeks *$p<0.05$ w.r.t. scramble by Mann Whitney U test (A). Circulating human Adiponectin in plasma from mice at 4- and 12-weeks post implant *$p<0.05$ w.r.t. scramble by Two-Way ANOVA (B) (Pooled 4 mice from 4 batch's). Complete Blood cell count (C), Cumulative weight gain (D), Organ weights (E). 16 weeks after mice were implanted, mice were euthanized, and livers collected. RNA was extracted from livers and gene expression of multiple markers (COL1A1, COL1A2, ADGRE1, LUM1, TIMP1, TNFA, IL-1B and CCL2) of fatty liver was measured by qPCR, (N=9 per group), $p<0.05$ w.r.t. scramble shR. (F) 14 weeks after mice were implanted, Mice fasted overnight then received an i.p injection of 1.5g/kg glucose and blood samples collected at times indicated to measure blood glucose. (G). Mice fasted for 5 hours then received an i.p injection of 0.5U/kg of insulin and blood samples collected at times indicated to measure blood glucose. (H) N= 6 for scramble shR, N=4 for Linc-ADAIN shR. Data presented as ± SEM.
