# Supplementary material for: linc-ADAIN, a human adipose lincRNA, regulates adipogenesis by modulating KLF5 and IL-8 mRNA stability
Source: Cell Rep. Author manuscript; Available in PMC 2024 Aug 20. (PMC11334222; doi:10.1016/j.celrep.2024.114240)
Supplement: Supplementary Material [file NIHMS2009043-supplement-Supplementary_Material.zip › 1-s2.0-S2211124724005680-mmc7.pdf]

Cell Reports

Article

linc-ADAIN, a human adipose lincRNA, regulates adipogenesis by modulating KLF5 and IL-8 mRNA stability

Graphical abstract

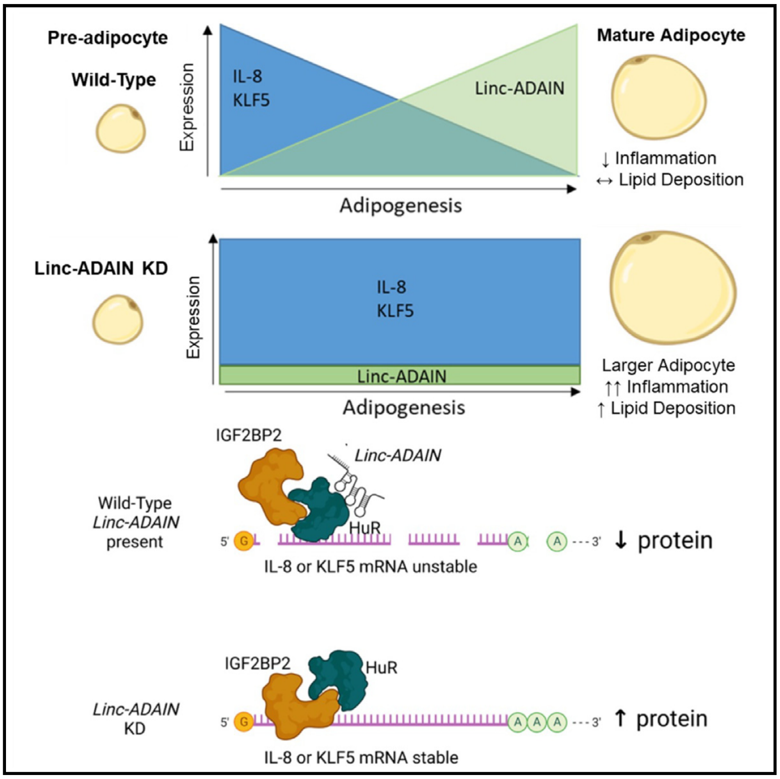

Highlights

- *linc-ADAIN* expression increases during adipocyte differentiation
- *linc-ADAIN* is suppressed in white adipose tissue of obese humans
- Knockdown of *linc-ADAIN* leads to increased inflammation and sustained adipogenesis
- *linc-ADAIN* knockdown increases IL-8 and KLF5 mRNA, important genes for adipogenesis

Authors

Marcella E. O'Reilly, Sebastian Ho, Johana Coronel, ..., Silvia Corvera, Susan K. Fried, Muredach P. Reilly

Correspondence

mpr2144@cumc.columbia.edu

In brief

O'Reilly et al. investigate a long non-coding RNA, named linc-adipose anti-inflammatory (*linc-ADAIN*), and its role in adipose inflammation and maturation of fat cells (adipocytes). They show that *linc-ADAIN*'s expression is suppressed in adipose tissue of obese humans and that *linc-ADAIN* regulates inflammation as well as maturation and size of fat cells.

O'Reilly et al., 2024, Cell Reports *43*, 114240

May 28, 2024 © 2024 The Authors. Published by Elsevier Inc.

https://doi.org/10.1016/j.celrep.2024.114240

Cell Reports

CellPress
OPEN ACCESS

Article

linc-ADAIN, a human adipose lincRNA, regulates adipogenesis by modulating KLF5 and IL-8 mRNA stability

Marcella E. O'Reilly,[1] Sebastian Ho,[1] Johana Coronel,[1] Lucie Zhu,[1] Wen Liu,[1] Chenyi Xue,[1] Eunyoung Kim,[1] Esther Cynn,[1] Caio V. Matias,[1] Rajesh Kumar Soni,[2] Chen Wang,[3] Iuliana Ionita-Laza,[3] Robert C. Bauer,[1] Leila Ross,[1] Yiying Zhang,[4] Silvia Corvera,[5] Susan K. Fried,[6] and Muredach P. Reilly[1,7,8,*]

[1]Cardiometabolic Genomics Program, Division of Cardiology, Department of Medicine, Columbia University Medical Center, New York, NY, USA
[2]Proteomics and Macromolecular Crystallography Shared Resource, Herbert Irving Comprehensive Cancer Center, Columbia University Medical Center, New York, NY, USA
[3]Department of Statistics, Mailman School of Public Health, Columbia University Medical Center, New York, NY, USA
[4]Division of Molecular Genetics, Department of Pediatrics, Columbia University Medical Center, New York, NY, USA
[5]Program in Molecular Medicine, University of Massachusetts Medical School, Worcester, MA 01605, USA
[6]Diabetes, Obesity, and Metabolism Institute, Icahn School of Medicine at Mount Sinai, New York, NY 10029, USA
[7]Irving Institute for Clinical and Translational Research, Columbia University, New York, NY 10032, USA
[8]Lead contact
*Correspondence: mpr2144@cumc.columbia.edu
https://doi.org/10.1016/j.celrep.2024.114240

SUMMARY

Adipose tissue remodeling and dysfunction, characterized by elevated inflammation and insulin resistance, play a central role in obesity-related development of type 2 diabetes (T2D) and cardiovascular diseases. Long intergenic non-coding RNAs (lincRNAs) are important regulators of cellular functions. Here, we describe the functions of *linc-ADAIN* (adipose anti-inflammatory), an adipose lincRNA that is downregulated in white adipose tissue of obese humans. We demonstrate that *linc-ADAIN* knockdown (KD) increases KLF5 and interleukin-8 (IL-8) mRNA stability and translation by interacting with IGF2BP2. Upregulation of KLF5 and IL-8, via *linc-ADAIN* KD, leads to an enhanced adipogenic program and adipose tissue inflammation, mirroring the obese state, *in vitro* and *in vivo*. KD of *linc-ADAIN* in human adipose stromal cell (ASC) hTERT adipocytes implanted into mice increases adipocyte size and macrophage infiltration compared to implanted control adipocytes, mimicking hallmark features of obesity-induced adipose tissue remodeling. *linc-ADAIN* is an anti-inflammatory lincRNA that limits adipose tissue expansion and lipid storage.

INTRODUCTION

Obesity is a state of chronic low-grade inflammation that increases the risk for cardiometabolic diseases (CMDs), a cluster of conditions that include cardiovascular disease (CVD) and type 2 diabetes (T2D).[1,2] Remodeling and dysfunction of adipose tissue, characterized by increased adipocyte hypertrophy, macrophage recruitment, inflammation, hypoxia, and insulin resistance, play a central role in the development of CMD.[3,4] Despite substantial scientific and clinical advances, CMDs continue to be major causes of morbidity and mortality.[1,5] Therefore, to drive new discoveries and treatments, it is imperative to investigate all potential contributions to this complex disease clustering.

Genomic discoveries have advanced our understanding of adipose metabolic functions.[6] Regions of the genome that were once thought to be transcriptionally silent are now known to be transcribed into a variety of regulatory non-coding RNAs,[7] including long (>200 nt) intergenic non-coding RNAs (lincRNAs), most of which remain poorly understood.[8,9] In the last decade, lincRNAs have emerged as key regulators of cell-specific functions that can fine-tune metabolism within adipose tissue[10] and other tissues.[11,12] Several adipose lincRNAs have been studied to date,[10] advancing the field of adipose biology and obesity. For example, a nuclear long non-coding RNA (lncRNA), HOTAIR, expressed in human gluteal but not abdominal subcutaneous adipose tissue, can impact transcriptional regulation of adipogenic genes, such as PPARγ and LPL, via DNA methylation.[13,14] Human-specific thermogenic adipose lincRNA, LINC00473, is decreased in obesity and T2D and regulates lipolysis and genes associated with mitochondrial oxidative metabolism, impacting energy metabolism in adipocytes.[15] We have previously identified *linc-ADAL*, a non-conserved human adipose lincRNA, as upregulated in human subcutaneous obese white adipose tissue and to modulate *de novo* lipogenesis.[16] *linc-ADAL*, found equally in cytoplasm and nucleus, interacts with hnRNPU in the nucleus and IGF2BP2 in the cytoplasm to regulate mRNA

 © 2024 The Authors. Published by Elsevier Inc.
This is an open access article under the CC BY license (http://creativecommons.org/licenses/by/4.0/).

and protein levels of a subset of metabolic genes in adipocytes. Here, we investigate human adipocyte *linc-ADAIN* (adipose anti-inflammatory; also known as *linc-DMRT2*[17] and as linc01230[18]), previously identified by our laboratory,[17] because of its enriched expression in mature adipocytes and downregulation in human obese white adipose tissue. *linc-ADAIN*, also known as linc01230, has been shown to be expressed in endothelial cells.[19] *linc-ADAIN* negatively correlates with total percentage of body fat in obese humans. Therefore, we hypothesize that *linc-ADAIN* may play a role in obese adipose tissue remodeling. Mirroring its downregulation in human obese white adipose tissue, we show that knockdown (KD) of *linc-ADAIN* in human adipocytes led to larger lipid-filled adipocytes and increased secretion of adipocyte cytokines. This was due to an enhancement of the adipogenic program via stabilization of KLF5 mRNA, an early adipogenic transcription factor,[20] and IL-8, likely via interaction of IGF2BP2 and human antigen R (HuR), two proteins known to form a complex to stabilize mRNAs.[21,22] *linc-ADAIN* has a syntenic locus in mouse. However, unlike in humans, the mouse lincRNA (2610016A17Rik) at this locus differs in its regulation by PPARγ, has a reverse orientation, and shares a promoter with its neighboring protein-coding gene, *Dmrt2*. To circumvent uncertainty in conservation between mouse and human and the challenge to generating *linc-ADAIN* gene deletion in a mouse model, we adapted our collaborator's established transplant mouse model for *in vivo* studies of human *linc-ADAIN*.[23,24] Human adipose stromal cell (ASC) hTERT adipocytes overexpressing a short hairpin RNA (shRNA) targeting *linc-ADAIN* were implanted into the flanks of NOD.Cg-PrkdcscidIl2rgtm1Wjl/SzJ (NOD-scid IL2rγnull, NSG) mice to generate a functional human fat pad *in vivo*. Subsequent explant of mature implants showed that *linc-ADAIN* KD had increased adipocyte size and macrophage infiltration compared to control, mimicking hallmark features of adipose tissue remodeling seen in obesity.

RESULTS

Adipose-enriched *linc-ADAIN* expression is modulated by obesity in humans

linc-ADAIN (previously named linc-DMRT2) was identified as an inflammatory modulated lincRNA by our laboratory through deep RNA sequencing (RNA-seq) of gluteal subcutaneous white adipose tissue (sWAT) from healthy, non-obese volunteers in the Genetics and Evoked Responses to Niacin and Endotoxemia (GENE) study.[17] Adipose expression of lincRNAs, including *linc-ADAIN*, was validated in lean healthy subjects from GENE (N = 25) and obese subjects from the Penn Human Adipose Resource (HAR) (N = 39). Here, we show that *linc-ADAIN* expression is reduced in sWAT of obese compared to lean humans (Figure 1A), with similar expression in sWAT and visceral adipose tissue (VAT) (Figure 1B) and slightly higher expression in males than in females (Figure 1C). *linc-ADAIN* expression negatively correlates with percentage of total body fat (Figure 1D). During endotoxemia, *linc-ADAIN* expression was significantly downregulated in human sWAT (Figure 1E), demonstrating that its adipose expression is suppressed in this well-studied model of acute systemic inflammation.

Human *linc-ADAIN* expression is induced during adipocyte differentiation and is modulated by canonical adipocyte transcription factors such as PPARγ

The *linc-ADAIN* gene is located upstream of the protein-coding gene DMRT2 on human chromosome 9. RNA-seq, 3′ RACE (rapid amplification of cDNA ends) (Figure S1A), and the 5′ transcription start site (TSS) identified by cap analysis of gene expression (CAGE)[25] in human adipocytes suggest that *linc-ADAIN* has three alternatively spliced isoforms, with isoforms 1 and 2 sharing the same promoter (Figures 2A and S2A). Isoform 3 is the most abundant and therefore is the predominant focus for follow-on studies. Analysis of the phylogenetic information–based codon substitution frequency (PhyloCSF/Expasy) tracks on the UCSC genome browser[26] suggested that the three *linc-ADAIN* transcripts had low probabilities of containing open reading frames for coding sequences. *In vitro* transcription/translation assays failed to detect any peptide products, supporting its predicted non-coding status (Figure S1B). Tissue expression of *linc-ADAIN* using RNA-seq data from GTEx (Genotype-Tissue Expression project) shows that *linc-ADAIN* is predominantly expressed in adipose and kidney tissues (Figure 2B). Previously, we showed that *linc-ADAIN* was detected in adipocytes, but not in monocytes, unpolarized macrophages, or macrophages polarized to M1 or M2 by qPCR.[17] Using the Broad Institute online single-cell RNA-seq in adipose tissue dataset from Emont et al.,[27] expression of *linc-ADAIN*, listed as *linc01230*, is almost exclusively limited to adipocytes and not other cell types within human WAT (Figure 2G). Human adipocyte chromatin immunoprecipitation (ChIP)-seq data revealed high occupancy of H3K4me3 at the *linc-ADAIN* locus, with multiple PPARγ and C/EBPα binding peaks, and H3K4me and H3K27ac histone enhancer marks upstream of its TSS (Figure 2A). In mature adipocytes, *linc-ADAIN* expression was reduced by GW9662, a PPARγ antagonist (Figure S1C). Conversely, the PPARγ agonist rosiglitazone induced its expression (Figure 2C). *linc-ADAIN* was slightly expressed in ASC pre-adipocytes but was markedly induced during ASC differentiation into adipocytes (Figure 2D). The *linc-ADAIN* locus is syntenic in mouse as defined by homologous neighboring protein-coding genes. However, unlike human *linc-ADAIN*, the 2610016A17Rik lincRNA transcript upstream of DMRT2 in mouse is on the antisense strand, is not regulated by PPARγ in the same manner as human *linc-ADAIN*, and shares a promoter region with mouse DMRT2 (Figure S1D). Overall, these data show that human *linc-ADAIN* is exclusively expressed in adipocytes in WAT, is markedly enriched in WAT over other tissues, is directly regulated by the canonical adipose transcription factor PPARγ, and most likely is not conserved.

linc-ADAIN is located predominantly in the cytoplasm

Following fractionation of mature primary human ASC adipocytes, *linc-ADAIN* transcripts are found mostly in the cytoplasm (Figures 2E and S2B). RNA scope confirms *linc-ADAIN* isoforms to be mostly cytoplasmic, often in perinuclear locations, particularly the most abundant isoform, 3 (Figure 2F). Using lentiviral delivery of an shRNA to target all three isoforms in human hTERT adipocytes (Figure S2D), ∼85% KD of *linc-ADAIN* isoforms was achieved compared to the scramble shRNA (Figure S1E).

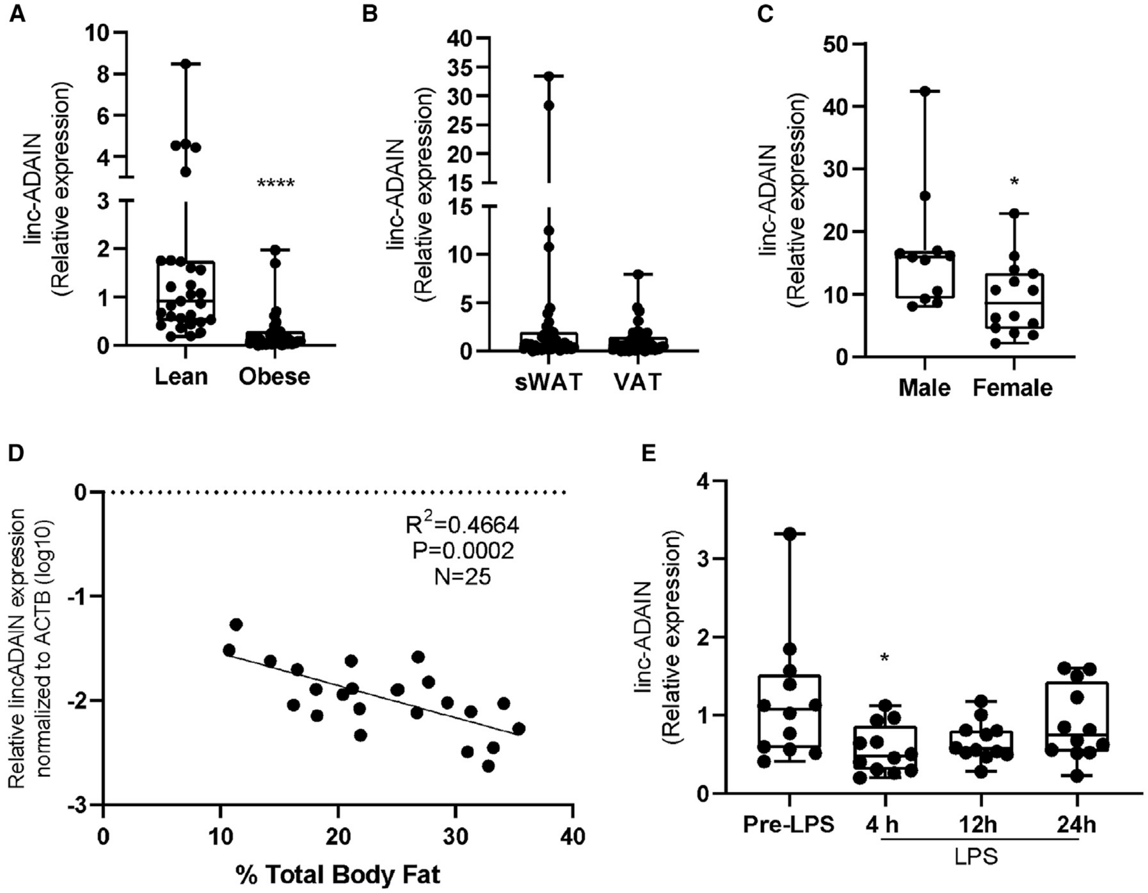

Figure 1. Adipose-enriched *linc-ADAIN* expression is modulated by obesity in humans

(A) Gluteal sWAT expression of *linc-ADAIN* is reduced in obese humans ($N = 29$ [lean], $N = 39$ [obese]); ****$p < 0.0001$ with relation to lean by Mann-Whitney U test.

(B) *linc-ADAIN* expression in human subcutaneous or visceral adipose tissue ($N = 39$ [obese]); $p = 0.2119$ by Mann-Whitney U test.

(C) *linc-ADAIN* expression in subcutaneous adipose tissue in males ($N = 11$) vs. females ($N = 14$); *$p < 0.05$ with relation to male by Mann-Whitney U test.

(D) *linc-ADAIN* expression correlates negatively with total percentage of body fat; Pearson correlations.

(E) Experimental human endotoxemia suppresses *linc-ADAIN* expression in human sWAT ($N = 12$); *$p < 0.05$ with relation to pre-lipopolysaccharide (LPS) by one-way ANOVA. Data are presented as the mean ± SEM.

linc-ADAIN KD had no effect on the expression of nearby protein-coding genes ± 500 kb of the *linc-ADAIN* locus, e.g., DMRT2, DMRT1, or DMRT3 (Figure S1F) or DMRT2 protein (Figure S2C).

linc-ADAIN KD increases lipid accumulation, the adipogenic program, and cytokine secretion in human adipocytes

Due to *linc-ADAIN*'s association with human obesity, we tested whether *linc-ADAIN* regulated human adipocyte differentiation and lipid storage. KD of *linc-ADAIN* via shRNA increased cell size and lipid storage, measured by Nile red staining and cellular triglyceride levels, in ASC hTERT adipocytes (Figures 3A, 3B, and 3C) at 14 and 21 days post differentiation. KD of *linc-ADAIN* also increased mRNA (Figure 3D) and protein expression (Figure 3E) of adipogenic markers PPARγ, FASN, ATGL (Pnpla2), perilipin 1, FABP4, GLUT4, C/EBPα, and adiponectin and increased adipocyte secretion of adiponectin at day 14 of differentiation (Figure S3A). Levels of multiple RNAs that regulate adipocyte lipolysis and *de novo* lipogenesis, including ATGL, HSL, MGL, and MTP, were increased by KD of *linc-ADAIN* at day 14 of adipocyte differentiation (Figure S3C).

Because adipose tissue inflammation modulated its expression, we tested whether *linc-ADAIN* regulated inflammatory pathways in human adipocytes. Using a human cytokine array (C5 Ray Biotech, 80 cytokines) as an initial screen, we found that, compared to scramble control, *linc-ADAIN* KD tended to increase adipocyte secretion of several cytokines, including IL-8, MCP-1, IL-6, M-CSF, and MIP-1 (Figures S3E and S3F). Using a human adipocyte panel Luminex assay, we confirmed that *linc-ADAIN* KD increased adipocyte secretion of IL-8, MCP-1, and IL-6 (Figure 3F) and validated this by ELISA (Figures S4A and S4B). Suggestive of post-translational regulation, of these, only IL-8 had significant increases at the mRNA level, and this was to a much lesser extent (4-fold) than increases in secreted IL-8 protein (20-fold) (Figures 3G and S4B). KD of *linc-ADAIN* tended to increase IL-1β and TNF-α mRNA transcripts but did not increase IL-1β or TNF-α protein secretion (Figures 3F and

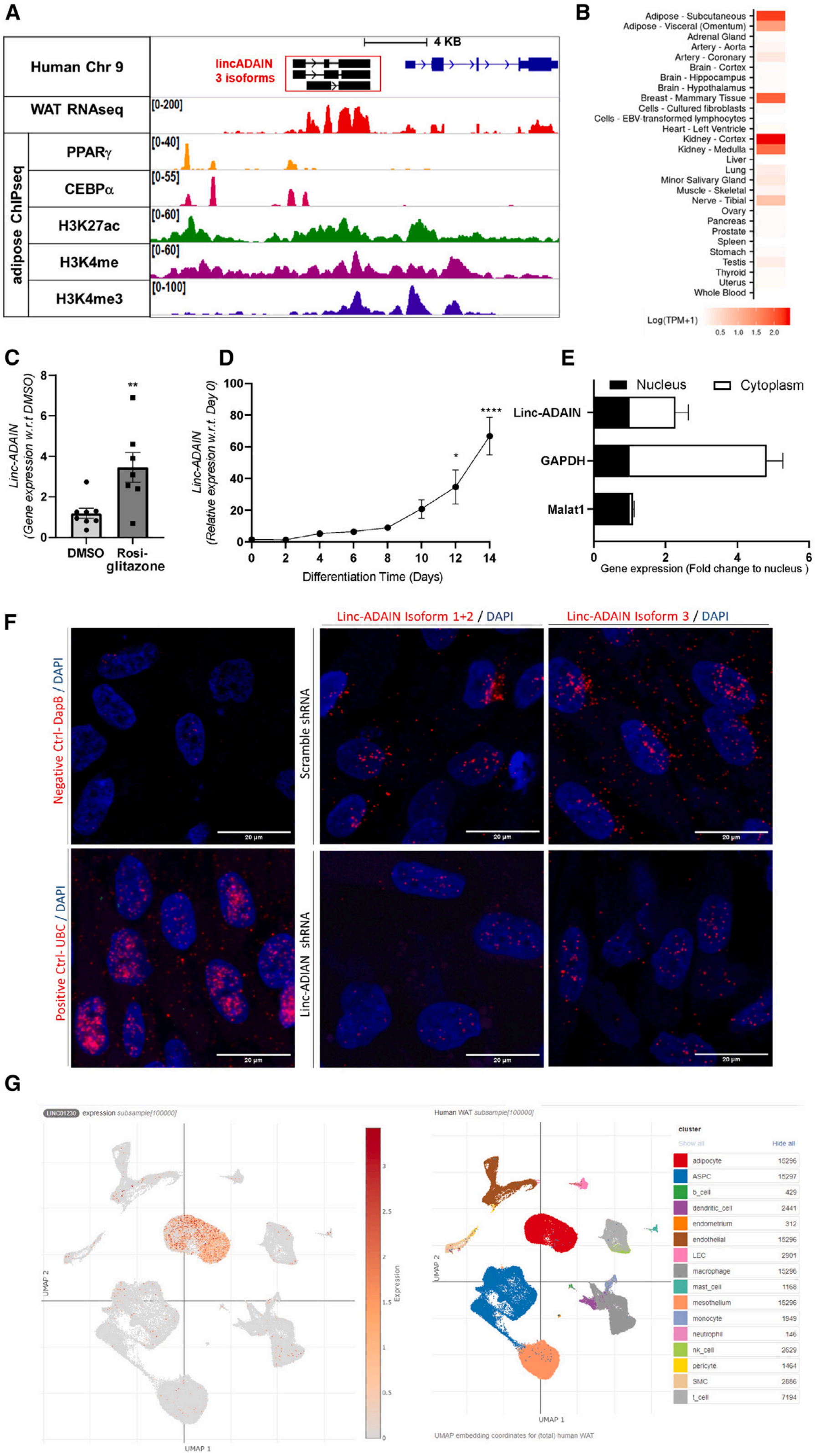

(legend on next page)

3G), known to be very low at baseline in adipocytes.[28] Cytokine findings were consistent in independent experiments using an antisense oligonucleotide (ASO) against *linc-ADAIN* and a negative control (Figure S3F to S4F). Ingenuity Pathway Analysis (IPA) and gene ontology (GO) enrichment analysis of RNA-seq data from *linc-ADAIN* KD vs. scramble control 14-day adipocytes showed cellular movement as a top molecular function (Figure S5A) and inflammatory response and angiogenesis as top enriched GO biological processes (Figures S5B, S6A, and S6B) for genes enriched in *linc-ADAIN* KD vs. scramble control.

linc-ADAIN interacts with HuR and IGF2BP2 in human adipocytes

We screened for candidate *linc-ADAIN*-protein interactions as potential functional mediators. First, RBPmap[29] was used to predict RNA binding protein (RBP) motifs within the *linc-ADAIN* RNA sequence. A total of 92 proteins were predicted to have binding with isoform 3, the most abundant *linc-ADAIN* (Table S2). Second, a biotinylated pull-down assay of *linc-ADAIN* coupled to mass spectrometry (MS) in primary ASC adipocytes identified 118 proteins that had a ≥5-fold total spectrum count (TSC) bound to *linc-ADAIN* compared to antisense control (Table S1). Since we were interested in how *linc-ADAIN* may regulate specific cytokine mRNAs, we used RBPmap to predict binding proteins that were common for IL-8, MCP-1, and IL-6 (Tables S3–S5) as well as for *linc-ADAIN*. We then overlapped the five datasets, which included (1) *linc-ADAIN* MS-pull-down data and the RBPmap-predicted proteins for (2) *linc-ADAIN*, (3) IL-8, (4) MCP-1, and (5) IL-6 (Tables S1–S5), and we found seven proteins in common: HuR, IGF2BP2, G3BP2, FXR2, MATR3, FMR1, and SFPQ (Figures 4A, 4B, and S7A). STRING analysis of the seven proteins showed regulation of translation and mRNA stability as top biological processes (Figure 4C), and two of these proteins (IGF2BP2 and HuR [ELAV1]) are found in the coding region instability determinant complex, which stabilizes mRNAs to protect them from degradation.[21,30] Specific proteins involved in mRNA stability are HuR (ELAVL1), IGF2BP2, FMR1, and FXR2 (Figure 4D). HuR and IGF2BP2 had the highest *Z* score and number of predicted binding motifs for *linc-ADAIN* of the seven proteins (Figure 4B). For this reason, we chose to validate their interactions with *linc-ADAIN*. Using RNA immunoprecipitation (RIP) assays, we found that *linc-ADAIN* RNA interacted with HuR protein in mature ASC adipocytes (Figure 4E) but not IGF2BP2 (data not shown). However, consistent with previously reported interactions between IGF2BP2 and HuR proteins,[21,30] we found that IGF2BP2 and HuR interact in human hTERT ASC adipocytes, as shown by western blot of an immunoprecipitation pull-down of IGF2BP2 probed for HuR protein (Figure 4F). Combining RNA scope and immunofluorescence (IF), we observed co-localization of *linc-ADAIN* with HuR and IGF2BP2 in adipocyte perinuclear regions (Figure 4G).

KD of *linc-ADAIN* stabilizes IL-8 and KLF5 mRNA, likely through IGF2BP2

We next investigated how *linc-ADAIN* might modulate HuR and IGF2BP2 functions. KD of *linc-ADAIN* via shRNA had no effect on IGF2BP2 or HuR protein expression in mature ASC adipocytes (Figure S7B). RIP assays showed that KD of *linc-ADAIN* affected binding of mRNAs to both HuR and IGF2BP2 (Figure 5). For IL-8 and KLF5, *linc-ADAIN* KD blunted the interaction with HuR (Figures 5A and 5C), but increased binding with IGF2BP2 (Figure 5B). With linc-ADAIN KD, there was enrichment of IL-8 and KLF5 mRNA on IGF2BP2 protein (Figures 5B and 5D). The interaction of MCP-1 and HuR was blunted by *linc-ADAIN* KD, whereas the MCP1-IGF2BP2 interaction appeared unchanged (Figures S8A and S8B). c-myc mRNA, known to be modulated by IGF2BPs,[30] also had reduced binding to HuR and IGF2BP2 upon *linc-ADAIN* KD (Figures S8E and S8F) without an effect on c-Myc protein expression (Figure S8G). Negligible effects were observed for IL-6 (Figures S8C and S8D) and GAPDH, an mRNA not known to be modulated by the mRNA stabilizing coding region instability determinant complex (Figures 5E and 5F).

Given the roles of HuR and IGF2BP2 in mRNA stabilization and in adipogenesis,[22,30–32] we next explored whether mRNA stabilization of specific targets, such as IL-8 and KLF5, might mediate *linc-ADAIN* effects on adipogenic gene expression noted above. We cross-referenced mRNAs with the greatest fold change in IGF2BP2 binding vs. IgG in HEK293T cells from public data[21] and mRNAs with the greatest differential expression in our bulk RNA-seq data from *linc-ADAIN* shRNA vs. scramble shRNA in 14-day ASC adipocytes (Figure S9A). The top candidate mRNA was KLF5, a transcription factor shown to be a key regulator of early adipogenesis[20] and also found in the angiogenesis GO pathway above (Figure S6B). KLF5 mRNA was upregulated by *linc-ADAIN* KD (Figure S9B) and showed the same pattern of binding to HuR and IGF2BP2 as IL-8 (Figures 5C and 5D), indicating that KLK5 and IL-8 mRNAs may be regulated similarly.

We investigated the effect of *linc-ADAIN* KD on mRNA stability in mature ASC hTERT adipocytes. Using actinomycin-D to pause transcription, *linc-ADAIN* KD delayed IL-8 and KLF5 mRNA decay responses (Figures 5G and 5H), suggesting that endogenous *linc-ADAIN* accelerates the decay of these two

Figure 2. Human *linc-ADAIN* expression is induced during adipocyte differentiation and is modulated by the canonical adipocyte transcription factor PPARγ

(A) Regulatory features at the *linc-ADAIN* locus, RNA-seq coverage (human adipocytes), transcription factor binding, and active histone modification markers.

(B) Tissue expression of *linc-ADAIN* from GTEx (gene mean transcripts per million [TPM]).

(C and D) Induction of *linc-ADAIN* by the PPARγ agonist rosiglitazone (10 μM) (C) and during adipocyte differentiation *in vitro* (D) (*N* = 3 in triplicate).

(E) Cellular fractionation of primary ASC adipocytes. qPCR of MALAT1, GAPDH, and linc-ADAIN of nuclear and cytoplasmic fractions. Data were normalized by averaging of loading controls GAPDH, β-ACTIN, MALAT1, U6, and HPRT and then subtracting the nucleus value and getting a fold change of gene expression compared to nucleus (*N* = 3).

(F) RNA scope assay showing the spatial expression of *linc-ADAIN* (red) and nuclei (DAPI/blue) in scramble and linc-ADAIN shRNA hTERT ASC adipocytes (scale bar, 20 μm).

(G) UMAP (uniform manifold approximation and projection) projection of *linc-ADAIN* (linc01230) expression in single-cell RNA-seq of human subcutaneous WAT (Broad Institute[27]). Data are presented as the mean ± SEM.

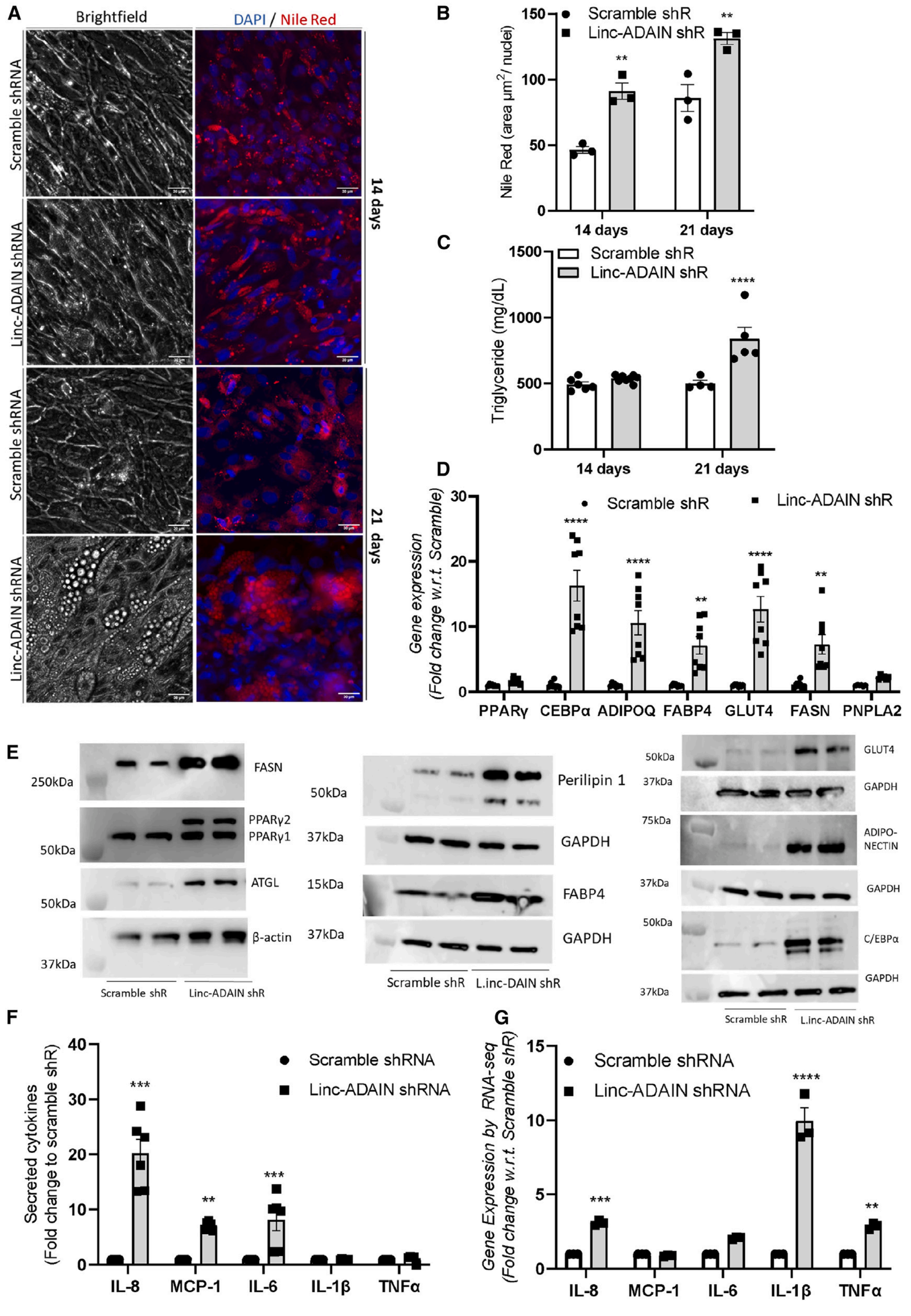

(legend on next page)

mRNAs in maturing adipocytes. *linc-ADAIN* KD did not affect GAPDH, MCP1, or IL-6 mRNA stability (Figures 5I, S8H, and S8I), but did cause a modest delay in the MYC (Figure S8J) mRNA decay response. *linc-ADAIN* KD enhanced IGF2BP2 binding to IL-8 and KLF5 but not to MCP-1 or IL-6, suggesting that IGF2BP2 may mediate *linc-ADAIN* induced differences in patterns of IL-8 and KLF5 but not MCP-1 or IL-6 mRNA and protein. Overall, because of its established role in mRNA stabilization,[33–35] IGF2BP2 may be a mediator of IL-8 and KLF5 stabilization observed during *linc-ADAIN* KD.

KD of *linc-ADAIN* prevents the normal reduction of KLF5 expression during late adipocyte differentiation

As KLF5 is an important transcription factor in early adipogenesis,[20] we investigated its expression during adipocyte differentiation in the presence and absence of *linc-ADAIN.* Normally, *linc-ADAIN* is induced gradually as adipocytes differentiate (Figure 2D), whereas KLF5 expression increases during early differentiation but falls as adipocytes mature (Figures 6B and 6E, scramble).[20] *linc-ADAIN* KD prevented the drop in KLF5 mRNA (Figures 6A and 6B) and protein (Figure 6E) during later adipocyte differentiation, with marked upregulated expression at day 14 compared to absent KLF5 expression in controls at these later time points.[20] This *linc-ADAIN*-KD-induced persistence of KLF5 expression was associated with increased expression of PPARγ1 and 2 (Figure 6E) and CEBPα at day 14 (Figure 6D). Consistent with published data,[36] IL-8 mRNA expression is high at baseline in pre-adipocytes, and levels decline as adipocytes differentiate (Figure 6F, scramble). Silencing of *linc-ADAIN* before differentiation led to increased IL-8 mRNA at baseline (Figure 6F) and a sustained increase in IL-8 secretion throughout differentiation (Figure 6G). To test if KLF5 and IL-8 could mediate the adipogenic and inflammatory marker phenotypes observed with linc-ADAIN KD, we performed ASO-induced KD of KLF5 and IL-8 in ASC hTERTs, achieving 81% and 89% KD, respectively (Figures S9C and S9D). We observed significant reductions in PPARγ and IL-6 with KLF5 KD (Figure S9E). Indeed, KD of KLF5 had the opposite effect on PPARγ and IL-6 compared to *linc-ADAIN* KD (Figures S3D and S3F). These findings support the concept that linc-ADAIN may control adipogenesis through KLF5. IL-8 KD showed no changes in adipogenic genes such as KLF5 or PPARγ—this was expected because IL-8 is not known to regulate these factors. However, there was a significant reduction in IL-6 gene expression with IL-8 KD. Again, this is in the opposite direction of the *linc-ADAIN* KD effect on IL-6 (see Figure 3F).

Overall, our data suggest that the sustained increase in KLF5 and IL-8 mRNA induced by *linc-ADAIN* KD during adipocyte differentiation is mediated by IGF2BP2 stabilization of their mRNA. Sustained expression of KLF5 is likely to drive increased expression of adipogenic transcription factors, PPARγ and CEBPα, as has been reported,[20,37] and increased adipocyte lipid accumulation.

Adipocytes with reduced *linc-ADAIN* have increased size and lipid storage and greater macrophage infiltration *in vivo*

Next, we sought to determine the impact of the depletion of *linc-ADAIN* on adipocyte maturation, lipid storage, and KLF5 and IL-8 expression *in vivo*. Although, the human *linc-ADAIN* locus is syntenic in mice, our data (Figure S1) suggest that *linc-ADAIN* is unlikely to be a conservation of the mouse lincRNA (2610016A17Rik) at this syntenic locus. Further 2610016A17Rik is reversed, on the antisense strand, and shares a common promoter region with the nearest protein-coding gene, DMRT2. Thus, it is not appropriate or feasible to generate a 2610016A17Rik-knockout mouse model to study *linc-ADAIN* loss of function *in vivo* (Figure S1C). For this reason, we chose to adapt a transplant model, whereby cultured human adipocytes are implanted with Matrigel into NSG mice[23,24] to generate a functional human fat pad. We implanted day 14 hTERT ASC human adipocytes overexpressing shRNA against *linc-ADAIN* or scramble control into the flank regions of female NSG mice. We extracted these human adipose transplants after 16 weeks *in vivo*. The mice had no significant changes in blood cell counts, weight gain, or organ weight during the protocol (Figures S10C–S10E). Compared to controls, *linc-ADAIN*-KD explants had significantly fewer small adipocytes (<20 μm diameter) and more large adipocytes (>50 μm diameter) (Figures 7A and 7B), and the *linc-ADAIN*-KD explants weighed significantly more than control explants, and the mice with *linc-ADAIN*-KD implants also had increased plasma human adiponectin compared to control mice (Figures S10A and S10B). *linc-ADAIN*-KD explants also had increased infiltration of macrophages, as measured by CD68 and F4/80 (*Adgre1*) by immunohistochemistry (IHC) (Figures 7A, 7C, and 7D) and via qPCR (Figures 7E and 7F). Although not all were statistically significant, compared to control explants, mRNA levels of MCP-1, IL-8, IL-6, and KLF5 tended to be higher in *linc-ADAIN*-KD explants (Figures 7G, 7H, 7I, and 7J). Expression of adipogenic genes, like PPARγ and CEBPα, that was increased by *linc-ADAIN* KD *in vitro* was decreased *in vivo* (Figure 7K). This may be due to increased infiltration of macrophages and their secreted inflammatory factors *in vivo*, which has previously been shown to drive down PPARγ and CEBPα gene expression in adjacent mature adipocytes.[38,39] We found no evidence that KD of *linc-ADAIN* in implants had

Figure 3. *linc-ADAIN* knockdown increases lipid deposition, the adipogenic program, and cytokine secretion

(A–C) Nile red staining of ASC hTERT adipocytes expressing shRNA against *linc-ADAIN* or scramble control (A and B) (scale bar, 20 μm) and triglyceride accumulation at days 14 and 21 post differentiation (C).

(D) qPCR of adipogenic gene expression upon *linc-ADAIN* KD with shRNA vs. scramble control at 14 days (*N* = 3 in duplicate).

(E) Western blot of adipogenic markers in scramble and *linc-ADAIN* shRNA ASC adipocytes at day 14: PPARγ, FASN, ATGL, perilipin 1, FABP4, GLUT4, C/EBPα, and adiponectin.

(F) Luminex protein panel in adipocyte medium at 14 days (*N* = 3 in duplicate).

(G) Fold change of transcripts per million (TPM) of cytokine mRNAs via RNA-seq upon *linc-ADAIN* KD at 14 days (*N* = 1 in triplicate). All data, $^{**}p < 0.01$, $^{***}p < 0.001$ with respect to scramble shR by two-way ANOVA. Data are presented as the mean ± SEM.

A

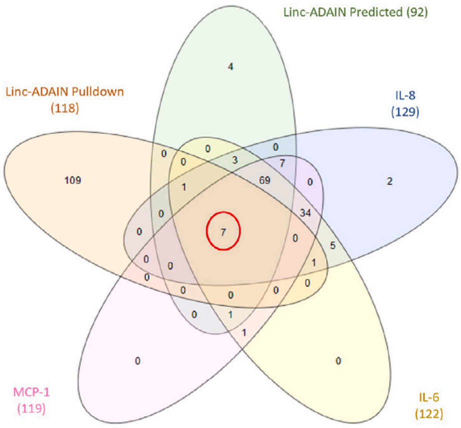

B

Protein	Protein Location	Detected by MS pulldown assay Linc-ADAIN: Antisense (TSC)	Linc-ADAIN (TSC)	Predicted by RBP map Linc-ADAIN: Z-score	P-value	# of binding motifs found in RNA sequence
**HuR**	Nucleus and Cytoplasm	1	13	3.538	0.0002	25
**IGF2BP2**	Cytoplasm	1	21	3.342	0.0004	11
**G3BP2**	Cytoplasm	1	10	3.213	0.0007	5
**FXR2**	Cytoplasm	1	15	2.643	0.0041	3
**MATR3**	Nucleus	1	6	2.203	0.0138	2
**FMR1**	Cytoplasm	1	9	2.194	0.0141	1
**SFPQ**	Nucleus	2	50	2.13	0.0166	5

C

Biological Process (Gene Ontology)				
**GO term**	**Description**	**Count in network**	**Strength**	**False discovery rate**
GO:0045727	Positive regulation of translation	3 of 131	1.81	0.0106
GO:0043488	Regulation of mRNA stability	4 of 183	1.79	0.0012
GO:0017148	Negative regulation of translation	3 of 136	1.79	0.011
GO:0050658	RNA transport	4 of 189	1.77	0.0012
GO:0051028	mRNA transport	3 of 148	1.75	0.0116

D

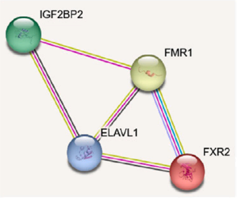

E

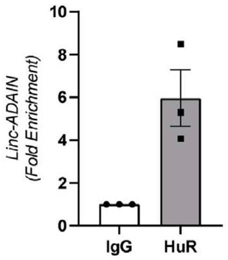

F

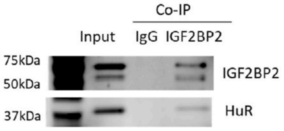

G

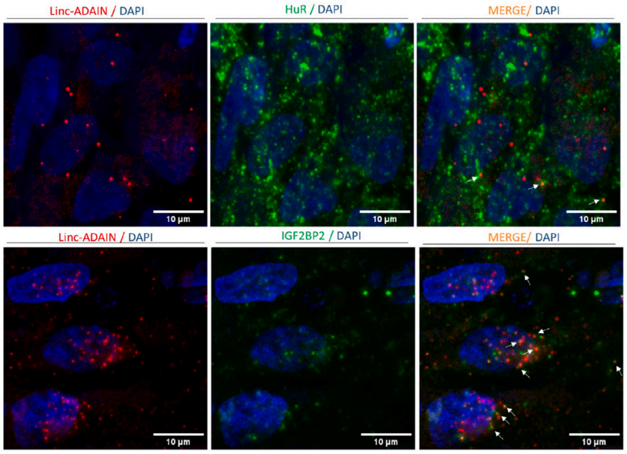

(legend on next page)

any significant effect *in vivo* on systemic glucose and insulin tolerance or on markers of fatty liver (Figures S10F–S10H), although this chow-fed model may not be optimized to study metabolic stress *in vivo*. Overall, these results suggest that *linc-ADAIN* is a human lincRNA that limits adipose tissue expansion and lipid storage while reducing macrophage infiltration of adipose *in vivo*.

DISCUSSION

Adipose tissue can store excess calories through two mechanisms, hypertrophy and hyperplasia. In hypertrophy, the enlargement of existing adipocytes, adipose becomes hypoxic due to limited angiogenesis, as well as inflamed and insulin resistant.[40–42] This dysfunction contributes to CMDs.[43] Here we uncover how *linc-ADAIN*, an adipose lincRNA downregulated in human sWAT during obesity, regulates adipocyte lipid storage and secretion of cytokines. Although the publicly annotated *linc-ADAIN*, known as linc01230, has been shown to have modulated expression in endometrial cancer,[44] renal cell carcinoma,[45] and lung adenocarcinoma,[46] our data suggest that in the context of non-cancer human pathophysiology, *linc-ADAIN* is preferentially expressed in adipose over other tissues and is restricted to adipocytes within human WAT. We found that *linc-ADAIN* interacts and modulates the RBPs HuR and IGF2BP2 in adipocytes to reduce the stability of KLF5 and IL-8 mRNAs. *linc-ADAIN* disrupts the binding of IGF2BP2 to these mRNAs, increasing their decay and reducing protein translation. When *linc-ADAIN* is depleted, KLF5 and IL-8 mRNA binding to IGF2BP2 is enhanced, stabilizing their mRNAs and thus increasing their protein expression and secretion. Increased KLF5 enhances later adipocyte differentiation and lipid storage *in vitro* with higher PPARγ1 and 2 expression compared to when *linc-ADAIN* is present. *In vivo*, adipocyte implants with *linc-ADAIN* depletion were much larger and had evidence for increased macrophage infiltration. *linc-ADAIN* is induced by PPARγ early during adipocyte differentiation, whereas the increases in PPARγ induced by *linc-ADAIN* KD during later adipocyte differentiation are likely mediated indirectly by stabilization of KLF5 and the induction of PPARγ by sustained KLF5 expression. *In vivo*, adipocyte implants with *linc-ADAIN* depletion were much larger and had evidence for increased macrophage infiltration. While the increase in PPARγ coincident with increased adipose inflammation with linc-ADAIN KD may seems counterintuitive, this combination has often been observed in adipose remodeling in both experimental models and clinical obesity.[47,48] Thus, *linc-ADAIN* is an adipose anti-inflammatory lincRNA that can modulate lipid storage and inflammation, two key features of adipose tissue remodeling in obesity.

RBPs play a central role in post-transcriptional gene regulation[49] and fundamental roles in cellular activities such as proliferation and differentiation. There is increasing evidence for the interplay of lncRNAs and RBPs in physiological and pathological contexts such as cancer.[50] Yet, few RBPs have been studied in the context of adipogenesis.[51] HuR (also known as ELAV1) is a ubiquitously expressed member of the Hu family of RNA-binding proteins that selectively binds adenylate uridylate-rich elements (AREs) and thereby stabilizes its RNA targets.[52] HuR regulates the expression of mRNAs and proteins involved in cellular processes such as inflammation and apoptosis.[53,54] Transgenic overexpression of HuR in myeloid cells modulated TNF-α, IL-1β, and TGF-β1 levels in the serum of LPS-challenged mice, and the effect was attributed to enhanced mRNA stability.[53] HuR acts in a tissue-specific manner through cell-specific targets to exert distinctive functions on different adipose depots.[31] Adipose knockout (AKO) of HuR in mice fed a high-fat diet led to increased WAT fat mass, adipocyte hypertrophy, increased circulating total cholesterol, increased triglycerides, insulin resistance, and glucose intolerance.[32] Adipocyte hypertrophy in HuR AKO was attributed to reduced ATGL expression and ATGL mRNA stability, leading to reduced lipolysis.[32] HuR and ATGL expression is reduced in subcutaneous adipose tissue of obese humans.[32] Of specific relevance to the actions of *linc-ADAIN*, AKO of HuR can enhance the adipogenic gene program and an inflammatory program in epidydimal white fat, by regulating the stability of hundreds of adipocyte mRNAs, including Insig1.[31] In the presence of *linc-ADAIN*, we observe increased binding of cytokines IL-8, MCP-1, and IL-6 to HuR compared to when *linc-ADAIN* is depleted. Indeed HuR has been shown to enhance the stabilization of IL-8,[55] IL-6,[56] and MCP-1[57] in other cell types. HuR complexes with IGF2BP family proteins are part of the coding region instability determinant complex, which stabilize mRNAs to protect them from degradation, with c-myc mRNA being a prime example.[21,30]

The RBP IGF2BP2 has been shown to interact with lncRNAs in adipocytes by us and others, regulating adipocyte differentiation and lipogenesis via *linc-ADAL*[16] or lipolysis via *lncRAP*.[58] In genome-wide association studies (GWASs), the IGF2BP2 locus has been repeatedly associated with T2D.[59,60] Similar to HuR, IGF2BP2 functions are tissue specific due to IGF2BP2 binding different RNAs across tissues.[51] Global deletion of IGF2BP2 in mice leads to resistance to high-fat-diet-induced obesity, fatty liver, and glucose tolerance, due to increased protein but not mRNA of UCP1, which promotes increased energy expenditure. IGF2BP2 binds to UCP1 mRNA and inhibits its translation in brown adipose tissue (BAT).[61] In contrast, hepatocyte-specific IGF2BP2 knockout results in diet-induced fatty liver by impairing

Figure 4. *linc-ADAIN* interacts with HuR and IGF2BP2 in human adipocytes

(A and B) Venn diagram overlapping *linc-ADAIN* biotinylated pull-down-MS with ≥5-fold total spectrum count (TSC) with *linc-ADAIN* RNA compared to antisense control and RBPmap-predicted binding proteins for *linc-ADAIN*, *IL-8*, *MCP-1*, and *IL-6* mRNAs (A) and the table of six proteins that overlapped (B).

(C) STRING analysis of the six proteins' gene ontology.

(D) Proteins involved in mRNA stability (IGF2BP2, ELAVL1 [HuR], FMR1, and FXR2).

(E) RNA immunoprecipitation assay (RIP) measuring interaction between HuR and *linc-ADAIN* in ASC hTERT adipocytes.

(F) Western blot of co-immunoprecipitation assay with IGF2BP2 in ASC hTERT adipocytes and HuR.

(G) RNA SCOPE (*linc-ADAIN*) immunofluorescence (IF) of HuR and IGF2BP2 by confocal microscopy in ASC hTERT adipocytes (white arrows point to co-localization [orange] of *linc-ADAIN* and proteins HuR or IGF2BP2; scale bar, 10 μm). Data are presented as the mean ± SEM.

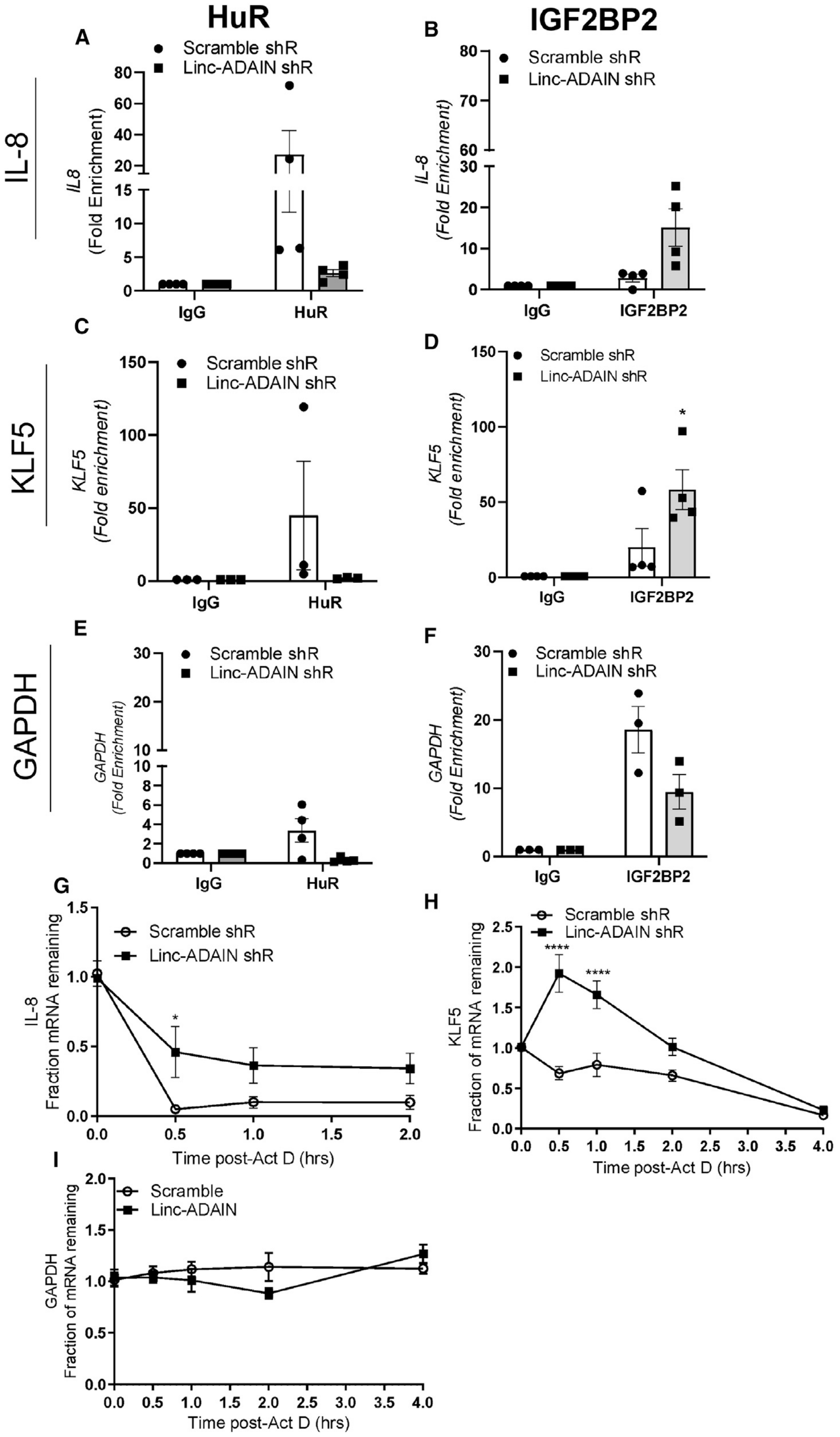

(legend on next page)

fatty acid oxidation.[62] Like *linc-ADAIN*, many lincRNAs are tissue specific in their expression,[63] providing one molecular mechanism for how RBPs have tissue-specific functions, e.g., for HuR.[32,53] Through direct interactions with lincRNAs, HuR and IGF2BP2 can stabilize and increase the translation of certain mRNAs in cancer.[64] For example, *circEIF3H* acts as a scaffold for IGF2BP2 and HuR to regulate mRNA stability of HSPD1, RB8A, and G3BP1 in triple-negative breast cancer.[65] We found HuR bound to IGF2BP2 in human adipocytes, and when *linc-ADAIN* was depleted, this enhanced the binding of HuR and IGF2BP2 to IL-8 and KLF5 mRNA, increasing their mRNA stability and protein levels. IGF2BP2 is known to recruit HuR and MATR3 as stabilizers,[21] and we also found that MATR3 was present in our *linc-ADAIN* pull-down (Figure 4B).

lincRNAs can regulate cytokines in a transcriptional and post-transcriptional manner.[66] *linc-ADAIN* depletion increased IL-8 mRNA stability, mRNA levels, and protein secretion, likely through increased binding of IGF2BP2 to IL-8 mRNAs. IL-8 mRNA stabilization in macrophages is distinct from other cytokines (TNF-α and IL-6) and is ARE independent.[67] IL-8 mRNA is unstable in undifferentiated THP-1 monocytes but becomes constitutively elevated and stabilized in differentiated THP-1 macrophage cells.[67] IL-8 stabilization is cell-type dependent. We found high expression of IL-8 mRNA in pre-adipocytes, which then dropped during adipocyte differentiation coincident with an increase in *linc-ADAIN*. IL-8 is a chemokine for neutrophils, and in early stages of obesity, neutrophils infiltrate adipose tissue, producing chemokines that promote macrophage infiltration.[68] Indeed, with *linc-ADAIN* depletion we observed increased macrophage infiltration in 16-week-old adipose explants, which may be mediated by increased IL-8.

KLF5 is an early transcription factor for adipocyte differentiation and adipogenesis[20] and is needed to activate the PPARγ2 promoter. KLF5 is induced early (within 1 h) during adipocyte differentiation and gradually declines thereafter.[20] KLF5$^{+/-}$ mice have delayed adipose tissue development, have reduced lipid droplets, and are resistant to high-fat-diet-induced obesity.[20] *linc-ADAIN* depletion during adipocyte differentiation elevated KLF5 in pre-adipocytes and resulted in sustained KLF5 levels and increased PPARγ1 and 2 expression, as well as levels of adipogenic genes. Like IL-8, we observed enhanced stability of KLF5 mRNA with *linc-ADAIN* depletion, likely due to its increased binding to IGF2BP2 protein. Notably, both KLF5 and IL-8 mRNA levels are high in pre-adipocytes and decline during differentiation, coincident with increased *linc-ADAIN* expression. When *linc-ADAIN* is depleted, both KLF5 and IL-8 remain high throughout differentiation, suggesting that *linc-ADAIN* is an important regulator of their expression and functions during physiological adipocyte differentiation, adipogenesis, and lipid storage in humans. Furthermore, consistent with KLF5 and IL-8 being potential mediators of linc-ADAIN effects, we found that KD of IL-8 and KLF5 produced the phenotypes opposite that observed with linc-ADAIN KD. Our findings suggest a potential negative feedback regulation by *linc-ADAIN* of KLF5 under normal physiological circumstances. With reduction of *linc-ADAIN* expression experimentally or in the context of obesity, this regulation may be lost, permitting adipose remodeling and expansion. To our knowledge, *linc-ADAIN* is the only lincRNA to be shown to regulate KLF5 or IL-8 expression and functions within adipose tissue.

KLF5 expression can promote some cancers and tumor angiogenesis.[69] For example, KLF5 promoted angiogenesis in bladder cancer by directly increasing vascular endothelial growth factor A (VEGFA) transcription.[70] KD of lncRNA MCM3AP-AS1 increases miR-211, which targets KLF5 and inhibits its expression, affecting angiogenesis in glioblastoma.[71] Interestingly, IL-8 is also known as a pro-angiogenic factor, particularly in cancer.[72] In our pathway analyses of adipocyte RNA-seq data from *linc-ADAIN* KD vs. scramble control, angiogenesis was one of the top biological processes identified. Angiogenesis is necessary for normal adipose tissue development to provide nutrients and oxygen. In obesity, angiogenesis tends to precede hyperplastic expansion, but follows hypertrophic expansion, leading to hypoxia in hypertrophic expansion.[73] The mechanisms by which adipose tissue growth is coordinated with capillary networks are still not fully understood and may underlie aspects of adipose tissue dysfunction in obesity and metabolic disease.[73] *linc-ADAIN* increases during adipocyte differentiation and regulates IL-8 and KLF5 expression and thus may contribute to adipose tissue remodeling through adipocyte expansion, lipid storage, angiogenesis, and immune cell recruitment during normal adipose tissue development and physiology.

Most lincRNAs are not conserved across species; thus, creating knockout or overexpression mouse models is challenging. *linc-ADAIN* has a syntenic locus in mouse, yet *linc-ADAIN* is probably not a conservation of the mouse 2610016A17Rik lincRNA at this locus. In addition, mouse 2610016A17Rik is on the antisense strand and shares a promoter region with DMRT2. Thus, deleting 2610016A17Rik would likely affect DMRT2 expression in the mouse and confound studies of 2610016A17Rik. Therefore, we adapted an implant model to generate a functional human adipose fat pad in mice.[23,24] Human ASC hTERT adipocytes with a lentiviral expression of an shRNA targeting *linc-ADAIN* or a scramble control after 14 days of differentiation *in vitro* were implanted into the flanks of NSG mice. We hypothesized that *linc-ADAIN* KD adipocytes would have increased differentiation and lipid storage over time *in vivo* and that increased adipocyte cytokine secretion from *linc-ADAIN* KD would lead to increased macrophage infiltration *in vivo*. By 16 weeks after implantation, *linc-ADAIN*-KD adipocytes produced larger adipose depots and had increased macrophage infiltration compared to scramble shRNA

Figure 5. Knockdown of *linc-ADAIN* stabilizes *IL-8* and *KLF5* mRNA, likely through IGF2BP2

(A–F) RNA immunoprecipitation (RIP) assays measure the interaction of *IL-8*, *KLF5*, and *GAPDH* with HuR (A, C, E) ($N = 3$) and IGF2PBP2 (B, D, F) ($N = 3$–4), upon *linc-ADAIN* shRNA knockdown compared to scramble shRNA in day 14 ASC hTERT adipocytes.

(G–I) Scramble and *linc-ADAIN* shRNA-expressing ASC adipocytes were treated with actinomycin-D to halt transcription and fraction of mRNA measured at 0.5, 1, 2, and 4 h post treatment of *IL-8* (normalized to GAPDH) (G), *KLF5* (normalized to GAPDH) (H), and *GAPDH* (I) ($N = 3$). *$p < 0.05$, **$p < 0.01$ ****$p < 0.0001$ with respect to scramble shRNA by two-way ANOVA. Data are presented as the mean ± SEM.

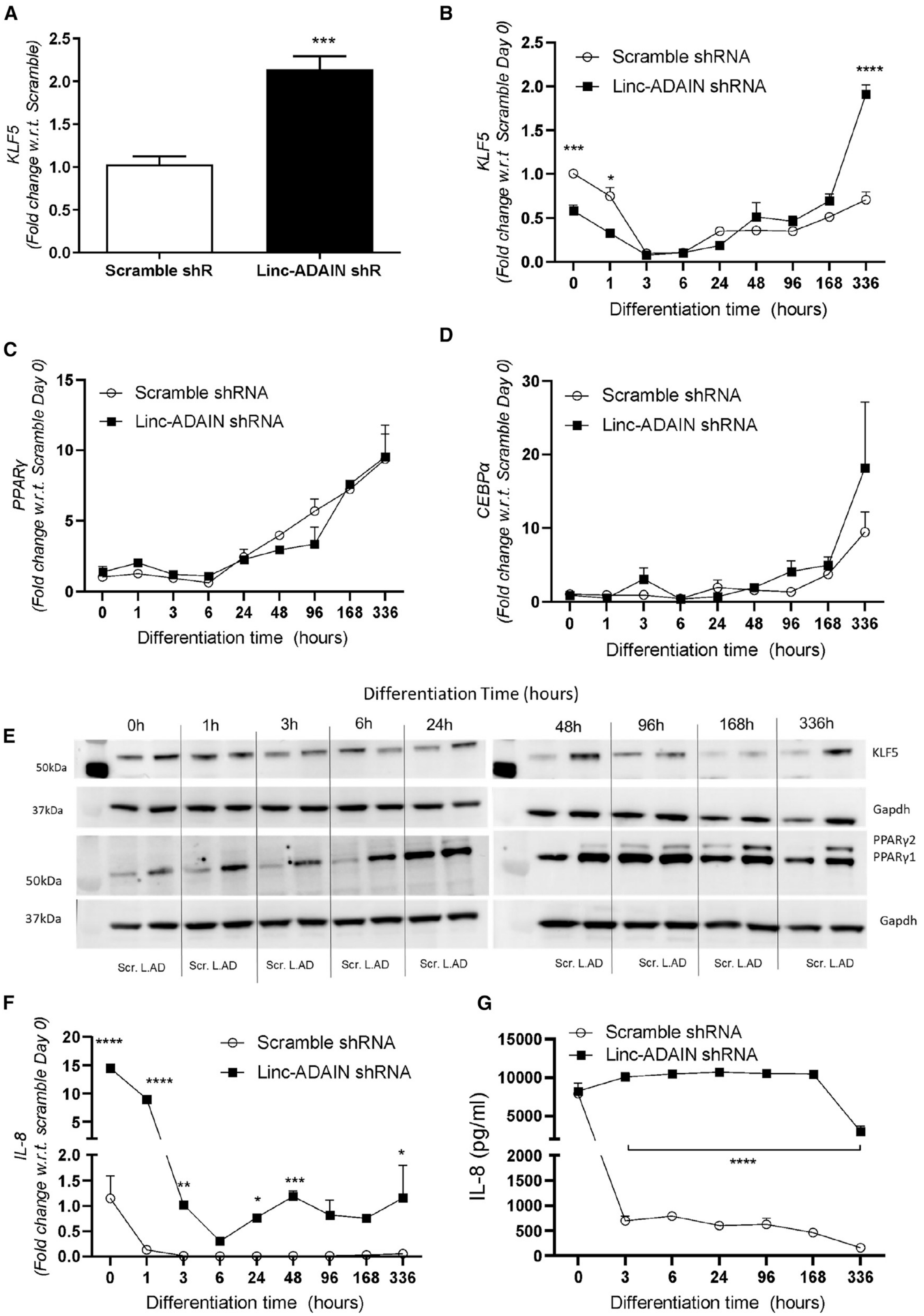

(legend on next page)

adipocytes. These results mirror many hallmark features of adipose tissue remodeling in obesity and provide proof of principle that *linc-ADAIN* regulates adipogenesis, lipid storage, and tissue inflammation *in vivo*.

Limitations of the study

In our work, the role of linc-ADAIN in adipogenesis has been characterized in much more detail than its role in adipose tissue inflammation. To understand fully its role in modulating adipose tissue inflammation will require more work, including a closer examination *in vitro* and *in vivo* of the effects of adipose linc-ADAIN on recruitment of neutrophils as well as of macrophages via linc-ADAIN-regulated chemokines and cytokines, especially IL-8. Our *in vivo* adipocyte transplant model is innovative but challenging and has limitations for assessing whole-body metabolic effects of this non-conserved lincRNA. Future *in vivo* studies using high-fat-diet feeding can examine the effects of adipose *linc-ADAIN* expression on systemic measures of glucose tolerance, insulin sensitivity, and adipocyte-to-macrophage crosstalk under pathophysiological stress. We could not address directly why *linc-ADAIN* is downregulated in obesity. We hypothesize this may be due to reduced PPARγ activity caused by inflammatory macrophage-secreted factors in obese adipose.[39] Our data showing PPARγ ChIP-seq peaks upstream of *linc-ADAIN* and PPARγ agonist, rosiglitazone, induction of *linc-ADAIN* suggest that PPARγ is a transcriptional activator of *linc-ADAIN*. Previously, the publicly annotated *linc-ADAIN*, known as linc01230, had been shown to be transcriptionally activated by PPARγ in endothelial cells.[19] Overexpression of *linc-ADAIN* in ASC-hTERTs using a tetracycline-inducible lentivirus (pSILKNeo) system did not significantly change lipid storage or inflammatory gene expression. However, viral vector overexpression of lincRNAs is associated with several technical challenges, including introduction of additional sequence elements that may have non-specific effects and failure of ectopic expression to reproduce physiological subcellular localization. For lincRNAs where endogenous expression is physiologically "protective," overexpression systems fail to produce the phenotype opposite to the loss-of-function models.[74,75] Furthermore, we emphasize that our data show significant, consistent and reproducible effects of independent experimental KD approaches using shRNA and ASO in cultured adipocytes *in vitro* as well as in our adipocyte transplant model *in vivo*. In humans, *linc-ADAIN* is downregulated in obese human adipose tissue and is suppressed by acute inflammation during endotoxemia, and its expression is negatively correlated with total body fat percentage.[17] Larger human population studies are required to determine if *linc-ADAIN* is a causal gene in CMD traits. Future work can address if induction of *linc-ADAIN* (e.g., through RNA activation) in obese adipose *in vivo* can provide anti-inflammatory and adipose remodeling effects that may attenuate the cardiometabolic complications of obesity. Because cytoplasmic lincRNAs can act as competing endogenous RNAs for microRNA (miRNA), we performed *in silico* analyses using DIANA LncBase[76] and identified predicted linc-ADAIN interactions with hsa-let-7d-5p, hsa-miR-34a-5p, and hsa-miR-423-5p. Although Ago2 was not identified in our protein-binding analyses, future studies of these predicted miRNA interactions may be warranted.

In summary, we provide a functional study of human adipose *linc-ADAIN* and its roles in adipose remodeling. We demonstrate that *linc-ADAIN* is an anti-inflammatory adipose lincRNA that limits adipose expansion and lipid storage and is downregulated in obese white adipose tissue. *linc-ADAIN* fine-tunes adipogenesis and lipid storage via its regulation of IL-8 and KLF5 mRNA stability and expression, two genes of known importance in adipose tissue remodeling and adipogenesis. *linc-ADAIN* inhibits the interaction between IL-8 and KLF5 mRNAs and IGF2BP2, an RBP that stabilizes mRNA as part of the coding region instability determinant complex. When adipocyte *linc-ADAIN* is depleted, IL-8 and KLF5 mRNAs remain stable, and adipocytes have increased mRNA and protein expression throughout adipogenesis, leading to increased expression of IL-8, other chemokines, PPARγ, and the adipogenic program. This results in hypertrophied and inflamed adipocytes. *In vivo*, *linc-ADAIN* KD in adipocytes led to adipocyte hypertrophy and larger fat pads as well as increased macrophage infiltration of fat pads implanted into NSG mice—key features of obesity-induced adipose tissue remodeling in obesity and CMD.

STAR★METHODS

Detailed methods are provided in the online version of this paper and include the following:

- KEY RESOURCES TABLE
- RESOURCE AVAILABILITY
  - Lead contact
  - Materials availability
  - Data and code availability
- METHOD DETAILS
  - Experimental model and study participant details
  - Mouse studies
  - Cellular fractionation
  - 3' RACE (rapid amplification of cDNA ends)
  - *In vitro* transcription/translation assay
  - RNA pulldown assay and mass spectrometry
  - RNA-binding proteins (RBP) map and STRING analysis
  - RNA immunoprecipitation (RIP)
  - RNA isolation and quantitative real-time PCR analysis
  - RNA stability assays
  - Immunoblot analysis
  - Human adipocyte implantation into mice
  - Immunohistochemistry staining (IHC)

Figure 6. KLF5 and IL-8 have sustained upregulation in *linc-ADAIN* shRNA KD during differentiation

(A and B) Gene expression of *KLF5* at day 14 of adipocyte differentiation (A) ($N = 3$ in duplicate); ***$p < 0.001$ with respect to scramble, and during adipocyte differentiation (B).

(C and D) *PPARγ* and *CEBPα* gene expression during adipocyte differentiation.

(E) Protein expression of KLF5, PPARγ, and CEBPα in ASC hTERT adipocytes expressing scramble or *linc-ADAIN* shRNA.

(F and G) *IL-8* gene expression during adipocyte differentiation (F) and IL-8 secretion during adipocyte differentiation ($N = 3$) (G); *$p < 0.05$, **$p < 0.01$, ****$p < 0.0001$ with respect to scramble shRNA control by two-way ANOVA. Data are presented as the mean ± SEM.

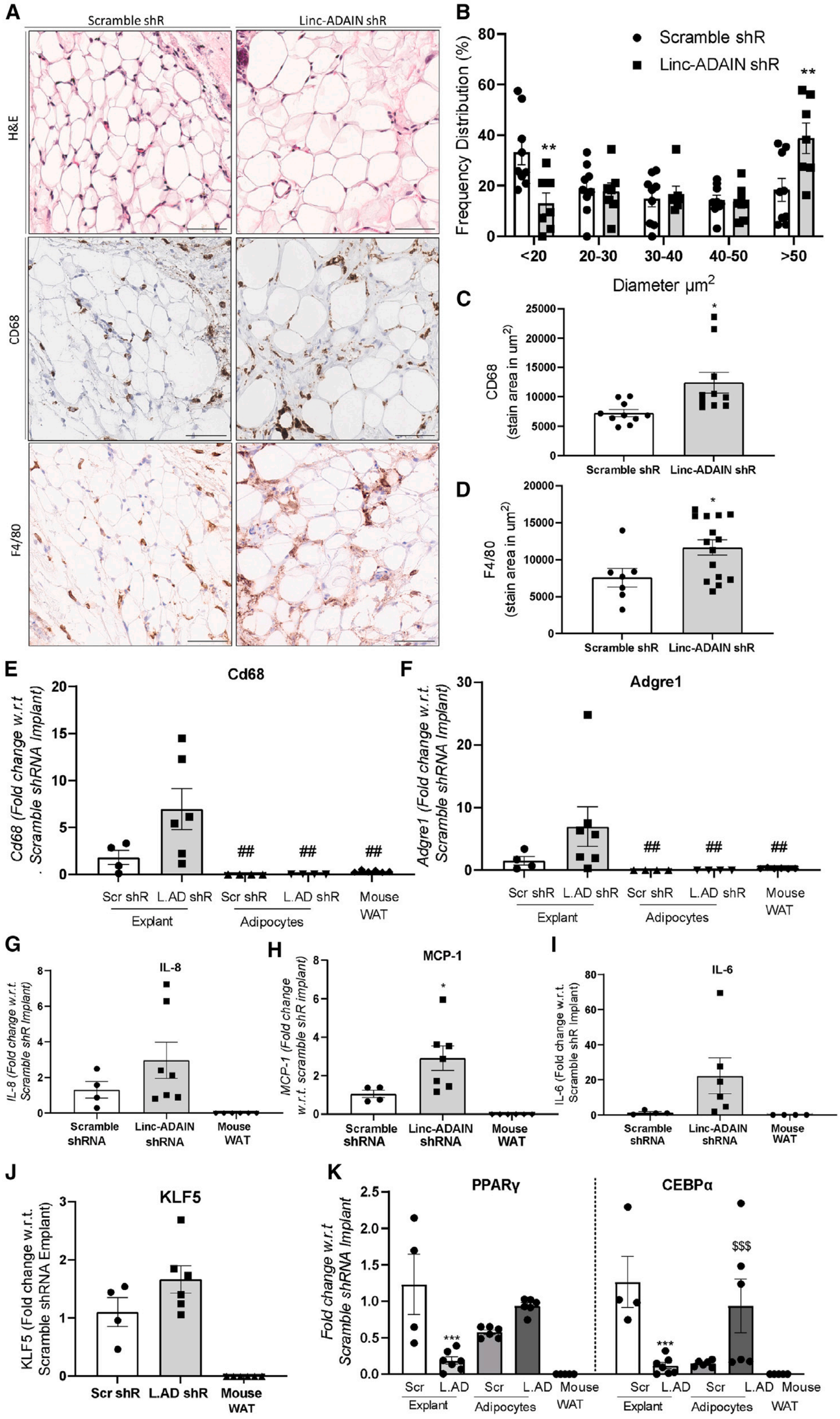

(legend on next page)

- ○ Nile red staining
- ○ RNA scope immunofluorescence (IF) assay
- ○ Ingenuity pathway (IPA) and database for annotation visualization and Integrate discovery (DAVID) gene ontology analysis
- QUANTIFICATION AND STATISTICAL ANALYSIS

SUPPLEMENTAL INFORMATION

Supplemental information can be found online at https://doi.org/10.1016/j.celrep.2024.114240.

ACKNOWLEDGMENTS

The authors would like to acknowledge and thank Dr. Javier Solivan-Rivera for training on the adipocyte implantation model. Confocal images were collected in the Confocal and Specialized Microscopy Shared Resource of the Herbert Irving Comprehensive Cancer Center at Columbia University. IHC was carried out using the Histology Service of the Molecular Pathology Shared Resource at the Columbia University Irving Medical Center. Flow cytometry experiments described in this article were performed in the Columbia Stem Cell Initiative Flow Cytometry core facility at Columbia University Irving Medical Center. RNA sequencing was performed at the J.P. Sulzberger Columbia Genome Center. M.E.O. was supported for this work by an American Heart Association Postdoctoral Fellowship (AHA 20POST35050045). M.P.R. was supported by National Institutes of Health grants R01HL132561, R01HL113147, R01HL150359, and R01HL166916.

AUTHOR CONTRIBUTIONS

Conceptualization, M.E.O. and M.P.R.; methodology, M.E.O., M.P.R., S.C., S.K.F., and Y.Z.; investigation, M.E.O., S.H., J.C., E.C., L.Z., W.L., C.X., E.K., and C.V.M.; formal analysis, M.E.O., C.X., E.K., C.W., and I.I.-L.; resources, M.P.R., R.C.B., S.K.F., and S.C.; data curation, C.X. and E.K.; visualization, M.E.O., S.H., C.X., and E.K.; writing – original draft, M.E.O. and M.P.R.; writing – review & editing, M.E.O., M.P.R., and L.R.; funding acquisition, M.E.O., M.P.R., and L.R.; supervision, M.P.R., S.C., S.K.F., Y.Z., and R.C.B.

DECLARATION OF INTERESTS

The authors declare no competing interests.

Received: July 7, 2023
Revised: March 1, 2024
Accepted: May 1, 2024
Published: May 14, 2024

REFERENCES

1. Mottillo, S., Filion, K.B., Genest, J., Joseph, L., Pilote, L., Poirier, P., Rinfret, S., Schiffrin, E.L., and Eisenberg, M.J. (2010). The metabolic syndrome and cardiovascular risk a systematic review and meta-analysis. J. Am. Coll. Cardiol. *56*, 1113–1132.
2. Valenzuela, P.L., Carrera-Bastos, P., Castillo-García, A., Lieberman, D.E., Santos-Lozano, A., and Lucia, A. (2023). Obesity and the risk of cardiometabolic diseases. Nat Rev Cardiol *20*, 475–494.
3. Xu, H., Barnes, G.T., Yang, Q., Tan, G., Yang, D., Chou, C.J., Sole, J., Nichols, A., Ross, J.S., Tartaglia, L.A., and Chen, H. (2003). Chronic inflammation in fat plays a crucial role in the development of obesity-related insulin resistance. J. Clin. Invest. *112*, 1821–1830.
4. Ye, R.Z., Richard, G., Gévry, N., Tchernof, A., and Carpentier, A.C. (2022). Fat Cell Size: Measurement Methods, Pathophysiological Origins, and Relationships With Metabolic Dysregulations. Endocr. Rev. *43*, 35–60.
5. World Health Organization (2018). Obesity and Overweight Key Facts.
6. Shungin, D., Winkler, T.W., Croteau-Chonka, D.C., Ferreira, T., Locke, A.E., Mägi, R., Strawbridge, R.J., Pers, T.H., Fischer, K., Justice, A.E., et al. (2015). New genetic loci link adipose and insulin biology to body fat distribution. Nature *518*, 187–196.
7. Derrien, T., Johnson, R., Bussotti, G., Tanzer, A., Djebali, S., Tilgner, H., Guernec, G., Martin, D., Merkel, A., Knowles, D.G., et al. (2012). The GENCODE v7 catalog of human long noncoding RNAs: analysis of their gene structure, evolution, and expression. Genome Res. *22*, 1775–1789.
8. Hangauer, M.J., Vaughn, I.W., and McManus, M.T. (2013). Pervasive transcription of the human genome produces thousands of previously unidentified long intergenic noncoding RNAs. PLoS Genet. *9*, e1003569.
9. Ulitsky, I., and Bartel, D.P. (2013). lincRNAs: genomics, evolution, and mechanisms. Cell *154*, 26–46.
10. Corral, A., Alcala, M., Carmen Duran-Ruiz, M., Arroba, A.I., Ponce-Gonzalez, J.G., Todorčević, M., Serra, D., Calderon-Dominguez, M., and Herrero, L. (2022). Role of long non-coding RNAs in adipose tissue metabolism and associated pathologies. Biochem. Pharmacol. *206*, 115305.
11. Mattick, J.S., Amaral, P.P., Carninci, P., Carpenter, S., Chang, H.Y., Chen, L.L., Chen, R., Dean, C., Dinger, M.E., Fitzgerald, K.A., et al. (2023). Long non-coding RNAs: definitions, functions, challenges and recommendations. Nat Rev Mol Cell Biol *24*, 430–447.
12. Liu, S.J., Dang, H.X., Lim, D.A., Feng, F.Y., and Maher, C.A. (2021). Long noncoding RNAs in cancer metastasis. Nat. Rev. Cancer *21*, 446–460.
13. Divoux, A., Karastergiou, K., Xie, H., Guo, W., Perera, R.J., Fried, S.K., and Smith, S.R. (2014). Identification of a novel lncRNA in gluteal adipose tissue and evidence for its positive effect on preadipocyte differentiation. Obesity *22*, 1781–1785.
14. Kuo, F.C., Huang, Y.C., Yen, M.R., Lee, C.H., Hsu, K.F., Yang, H.Y., Wu, L.W., Lu, C.H., Hsu, Y.J., and Chen, P.Y. (2022). Aberrant overexpression of HOTAIR inhibits abdominal adipogenesis through remodelling of genome-wide DNA methylation and transcription. Mol. Metabol. *60*, 101473.
15. Tran, K.V., Brown, E.L., DeSouza, T., Jespersen, N.Z., Nandrup-Bus, C., Yang, Q., Yang, Z., Desai, A., Min, S.Y., Rojas-Rodriguez, R., et al. (2020). Human thermogenic adipocyte regulation by the long noncoding RNA LINC00473. Nat. Metab. *2*, 397–412.
16. Zhang, X., Xue, C., Lin, J., Ferguson, J.F., Weiner, A., Liu, W., Han, Y., Hinkle, C., Li, W., Jiang, H., et al. (2018). Interrogation of nonconserved human adipose lincRNAs identifies a regulatory role of linc-ADAL in adipocyte metabolism. Sci. Transl. Med. *10*, eaar5987.
17. Liu, Y., Ferguson, J.F., Xue, C., Ballantyne, R.L., Silverman, I.M., Gosai, S.J., Serfecz, J., Morley, M.P., Gregory, B.D., Li, M., and Reilly, M.P. (2014). Tissue-specific RNA-Seq in human evoked inflammation identifies blood and adipose LincRNA signatures of cardiometabolic diseases. Arterioscler. Thromb. Vasc. Biol. *34*, 902–912.

Figure 7. Adipocytes with reduced *linc-ADAIN* show increased size and lipid storage and macrophage infiltration *in vivo*

(A and B) ASC adipocytes with scramble or *linc-ADAIN* shRNA were implanted into the flanks of NSG mice and removed 16 weeks later. H&E staining of scramble and *linc-ADAIN*-KD implants after 16 weeks (scale bar, 60 μm) (A) and size distribution of adipocytes, quantified with Adiposoft in a 300 × 300 μm section (B).
(C and D) CD68 (C) and F4/80 (D) IHC staining area quantified.
(E–K) Gene expression of mouse *Cd68* (E) and *Adgre1* (F) and human expression of *IL-8* (G), *MCP-1* (H), *IL-6* (I), *KLF5* (J), and *PPARγ* and *CEBPα* (K) in the scramble and *linc-ADAIN* shRNA-expressing explants after 16 weeks or the ASC hTERT adipocytes prior to implantation (N = 4–7); *$p < 0.05$, **$p < 0.01$, ***$p < 0.001$ with relation to scramble shRNA explant; $^{\#\#}p < 0.01$ with relation to *linc-ADAIN* shRNA explant; $^{\$\$\$}p < 0.01$ with reslation to scramble shRNA adipocytes by two-way ANOVA. Data are presented as the mean ± SEM.

18. Uszczynska-Ratajczak, B., Lagarde, J., Frankish, A., Guigó, R., and Johnson, R. (2018). Towards a complete map of the human long non-coding RNA transcriptome. Nat. Rev. Genet. *19*, 535–548.

19. Liu, L., Wang, Z., Jia, J., Shi, Y., Lian, T., and Han, X. (2018). Linc01230, transcriptionally regulated by PPARgamma, is identified as a novel modifier in endothelial function. Biochem. Biophys. Res. Commun. *507*, 369–376.

20. Oishi, Y., Manabe, I., Tobe, K., Tsushima, K., Shindo, T., Fujiu, K., Nishimura, G., Maemura, K., Yamauchi, T., Kubota, N., et al. (2005). Kruppel-like transcription factor KLF5 is a key regulator of adipocyte differentiation. Cell Metabol. *1*, 27–39.

21. Huang, H., Weng, H., Sun, W., Qin, X., Shi, H., Wu, H., Zhao, B.S., Mesquita, A., Liu, C., Yuan, C.L., et al. (2018). Recognition of RNA N(6)-methyladenosine by IGF2BP proteins enhances mRNA stability and translation. Nat. Cell Biol. *20*, 285–295.

22. Fan, X.C., and Steitz, J.A. (1998). Overexpression of HuR, a nuclear-cytoplasmic shuttling protein, increases the in vivo stability of ARE-containing mRNAs. EMBO J. *17*, 3448–3460.

23. Rojas-Rodriguez, R., Lujan-Hernandez, J., Min, S.Y., DeSouza, T., Teebagy, P., Desai, A., Tessier, H., Slamin, R., Siegel-Reamer, L., Berg, C., et al. (2019). Generation of Functional Human Adipose Tissue in Mice from Primed Progenitor Cells. Tissue Eng. *25*, 842–854.

24. Min, S.Y., Kady, J., Nam, M., Rojas-Rodriguez, R., Berkenwald, A., Kim, J.H., Noh, H.L., Kim, J.K., Cooper, M.P., Fitzgibbons, T., et al. (2016). Human 'brite/beige' adipocytes develop from capillary networks, and their implantation improves metabolic homeostasis in mice. Nat. Med. *22*, 312–318.

25. Arner, E., Daub, C.O., Vitting-Seerup, K., Andersson, R., Lilje, B., Drabløs, F., Lennartsson, A., Rönnerblad, M., Hrydziuszko, O., Vitezic, M., et al. (2015). Transcribed enhancers lead waves of coordinated transcription in transitioning mammalian cells. Science *347*, 1010–1014.

26. Mudge, J.M., Jungreis, I., Hunt, T., Gonzalez, J.M., Wright, J.C., Kay, M., Davidson, C., Fitzgerald, S., Seal, R., Tweedie, S., et al. (2019). Discovery of high-confidence human protein-coding genes and exons by whole-genome PhyloCSF helps elucidate 118 GWAS loci. Genome Res. *29*, 2073–2087.

27. Emont, M.P., Jacobs, C., Essene, A.L., Pant, D., Tenen, D., Colleluori, G., Di Vincenzo, A., Jørgensen, A.M., Dashti, H., Stefek, A., et al. (2022). A single-cell atlas of human and mouse white adipose tissue. Nature *603*, 926–933.

28. Deshmukh, A.S., Peijs, L., Beaudry, J.L., Jespersen, N.Z., Nielsen, C.H., Ma, T., Brunner, A.D., Larsen, T.J., Bayarri-Olmos, R., Prabhakar, B.S., et al. (2019). Proteomics-Based Comparative Mapping of the Secretomes of Human Brown and White Adipocytes Reveals EPDR1 as a Novel Batokine. Cell Metabol. *30*, 963–975.e7.

29. Paz, I., Kosti, I., Ares, M., Jr., Cline, M., and Mandel-Gutfreund, Y. (2014). RBPmap: a web server for mapping binding sites of RNA-binding proteins. Nucleic Acids Res. *42*, W361–W367.

30. Weidensdorfer, D., Stöhr, N., Baude, A., Lederer, M., Köhn, M., Schierhorn, A., Buchmeier, S., Wahle, E., and Hüttelmaier, S. (2009). Control of c-myc mRNA stability by IGF2BP1-associated cytoplasmic RNPs. RNA *15*, 104–115.

31. Siang, D.T.C., Lim, Y.C., Kyaw, A.M.M., Win, K.N., Chia, S.Y., Degirmenci, U., Hu, X., Tan, B.C., Walet, A.C.E., Sun, L., and Xu, D. (2020). The RNA-binding protein HuR is a negative regulator in adipogenesis. Nat. Commun. *11*, 213.

32. Li, J., Gong, L., Liu, S., Zhang, Y., Zhang, C., Tian, M., Lu, H., Bu, P., Yang, J., Ouyang, C., et al. (2019). Adipose HuR protects against diet-induced obesity and insulin resistance. Nat. Commun. *10*, 2375.

33. Bell, J.L., Wächter, K., Mühleck, B., Pazaitis, N., Köhn, M., Lederer, M., and Hüttelmaier, S. (2013). Insulin-like growth factor 2 mRNA-binding proteins (IGF2BPs): post-transcriptional drivers of cancer progression? Cell. Mol. Life Sci. *70*, 2657–2675.

34. Cao, J., Mu, Q., and Huang, H. (2018). The Roles of Insulin-Like Growth Factor 2 mRNA-Binding Protein 2 in Cancer and Cancer Stem Cells. Stem Cell. Int. *2018*, 4217259.

35. Hu, X., Peng, W.X., Zhou, H., Jiang, J., Zhou, X., Huang, D., Mo, Y.Y., and Yang, L. (2020). IGF2BP2 regulates DANCR by serving as an N6-methyladenosine reader. Cell Death Differ. *27*, 1782–1794.

36. Bruun, J.M., Lihn, A.S., Madan, A.K., Pedersen, S.B., Schiøtt, K.M., Fain, J.N., and Richelsen, B. (2004). Higher production of IL-8 in visceral vs. subcutaneous adipose tissue. Implication of nonadipose cells in adipose tissue. Am. J. Physiol. Endocrinol. Metab. *286*, E8–E13.

37. Cervantes-Camacho, C., Beltrán-Langarica, A., Ochoa-Uribe, A.K., Marsch-Moreno, M., Ayala-Sumuano, J.T., Velez-delValle, C., and Kuri-Harcuch, W. (2015). The transient expression of Klf4 and Klf5 during adipogenesis depends on GSK3beta activity. Adipocyte *4*, 248–255.

38. Lacasa, D., Taleb, S., Keophiphath, M., Miranville, A., and Clement, K. (2007). Macrophage-secreted factors impair human adipogenesis: involvement of proinflammatory state in preadipocytes. Endocrinology *148*, 868–877.

39. Ma, H., Li, Y.N., Song, L., Liu, R., Li, X., Shang, Q., Wang, Y., Shao, C., and Shi, Y. (2020). Macrophages inhibit adipogenic differentiation of adipose tissue derived mesenchymal stem/stromal cells by producing pro-inflammatory cytokines. Cell Biosci. *10*, 88.

40. Kim, S.M., Lun, M., Wang, M., Senyo, S.E., Guillermier, C., Patwari, P., and Steinhauser, M.L. (2014). Loss of white adipose hyperplastic potential is associated with enhanced susceptibility to insulin resistance. Cell Metabol. *20*, 1049–1058.

41. Cifarelli, V., Beeman, S.C., Smith, G.I., Yoshino, J., Morozov, D., Beals, J.W., Kayser, B.D., Watrous, J.D., Jain, M., Patterson, B.W., and Klein, S. (2020). Decreased adipose tissue oxygenation associates with insulin resistance in individuals with obesity. J. Clin. Invest. *130*, 6688–6699.

42. Lempesis, I.G., van Meijel, R.L.J., Manolopoulos, K.N., and Goossens, G.H. (2020). Oxygenation of adipose tissue: A human perspective. Acta Physiol. *228*, e13298.

43. Longo, M., Zatterale, F., Naderi, J., Parrillo, L., Formisano, P., Raciti, G.A., Beguinot, F., and Miele, C. (2019). Adipose Tissue Dysfunction as Determinant of Obesity-Associated Metabolic Complications. Int. J. Mol. Sci. *20*, 2358.

44. Bienkiewicz, J., Romanowicz, H., Szymanska, B., Domanska-Senderowska, D., Wilczynski, M., Stepowicz, A., Malinowski, A., Smolarz, B., et al. (2022). Analysis of lncRNA sequences: FAM3D-AS1, LINC01230, LINC01315 and LINC01468 in endometrial cancer. BMC Cancer *22*, 343.

45. Bohosova, J., Kasik, M., Kubickova, A., Trachtova, K., Stanik, M., Poprach, A., and Slaby, O. (2022). LncRNA PVT1 is increased in renal cell carcinoma and affects viability and migration in vitro. J. Clin. Lab. Anal. *36*, e24442.

46. Li, Q., Wang, T., Zhu, J., Zhang, A., Wu, A., Zhou, Y., and Shi, J. (2023). A cuproptosis-related lncRNAs risk model to predict prognosis and guide immunotherapy for lung adenocarcinoma. Ann. Transl. Med. *11*, 198.

47. Vidal-Puig, A.J., Considine, R.V., Jimenez-Liñan, M., Werman, A., Pories, W.J., Caro, J.F., and Flier, J.S. (1997). Peroxisome proliferator-activated receptor gene expression in human tissues. Effects of obesity, weight loss, and regulation by insulin and glucocorticoids. J. Clin. Invest. *99*, 2416–2422.

48. Hotamisligil, G.S. (2006). Inflammation and metabolic disorders. Nature *444*, 860–867.

49. Glisovic, T., Bachorik, J.L., Yong, J., and Dreyfuss, G. (2008). RNA-binding proteins and post-transcriptional gene regulation. FEBS Lett. *582*, 1977–1986.

50. Yao, Z.T., Yang, Y.M., Sun, M.M., He, Y., Liao, L., Chen, K.S., and Li, B. (2022). New insights into the interplay between long non-coding RNAs and RNA-binding proteins in cancer. Cancer Commun. *42*, 117–140.

51. Zhang, P., Wu, W., Ma, C., Du, C., Huang, Y., Xu, H., Li, C., Cheng, X., Hao, R., and Xu, Y. (2022). RNA-Binding Proteins in the Regulation of Adipogenesis and Adipose Function. Cells *11*, 2357.

52. Srikantan, S., and Gorospe, M. (2012). HuR function in disease. Front. Biosci. *17*, 189–205.

53. Katsanou, V., Papadaki, O., Milatos, S., Blackshear, P.J., Anderson, P., Kollias, G., and Kontoyiannis, D.L. (2005). HuR as a negative posttranscriptional modulator in inflammation. Mol. Cell *19*, 777–789.

54. Abdelmohsen, K., Lal, A., Kim, H.H., and Gorospe, M. (2007). Posttranscriptional orchestration of an anti-apoptotic program by HuR. Cell Cycle *6*, 1288–1292.

55. Palanisamy, V., Park, N.J., Wang, J., and Wong, D.T. (2008). AUF1 and HuR proteins stabilize interleukin-8 mRNA in human saliva. J. Dent. Res. *87*, 772–776.

56. Ouhara, K., Munenaga, S., Kajiya, M., Takeda, K., Matsuda, S., Sato, Y., Hamamoto, Y., Iwata, T., Yamasaki, S., Akutagawa, K., et al. (2018). The induced RNA-binding protein, HuR, targets 3'-UTR region of IL-6 mRNA and enhances its stabilization in periodontitis. Clin. Exp. Immunol. *192*, 325–336.

57. Fan, J., Ishmael, F.T., Fang, X., Myers, A., Cheadle, C., Huang, S.K., Atasoy, U., Gorospe, M., and Stellato, C. (2011). Chemokine transcripts as targets of the RNA-binding protein HuR in human airway epithelium. J. Immunol. *186*, 2482–2494.

58. Alvarez-Dominguez, J.R., Winther, S., Hansen, J.B., Lodish, H.F., and Knoll, M. (2022). An adipose lncRAP2-Igf2bp2 complex enhances adipogenesis and energy expenditure by stabilizing target mRNAs. iScience *25*, 103680.

59. Phani, N.M., Adhikari, P., Nagri, S.K., D'Souza, S.C., Satyamoorthy, K., and Rai, P.S. (2016). Replication and Relevance of Multiple Susceptibility Loci Discovered from Genome Wide Association Studies for Type 2 Diabetes in an Indian Population. PLoS One *11*, e0157364.

60. Ng, M.C.Y., Shriner, D., Chen, B.H., Li, J., Chen, W.M., Guo, X., Liu, J., Bielinski, S.J., Yanek, L.R., Nalls, M.A., et al. (2014). Meta-analysis of genome-wide association studies in African Americans provides insights into the genetic architecture of type 2 diabetes. PLoS Genet. *10*, e1004517.

61. Dai, N., Zhao, L., Wrighting, D., Krämer, D., Majithia, A., Wang, Y., Cracan, V., Borges-Rivera, D., Mootha, V.K., Nahrendorf, M., et al. (2015). IGF2BP2/IMP2-Deficient mice resist obesity through enhanced translation of Ucp1 mRNA and Other mRNAs encoding mitochondrial proteins. Cell Metabol. *21*, 609–621.

62. Regue, L., Minichiello, L., Avruch, J., and Dai, N. (2019). Liver-specific deletion of IGF2 mRNA binding protein-2/IMP2 reduces hepatic fatty acid oxidation and increases hepatic triglyceride accumulation. J. Biol. Chem. *294*, 11944–11951.

63. Cabili, M.N., Trapnell, C., Goff, L., Koziol, M., Tazon-Vega, B., Regev, A., and Rinn, J.L. (2011). Integrative annotation of human large intergenic noncoding RNAs reveals global properties and specific subclasses. Genes Dev. *25*, 1915–1927.

64. Deng, X., Su, R., Weng, H., Huang, H., Li, Z., and Chen, J. (2018). RNA N(6)-methyladenosine modification in cancers: current status and perspectives. Cell Res. *28*, 507–517.

65. Song, X., Chen, B., Liang, Y., Li, Y., Zhang, H., Han, D., Wang, Y., Ye, F., Wang, L., Zhao, W., and Yang, Q. (2022). CircEIF3H-IGF2BP2-HuR scaffold complex promotes TNBC progression via stabilizing HSPD1/RBM8A/G3BP1 mRNA. Cell Death Dis. *8*, 261.

66. Carpenter, S., and Fitzgerald, K.A. (2018). Cytokines and Long Noncoding RNAs. Cold Spring Harbor Perspect. Biol. *10*, a028589.

67. Mahmoud, L., Al-Enezi, F., Al-Saif, M., Warsy, A., Khabar, K.S.A., and Hitti, E.G. (2014). Sustained stabilization of Interleukin-8 mRNA in human macrophages. RNA Biol. *11*, 124–133.

68. Talukdar, S., Oh, D.Y., Bandyopadhyay, G., Li, D., Xu, J., McNelis, J., Lu, M., Li, P., Yan, Q., Zhu, Y., et al. (2012). Neutrophils mediate insulin resistance in mice fed a high-fat diet through secreted elastase. Nat. Med. *18*, 1407–1412.

69. Luo, Y., and Chen, C. (2021). The roles and regulation of the KLF5 transcription factor in cancers. Cancer Sci. *112*, 2097–2117.

70. Gao, Y., Wu, K., Chen, Y., Zhou, J., Du, C., Shi, Q., Xu, S., Jia, J., Tang, X., Li, F., et al. (2015). Beyond proliferation: KLF5 promotes angiogenesis of bladder cancer through directly regulating VEGFA transcription. Oncotarget *6*, 43791–43805.

71. Yang, C., Zheng, J., Xue, Y., Yu, H., Liu, X., Ma, J., Liu, L., Wang, P., Li, Z., Cai, H., and Liu, Y. (2017). The Effect of MCM3AP-AS1/miR-211/KLF5/AGGF1 Axis Regulating Glioblastoma Angiogenesis. Front. Mol. Neurosci. *10*, 437.

72. Waugh, D.J.J., and Wilson, C. (2008). The interleukin-8 pathway in cancer. Clin. Cancer Res. *14*, 6735–6741.

73. Corvera, S., Solivan-Rivera, J., and Yang Loureiro, Z. (2022). Angiogenesis in adipose tissue and obesity. Angiogenesis *25*, 439–453.

74. Liu, S.J., and Lim, D.A. (2018). Modulating the expression of long non-coding RNAs for functional studies. EMBO Rep. *19*, e46955.

75. Mattick, J.S., Amaral, P.P., Carninci, P., Carpenter, S., Chang, H.Y., Chen, L.L., Chen, R., Dean, C., Dinger, M.E., Fitzgerald, K.A., et al. (2023). Long non-coding RNAs: definitions, functions, challenges and recommendations. Nat. Rev. Mol. Cell Biol. *24*, 430–447.

76. Karagkouni, D., Paraskevopoulou, M.D., Tastsoglou, S., Skoufos, G., Karavangeli, A., Pierros, V., Zacharopoulou, E., and Hatzigeorgiou, A.G. (2020). DIANA-LncBase v3: indexing experimentally supported miRNA targets on non-coding transcripts. Nucleic Acids Res. *48*, D101–D110.

77. Ferguson, J.F., Patel, P.N., Shah, R.Y., Mulvey, C.K., Gadi, R., Nijjar, P.S., Usman, H.M., Mehta, N.N., Shah, R., Master, S.R., et al. (2013). Race and gender variation in response to evoked inflammation. J. Transl. Med. *11*, 63.

78. Alvarez-Dominguez, J.R., Bai, Z., Xu, D., Yuan, B., Lo, K.A., Yoon, M.J., Lim, Y.C., Knoll, M., Slavov, N., Chen, S., et al. (2015). De Novo Reconstruction of Adipose Tissue Transcriptomes Reveals Long Non-coding RNA Regulators of Brown Adipocyte Development. Cell Metabol. *21*, 764–776.

79. Ma, Y., Gao, J., Yin, J., Gu, L., Liu, X., Chen, S., Huang, Q., Lu, H., Yang, Y., Zhou, H., et al. (2016). Identification of a Novel Function of Adipocyte Plasma Membrane-Associated Protein (APMAP) in Gestational Diabetes Mellitus by Proteomic Analysis of Omental Adipose Tissue. J. Proteome Res. *15*, 628–637.

80. Chojnacki, S., Cowley, A., Lee, J., Foix, A., and Lopez, R. (2017). Programmatic access to bioinformatics tools from EMBL-EBI update: 2017. Nucleic Acids Res. *45*, W550–W553.

81. Hezroni, H., Koppstein, D., Schwartz, M.G., Avrutin, A., Bartel, D.P., and Ulitsky, I. (2015). Principles of long noncoding RNA evolution derived from direct comparison of transcriptomes in 17 species. Cell Rep. *11*, 1110–1122.

82. Schmidt, S.F., Jørgensen, M., Chen, Y., Nielsen, R., Sandelin, A., and Mandrup, S. (2011). Cross species comparison of C/EBPalpha and PPARgamma profiles in mouse and human adipocytes reveals interdependent retention of binding sites. BMC Genom. *12*, 152.

83. Bray, N.L., Pimentel, H., Melsted, P., and Pachter, L. (2016). Near-optimal probabilistic RNA-seq quantification. Nat. Biotechnol. *34*, 525–527.

84. Shevchenko, A., Tomas, H., Havlis, J., Olsen, J.V., and Mann, M. (2006). In-gel digestion for mass spectrometric characterization of proteins and proteomes. Nat. Protoc. *1*, 2856–2860.

85. Cox, J., and Mann, M. (2008). MaxQuant enables high peptide identification rates, individualized p.p.b.-range mass accuracies and proteome-wide protein quantification. Nat. Biotechnol. *26*, 1367–1372.

86. Cox, J., Neuhauser, N., Michalski, A., Scheltema, R.A., Olsen, J.V., and Mann, M. (2011). Andromeda: a peptide search engine integrated into the MaxQuant environment. J. Proteome Res. *10*, 1794–1805.

87. Heberle, H., Meirelles, G.V., da Silva, F.R., Telles, G.P., and Minghim, R. (2015). InteractiVenn: a web-based tool for the analysis of sets through Venn diagrams. BMC Bioinf. *16*, 169.

88. Szklarczyk, D., Kirsch, R., Koutrouli, M., Nastou, K., Mehryary, F., Hachilif, R., Gable, A.L., Fang, T., Doncheva, N.T., Pyysalo, S., et al. (2023). The STRING database in 2023: protein-protein association networks and functional enrichment analyses for any sequenced genome of interest. Nucleic Acids Res. *51*, D638–D646.

89. Galarraga, M., Campión, J., Muñoz-Barrutia, A., Boqué, N., Moreno, H., Martínez, J.A., Milagro, F., and Ortiz-de-Solórzano, C. (2012). Adiposoft: automated software for the analysis of white adipose tissue cellularity in histological sections. J. Lipid Res. *53*, 2791–2796.

90. Schindelin, J., Arganda-Carreras, I., Frise, E., Kaynig, V., Longair, M., Pietzsch, T., Preibisch, S., Rueden, C., Saalfeld, S., Schmid, B., et al. (2012). Fiji: an open-source platform for biological-image analysis. Nat. Methods *9*, 676–682.

91. Sherman, B.T., Hao, M., Qiu, J., Jiao, X., Baseler, M.W., Lane, H.C., Imamichi, T., and Chang, W. (2022). DAVID: a web server for functional enrichment analysis and functional annotation of gene lists (2021 update). Nucleic Acids Res. *50*, W216–W221.

STAR★METHODS

KEY RESOURCES TABLE

REAGENT or RESOURCE	SOURCE	IDENTIFIER
Antibodies		
IGF2BP2 antibody [RIP assay]	Millipore	03-251
HuR antibody	Millipore	07-468
GAPDH	Abcam	ab8245, RRID:AB_2107448
KLF5	Cell Signaling Technology	51586s, RRID:AB_2799395
PPARγ	Cell Signaling Technology	81B8
c-MYC	Cell Signaling Technology	9402, RRID:AB_2151827
DMRT2	Millipore	ABE1364
IGF2BP2 [WB +IF]	Thermo Scientific	712137, RRID:AB_2762371
Perilipin	Cell Signaling Technology	9349, RRID:AB_10829911
FABP4	Cell Signaling Technology	2120, RRID:AB_2102466
GLUT4	Abcam	ab33780, RRID:AB_2191441
CEBPα	Cell Signaling Technology	2295, RRID:AB_10692506
Adiponectin	Cell Signaling Technology	2789, RRID:AB_2221630
ATGL	Abcam	ab109251, RRID:AB_10864772
rabbit anti Mouse CD68	Abcam	ab283654, RRID:AB_2922954
rabbit anti-mouse F4/80	Cell Signaling Technology	70076S, RRID:AB_2799771
Opal 570	Akoya Biosciences	FP1488001KT
Opal 520	Akoya Biosciences	FP1487001KT
Bacterial and virus strains		
pLVX-shRNA2 vector	Clontech	N/A
psPAX2	Addgene	12260
pMD2.G	Addgene	12259
pcDNA3.1 with T7 promoter	Life-Technologies	V79020
Biological samples		
Human gluteal adipose tissue samples	GENE STUDY	NIH clinical trial NCT00953667
Abdominal subcutaneous and visceral omental adipose biopsies	Penn Human Adipose Resources	https://www.med.upenn.edu/idom/adipose.html
Chemicals, peptides, and recombinant proteins		
Human Epidermal Growth Factor carrier free	R&D Systems	236-EG
Human Fibroblast Growth Factor carrier free	R&D Systems	3718-FB
Recombinant Human Insulin	Sigma	91077C
Dexamethasone	Sigma	D2915
Isobutylmethylxanthine	Sigma	I5879
Rosiglitazone	Sigma	PHR2932
Panthothenate	Sigma	L8376
Collagenase Type 1	Roche	5172969103
Actinomycin D	Sigma	A9415
Critical commercial assays		
High-Capacity cDNA kit	Applied Biosystems	4368814
TaqMan Mouse Adgre1	ThermoFisher	Mm00802529_m1
TaqMan Mouse Cd68	ThermoFisher	Mm03047343
TaqMan Gene Expression Master Mix	Applied Biosystems	4370074

(Continued on next page)

Continued

REAGENT or RESOURCE	SOURCE	IDENTIFIER
TaqMan Mouse Hprt	ThermoFisher	Mm03024075_m1
TaqMan Mouse Rpl4	ThermoFisher	Mm05781370_g1
TaqMan Human IL-8	ThermoFisher	Hs0017413_m1
TaqMan Human CCL2	ThermoFisher	Hs00234140_m1
TaqMan Human PPARγ	ThermoFisher	Hs01115513_m1
TaqMan Human CEBPα	ThermoFisher	Hs00269972_s1
TaqMan Human HPRT	ThermoFisher	Hs99999909_g1
TaqMan Human RPL4	ThermoFisher	HS03044646_g1
Pierce^{TM} BCA kit	Sigma	23225
**Deposited data**		
RNA-seq data from the linc-ADAIN shRNA vs scramble shRNA ASC adipocytes	https://www.ncbi.nlm.nih.gov/geo/	GEO: GSE263852
Linc-ADAIN Pulldown assay in Human Adipocytes: The mass spectrometry proteomics data have been deposited to the ProteomeXchange Consortium via the PRIDE	https://www.proteomexchange.org/	PXD051388
**Experimental models: Cell lines**		
ASC52telo	ATCC	SCRC-4000
**Experimental models: Organisms/strains**		
NOD.Cg-Prkdcscid Il2rgtm1Wjl/SzJ (NSG) mice	Jackson Laboratory	#005557
**Oligonucleotides**		
Antisense LNA GapmeRs Negative control A	Qiagen	LG00000002
Antisense LNA gapmer used for IL-8	Qiagen	LG00839094
Antisense LNA gapmer used for KLF6	Qiagen	LG00839116
**Software and algorithms**		
Image J	Image J	https://imagej.net/ij/
Adiposoft plugin software Fiji	Fiji / Image J	https://imagej.net/plugins/adiposoft
Ingenuity Pathway (IPA)	Qiagen	N/A
Database for Annotation Visualization and Integrate Discovery (DAVID)	NIH	https://david.ncifcrf.gov/home.jsp
RBPmap	N/A	http://rbpmap.technion.ac.il/
**Other**		
Dulbecco’s modified Eagle’s medium/F-12	Gibco	11320033
Lipofectamine 3000	Invitrogen	L3000001
Direct-zol RNA miniprep kit	Zymo	R2052
SMARTer® RACE 5’/3’ Kit	clontech	Cat. Nos. 634858
TNT® Quick Coupled Transcription/ Translation System	Promega	L1170
Transcend® nonradioactive translation detection system	Promega	L5070
AmpliScribe T7 Flash Biotin RNA Transcription Kit	Lucigen	ASB71110
Protein A/G magnetic beads	Millipore	88802
Matrigel	Corning	356231
Nile Red	Invitrogen	N1142
Hoechst 33342	ThermoFisher	62249

(*Continued on next page*)

Continued

REAGENT or RESOURCE	SOURCE	IDENTIFIER
ACD Bio RNAscope Multiplex Fluorescent Reagent Kit v2	ACD Bio	#323100
SuperSignal™ West Pico PLUS chemiluminescent substrate	ThermoFisher	34580
MilliporeSigma™ Immobilon ECL Ultra Western HRP Substrate	Millipore	WBULS0500

RESOURCE AVAILABILITY

Lead contact

Further information and requests for resources and reagents should be directed to and will be fulfilled by the lead contact, Muredach P. Reilly (mpr2144@cumc.columbia.edu).

Materials availability

Requests for unique resources should be directed to the lead contact. A completed Materials Transfer Agreement may be required.

Data and code availability

- All data reported in this paper will be shared by the lead contact upon request.
- RNA-seq data and Mass Spec data have been deposited at https://www.ncbi.nlm.nih.gov/geo/ and https://www.proteomexchange.org/ and are publically available as of this publication. Accession numbers are listed in the key resources table.
- This paper does not report original code.
- Any additional information required to reanalyze the data reported in this paper is available from the lead contact upon request.

METHOD DETAILS

Experimental model and study participant details

Human participants

Human gluteal adipose tissue samples were collected in The Genetics of Evoked-responses to Niacin and Endotoxemia (GENE) study (N = 284, 33% African Americans, age 18-45), a National Institute of Health-sponsored protocol enrolled at University of Pennsylvania (Penn) (NIH clinical trial NCT00953667)[77]. Gluteal subcutaneous fat tissue samples were obtained at baseline, 4, 12 and 24 hours following intravenous LPS bolus (1ng/kg). As described in[16,17], RNA isolated from a subset of gluteal subcutaneous fat tissue samples underwent deep RNA-sequencing to identify adipose lincRNAs expressed and differentially modulated by LPS *in vivo*. Linc-DMRT2 (renamed to Linc-ADipose Anti-INflammatory) was identified as a top LPS-modulated lincRNA and its LPS-modulation was validated by qPCR in gluteal adipose tissue of an independent sample of healthy lean GENE subjects (Figure 1E). For clinical translation, qPCR was performed of gluteal subcutaneous adipose tissue from an independent sample of healthy lean GENE subjects (N=29, 48% female; 49% European ancestry; BMI ~21), as well as in abdominal subcutaneous and visceral omental adipose biopsies of obese subjects from the Penn Human Adipose Resources (https://www.med.upenn.edu/idom/adipose.html; N=39, BMI ~48)17 (Figures 1A–1C). All clinical studies were performed with the approval of the Penn Institutional Review Board and written informed consent was obtained from all research participants. Linc-ADAIN was chosen for functional study because 1) it showed high tissue specificity for adipose tissue and 2) it is modulated in obesity and during endotoxemia in human white adipose tissue. RNA-seq library preparation, sequencing and data processing, along with *de novo* assembly of human adipose transcriptomes for lincRNA discovery have all been previously described in detail in[16,17]. Linc-ADAIN genomic location is chr9 1044999-1048641 (isoform1), chr9 1044999-1048641 (isoform 2), chr9 1045640-1048641 (isoform 3).

Screening of human adipose proteome profiles as coding transcript filter

As previously described in[16]. Briefly to further exclude coding transcripts from annotated lincRNA catalogs and our *de novo* assembled putative lincRNAs, we screened human adipose proteome profiles for predicted peptides derived from these human lincRNA transcripts as previously described[78]. Published human adipose mass spectrometry (MS) shotgun profiling of deep coverage was used for our analyses[79]. Briefly, the *linc-ADAIN* transcripts (annotated and *de novo* assembled) were in silico translated into all possible peptides using Sixpack[80]. MS/MS spectra from human adipose MS profiling were searched against human UniProt database (20,205 reviewed canonical entries, version July 2017), MS common contaminants database and the custom in silico translated peptides from lincRNAs. MS raw files were analyzed with MaxQuant version 1.6.0.1 with a false discovery rate (FDR) of 1%. Variable modifications were indicated as methionine oxidation and protein N-terminal acetylation, while carbamidomethylation of cysteines was designated as a fixed modification. Trypsin, which cleaves after lysine and arginine residues, was selected as the digestion

enzyme and two missed cleavages were permitted. The main search peptide tolerance was set to 4.5 ppm and the first search tolerance was set to 20 ppm. iBAQ (intensity-based absolute quantification) was implemented for label-free quantification to accurately identify protein copy number. We identified over 28,075 high confidence peptides which mapped into 3,226 human proteins, including proteins whose mRNA abundances in adipose, e.g. KRT7 (FPKM 0.50) and SFN (FPKM 0.43), are low and similar to lincRNAs. In this context of robust profiling of protein coding genes, we found only 2 peptides which correspond to in silico translated ORFs from 2 lincRNAs (at FDR Q-value <0.05).

Synteny and conservation analysis of human adipose lincRNAs

As previously described in[16]. Briefly conserved lincRNAs are known to have limited sequence similarity with their orthologs but typically display genomic position conservation across species[81]. We examined the synteny of human lincRNA loci relative to mouse. For *linc-ADAIN*, we identified the two-neighboring protein-coding genes within ±500,000bp and searched for homologous genes in mouse using HomoloGene (http://www.ncbi.nlm.nih.gov/homologene). If two neighboring genes were homologous in mouse, the lincRNA locus was classified as syntenic. Next, we looked for evidence of mouse lincRNA transcription at syntenic regions in the mouse genome. We took a published mouse adipose lincRNAs transcriptome [78] and mapped those mouse lincRNAs to syntenic human regions. A human lincRNA was considered a conserved lincRNA if the mouse adipose lincRNAs was actually expressed in the syntenic region.

Transcription factor binding analysis and histone modification analysis

As previously described in[16]. Briefly we downloaded PPARγ and C/EBPα peaks identified in adipocytes derived from human Simpson-Golabi-Behmel syndrome (SGBS) pre-adipocytes (GSE27450)[82]. Peaks from two replicates for each transcription factor were merged. We then mapped the merged peaks to ±1,500bp of the TSS for three groups of human adipose lincRNAs based on their conservation with mouse: (1) lincRNAs with mouse adipose lincRNA transcripts expressed in the syntenic loci, (2) lincRNA with syntenic loci but no mouse lincRNA expression in the region and (3) lincRNAs non-syntenic in mouse. Mapping was done at the transcript level. We then counted the number of lincRNAs with PPARγ and C/EBPα binding as well as the total number of binding events for adipose lincRNAs. For Spearman correlation analyses between lincRNA adipose abundance/fractional expression and the distance of nearby transcription factor binding sites to lincRNA TSS, we identified the midpoints of transcription factor ChIPseq peaks for lincRNAs with transcription factor binding within ±1500bp and calculated the distances between lincRNA TSS and midpoint of transcription factor binding peak. For lincRNA genes with multiple TF binding, the smallest transcription factor binding site-TSS distances were used.

Tissue expression and single-cell expression of linc-ADAIN *in adipose tissue*

Gene expression in TPM and the associated sample attributes were downloaded from GTEx v8. Mean expression was calculated for each tissue and transformed by log(TPM + 1). TPMs were visualized on heatmap in a subset of 28 tissues from GTEx. Single cell UMAP data for *linc01230* was taken from the Single Cell Portal – Broad Institute (https://singlecell.broadinstitute.org) using single cell sequencing data from human white adipose tissue by Emont et al., 2022 [27].

Mouse studies

All animal experiments were approved by the Institutional Animal Care and Use Committee of Columbia University (protocol AC-AABR1551). NOD.Cg-Prkdcscid Il2rgtm1Wjl/SzJ (NSG) mice were obtained from The Jackson Laboratory (#005557) and bred in our animal facility. T method was adapted from previously published[23,24]. Female mice were used at 12 to 14 weeks of age. Under anesthesia, mice were injected subcutaneously into the flank regions with 50:50 (vol/vol) Matrigel (Corning 356231) and ASC hTERT adipocytes with lentiviral expression of shRNA targeting linc-ADAIN or scramble control as described above. ASC hTERT are differentiation for 14 days *in vitro* before injection, as described above. Mice were on standard chow diet and after 16 weeks, the mice were euthanized, and tissues and human adipose implants were removed for further study. Fourteen weeks after implantation, mice underwent a glucose tolerance test (GTT) or insulin tolerance test (ITT). (N= 6 for scramble shR, N=4 for Linc-ADAIN shR). Mice were fasted overnight then received an i.p injection of 1.5g/kg glucose and blood samples collected at times indicated to measure blood glucose using a/ glucometer. For ITT mice were fasted for 5 hours then received an i.p injection of 0.5U/kg of insulin and blood samples collected at times indicated to measure blood glucose.

Human adipocyte culture

ASC52telo, hTERT immortalized adipose derived mesenchymal stem cells were purchased from ATCC (SCRC-4000). Primary human adipose stromal cells (ASC) were extracted from freshly isolated subcutaneous adipose tissue. Briefly, subcutaneous adipose samples were minced and digested with collagenase (1 mg/ml) (Roche) for 1 hour. The digestion mixture was filtered and rinsed through sterile cell strainer. The stromal fraction cells were collected by centrifugation. Both hTERT ASCs and primary ACS were expanded in Dulbecco's modified Eagle's medium/F-12 media supplemented with 10% fetal bovine serum and human EGF (10 μg/L) and FGF (1 μg/L). To induce adipocyte differentiation, confluent ASCs or hTERT ASCs were incubated in differentiation media containing insulin (1.7μM), dexamethasone (1μM), isobutylmethylxanthine (500 μM), Rosiglitazone (2 μM), panthothenate (17μM) and biotin (33μM). After 7 days, cells were incubated with maintenance media containing insulin (1.7μM), dexamethasone (1μM), panthothenate (17μM) and biotin (33μM) for an additional 7 days (a total of 14 days culture), unless otherwise stated.

ShRNA KD of linc-ADAIN *in ASC hTERTs*

shRNA sequences were obtained using an open-access hairpin design tool from the RNAi Consortium, Broad Institute (http://www.broadinstitute.org/rnai/public/seq/search). These shRNA sequences were cloned into pLVX-shRNA2 vector containing ZsGreen

(Clonetech). The pLVX-shRNA2 vector was transfected into LentiX-293T cells (Clonetech) with packaging vectors psPAX2 (Addgene plasmid 12260) and pMD2.G (Addgene plasmid 12259) using Lipofectamine 3000 (Life technologies). Forty-eight hours after transfection, virus supernatants were collected and centrifuged at 1000g for 5 min to remove cell debris. For lentiviral infection, confluent ASCs hTERTs were treated with lentiviral supernatant and 5 μg/mL polybrene. Cells were expanded and flow activated cell sorted (FACS) for ZsGreen-positive cells. ShRNA KD efficiency was assessed by measuring relative gene expression by qPCR.

shRNA sequences:

Scramble shRNA (Target sequence)

CGTACGCGGAATACTTCGATTCAAGAGATCGAAGTATTCCGCGTACGTTTTTTTT

Linc-ADAIN shRNA sequence (Target sequence)

GGCGGATTGATTGAGTGTAATTTCAAGAGAATTACACTCAATCAATCCGCCTTTTTT

Antisense oligonucleotide (ASO) KD of linc-ADAIN, KLF5 and IL-8

Confluent primary ASCs were transfected with Antisense LNA GapmeRs (Qiagen) targeting *linc-ADAIN* (50nM) or negative control A (50nM) (LG00000002) using Lipofectamine RNAiMax Regent (Thermo Fisher Scientific) at day 0,3,6,9,12 of differentiation. Multiple Gapmers were tested for KD efficiency by measuring *linc-ADAIN* by qPCR. One was chosen for functional studies. Antisense LNA gapmer used for KLF5: LG00839094 and for IL-8: LG00839116

Antisense LNA gapmer target sequence used for *linc-ADAIN*:

TGGGAAACTAGATTGA

RNA-sequencing and analysis of differentially expressed genes

RNA samples were extracted using Trizol and Direct-zol RNA miniprep kit (Zymo Research R2052) from ASC-adipocytes. Following DNase treatment, RNA samples were assessed using Agilent Bioanalyzer (Agilent, Santa Clara, CA) and all samples had an RNA Integrity Number (RIN) larger than 9. With a minimum of 200ng input RNA. Libraries were prepared by Columbia Genome center using Illumina Stranded mRNA prep kit (Illumina, San Diego, CA) for subsequent sequencing on Illumina's NextSeq 500/550 obtaining ~80 million reads per sample. The estimated counts and transcripts per million (TPM) values for each gene were obtained from a pseudoalignment to a kallisto index derived from human transcriptomes (GRCh38) using kallisto version 0.44.0[83]. Differential gene expression analysis was performed using DESeq2 version 1.32.0. 17,174 genes with at least 10 reads total in the estimated counts were used to compare the expressions in RNA-seq data from scramble vs *linc-ADAIN* shRNA KD in ASC adipocytes. DESeq2 independent filtering was also applied to exclude count outliers and low mean count across samples. 1,403 genes with at least 2-fold difference between two conditions (scramble vs *linc-ADAIN* shRNA KD) and FDR adjusted P-value of less than 0.05 were considered differentially expressed genes. Heatmap was generated using Morpheus (Broad Institute).

Cellular fractionation

A 10cm plate of mature adipocytes was trypsinized and collected in 1ml ice-cold PBS. 2 x150ul aliquots for total lysate were centrifuged at 2000g for 3mins 4°C and resuspended in 1x Dautry Buffer + enzymes (2x Dautry buffer, Tris pH 7.4 10mM, NaCl 10mM, $MgCl_2$ 3mM, EDTA 1.5mM + 0.5% NP40, RNAse inhibitor, protease inhibitor). 700ul of adipocytes resuspended in PBS was centrifuged and then resuspended in 200ul 1x Dautry + enzymes and incubated on ice for 5min. Sample was centrifuged. The supernatant was collected and centrifuged and this supernatant was the cytoplasmic fraction. The pellet was resuspended in 1x Dautry Buffer + enzymes (nuclear fraction). Total lysate, cytoplasmic fraction and nuclear fraction was used for downstream RNA extraction or western blot.

3' RACE (rapid amplification of cDNA ends)

3' RACE was performed in purified RNAs of mature ASC-adipocytes using SMARTer RACE 5'/3′ Kit (clontech) as per manufacturer's instructions.

In vitro transcription/translation assay

Linc-ADAIN pcDNA3.1 expression vector T7 promoter Life-Technologies and TNT® Quick Coupled Transcription/Translation System (Promega) were used to *in vitro* transcribe and translate the full-length lincRNA. The T7 luciferase expression control plasmid supplied with the kit was used as the positive control. Protein products from transcription/translation reactions were labeled with biotinylated lysine. 2 ul of the reaction products were added with 15 ul SDS sample buffer, heat denatured and resolved in NuPage SDS–polyacrylamide gel (Life Technologies). Biotin-labeled proteins were subsequently detected using Transcend® nonradioactive translation detection system (Promega).

RNA pulldown assay and mass spectrometry

Linc-ADAIN or Anti-sense *linc-ADAIN* were cloned into pcDNA3.1 vector downstream of a T7 promoter (Life Technologies). The expression plasmid was linearized and transcribed *in vitro* using AmpliScribe T7 Flash Biotin RNA Transcription Kit (Lucigen ASB71110) following the manufacturer's instructions. Biotinylated RNAs were subsequently purified with RNeasy spin columns (Qiagen). For the pulldown assay, RNA (5000ng in 20ul) was refolded for proper secondary structure formation by heating for 2 min at 90°C and add RNA structure buffer (20mM trish ph7, 0.2M KCL, 20mmM MgCL2), incubate at RT for 30mins. Adipocyte lysates were collected from ~1x107 mature ASC-derived adipocytes in 2 ml RNA immunoprecipitation (RIP) buffer (150 mM KCl, 25 mM

Tris at pH 7.4, 5 mM EDTA, 0.5mM DTT, 0.5% NP40, 1X protease inhibitor cocktail, 100 U/ml RNAseOUT). Shear cells on ice with dounce homogenizer. The lysate was cleared from cell debris by centrifugation at 15,000g for 15 min at 4°C. Protein concentration in the lysate was determined using the BCA protein assay kit. RNA pulldown was carried out as previously described in[16]. Proteins were separated by 4-12% gradient Bis-Tris gels followed by coomassie blue staining (Thermo Fisher).

For the mass spectrometry (MS) analysis, gel band was excised and in-gel digestion was performed as previously described[84]. Peptides were dissolved in 3% acetonitrile/0.1% formic acid.

LC-MS/MS analysis

Thermo Scientific™ UltiMate™ 3000 RSLCnano system and Thermo Scientific EASY Spray™ source with Thermo Scientific™ Acclaim™ PepMap™100 2 cm x 75 μm trap column and Thermo Scientific™ EASY-Spray™ PepMap™ RSLC C18 50 cm x 75 μm ID column were used to separate desalted peptides with a 5-30% acetonitrile gradient in 0.1% formic acid over 50 min at a flow rate of 250 nL/min. The column temperature was maintained at a constant 50 °C during all experiments. Thermo Scientific™ Orbitrap Fusion™ Tribrid™ mass spectrometer was used for peptide MS/MS analysis. Survey scans of peptide precursors were performed from 400 to 1500 m/z at 120K FWHM resolution (at 200 m/z) with a 2 x 10^5 ion count target and a maximum injection time of 50 ms. The instrument was set to run in top speed mode with 3 s cycles for the survey and the MS/MS scans. After a survey scan, tandem MS was performed on the most abundant precursors exhibiting a charge state from 2 to 6 of greater than 5 x 10^3 intensity by isolating them in the quadrupole at 1.6 Th. CID fragmentation was applied with 35% collision energy, and resulting fragments were detected using the rapid scan rate in the ion trap. The AGC target for MS/MS was set to 1 x 10^4 and the maximum injection time was limited to 35 ms. The dynamic exclusion was set to 45 s with a 10-ppm mass tolerance around the precursor and its isotopes. Monoisotopic precursor selection was enabled.

Data analysis

Raw mass spectrometric data were analyzed using the MaxQuant environment[85] v.1.6.1.0 and employed Andromeda for database search[86] at default settings with a few modifications. The default was used for the first search tolerance and main search tolerance: 20 ppm and 6 ppm, respectively. MaxQuant was set up to search with the reference human proteome database downloaded from Uniprot. MaxQuant performed the search trypsin digestion with up to 2 missed cleavages. Peptide, Site, and protein FDR were all set to 1% with a minimum of 1 peptide needed for identification, and label-free quantitation (LFQ) was performed with a minimum ratio count of 1. The following modifications were used as variable modifications for identifications and included for protein quantification: Oxidation of methionine (M), Acetylation of the protein N-terminus, and Deamination for asparagine or glutamine (NQ). Results obtained from MaxQuant were further uploaded on Scaffold. Accession number.

RNA-binding proteins (RBP) map and STRING analysis

RNA sequences for *linc-ADAIN*, IL-8, IL-6, MCP-1 were uploaded to RBP map to predict RBP sites[29]. Predicted RBPs for *linc-ADAIN*, IL-8, IL-6, MCP-1 as well as detected proteins from *linc-ADAIN* RNA pulldown assay were overlapped in a Venn diagram using IneractiVenn[87]. The overlapping 7 proteins, HuR, IGF2BP2, G3BP2, FXR2, MATR3, FMR1, SFPQ were uploaded to STRING[88] to search for their possible interactions and biological processes.

RNA immunoprecipitation (RIP)

1 x 10cm plate of ASC- hTERT adipocytes were resuspended in 1 ml of RIP buffer (150mM KCl, 25 mM Tris, pH 7.4, 5 mM EDTA, 0.5% IGEPAL CA-630 (Sigma), 0.5mM DTT, 100 U/ml RNAseOUT, and 1X protease inhibitor cocktail), sheared with a Dounce homogenizer then sonicated at 20 amplitude for 10seconds followed by centrifugation at 15,000g for 15 min at 4°C. Lysates were pre-cleared with washed Protein A/G magnetic beads (Millipore) at 4°C for 30 minutes. 30 μl Protein A/G magnetic beads (Millipore) were incubated with 1.25ug IGF2BP2 antibody (Millipore 03-251), 10ug HuR antibody (Millpore 07-468) or IgG control in 200 μl RIPA buffer for 30 min at room temperature followed by incubation with pre-cleared lysate for 4 hours at 4°C. Bead samples were washed five times in RIP buffer. The beads were resuspended in 500μl of Trizol and RNA extracted. RNA pellets were precipitated in isopropanol overnight at 80°C. The pellet was resuspended in 15 μL of RNase-free water for cDNA synthesis and qPCR analysis.

RNA isolation and quantitative real-time PCR analysis

Total RNA was extracted using Trizol and Direct-zol RNA miniprep kit (Zymo Research R2052). At least 100ng of RNA was reverse transcribed using High-Capacity cDNA kit (Applied Biosystems). Expression of lincRNAs and protein-coding genes was assessed by quantitative real-time PCR using an Applied Biosystems PowerUp SYBR Green system. Custom DNA oligos targeting *linc-ADAIN* were designed using Genescript PCR primer design tool (genescript.com) and obtained from Integrated DNA technologies. Pre-designed PrimeTime qPCR primer assays were used and obtained from Integrated DNA technologies for all other genes. *GAPDH, HPRT* and *18S* were used as housekeeping genes. Adipose Explant macrophage gene expression was assessed using mouse specific TaqMan primers (ThermoFisher, Mouse Adgre1 Mm00802529_m1, Cd68 Mm03047343) and TaqMan Gene Expression Master Mix (Applied Biosystems). Housekeeping genes were Mouse Hprt and Rpl4 (ThermoFisher Mouse Hprt Mm03024075_m1, Mouse Rpl4 Mm05781370_g1). All other genes were human specific TaqMan primers (ThermoFisher, IL-8 Hs0017413_m1, CCL2 Hs00234140_m1, PPARγ Hs01115513_m1, CEBPα Hs00269972_s1, housekeeping HPRT Hs99999909_g1, RPL4 HS03044646_g1). Fold changes were calculated relative to housekeeping genes using the comparative Ct method.

Linc-ADAIN Forward Primer: ACAGGCGCATTCCACCACGC
Linc-ADAIN Reverse Primer: TCCTTGGCCCCACTCCGCAA

RNA stability assays

Day 14 ASC hTERT adipocytes are treated with 5ug/ml Actinomycin D (to pause transcription) in or DMSO control in F12 + 0.2% BSA media, for 30mins, 1,2,4 hrs. Cells were washed with PBS, RNA extracted with Trizol and qPCR performed as per protocol above. Calculated decay rate as by subtracting control CT value from sample CT (ΔCT) and then calculated ΔΔCT ($2^{-\Delta CT}$).

Immunoblot analysis

Total cell lysates were prepared in RIPA buffer supplemented with protease inhibitors (Roche). Protein concentration was quantified by Pierce BCA kit. About 10-15 μg protein lysate were reduced, separated by SDS-PAGE electrophoresis, and transferred to nitrocellulose membranes. Blots were blocked with 10% nonfat milk in Tris-buffered saline containing 0.1% Tween 20 and incubated with primary antibodies overnight at 4 °C. Primary antibodies against GAPDH (Abcam ab8245), KLF5 (CST 51586s), PPARγ (CST 81B8), c-MYC (CST 9402), DMRT2 (Millipore, ABE1364), IGF2BP2 (Thermo Scientific, 712137), HuR (Millipore 07-468), Perilipin (CST 9349), FABP4 (CST 2120), GLUT4 (Abcam ab33780), CEBPα (CST 2295), Adiponectin (CST 2789), ATGL (Abcam ab109251) were used according to manufacturer's instructions to probe membranes. Blots were then incubated with HRP-conjugated secondary antibodies at room temperature for 1 hour and visualized with SuperSignal™ West Pico PLUS chemiluminescent substrate (Thermo Fisher 34580) or MilliporeSigma™ Immobilon ECL Ultra Western HRP Substrate (WBULS0500).

Human adipocyte implantation into mice

All animal experiments were approved by the Institutional Animal Care and Use Committee of Columbia University (protocol AC-AABR1551). NOD.Cg-Prkdcscid Il2rgtm1Wjl/SzJ (NSG) mice were obtained from The Jackson Laboratory (#005557) and bred in our animal facility. T method was adapted from previously published[23,24]. Female mice were used at 12 to 14 weeks of age. Under anesthesia, mice were injected subcutaneously into the flank regions with 50:50 (vol/vol) Matrigel (Corning 356231) and ASC hTERT adipocytes with lentiviral expression of shRNA targeting *linc-ADAIN* or scramble control as described above. ASC hTERT are differentiation for 14 days *in vitro* before injection, as described above. Mice were on standard chow diet and after 16 weeks, the mice were euthanized, and tissues and human adipose implants were removed for further study. Fourteen weeks after implantation, mice underwent a glucose tolerance test (GTT) or insulin tolerance test (ITT). (N= 6 for scramble shR, N=4 for Linc-ADAIN shR). Mice were fasted overnight then received an i.p injection of 1.5 g/kg glucose and blood samples collected at times indicated to measure blood glucose using a glucometer. For ITT mice were fasted for 5 hours then received an i.p injection of 0.5U/kg of insulin and blood samples collected at times indicated to measure blood glucose.

Immunohistochemistry staining (IHC)

Adipose explants were placed in 4% paraformaldehyde overnight. Using the Histology Service of the Molecular Pathology Shared Resource at the Columbia University Irving Medical Center, explants were paraffin-embedded and 5 μm blank slides were sectioned. Slides were stained with hematoxylin and eosin. IHC staining was performed on 5 μm-thick FFPE sections with rabbit anti Mouse CD68 (Abcam ab283654) and rabbit anti-mouse F4/80 (Cell Signaling 70076S) antibodies to detect macrophages. Antigen retrieval was performed in 10 mmol/L citrate buffers (pH 6.0) with a pressure cooker. Quenching endogenous peroxidase activity was done with 3%H_2O_2 in PBS for 10 minutes at room temperature. Slides were incubated with primary antibody CD68 1:200 or F4/80 1:400 dilutions 2hours at room temperature, followed by anti-rabbit polymer (DAKO #K4003). Detection used HRP catalyzed chromogen "diaminobenzidine". Counterstaining was with hematoxylin followed by dehydration, clearing in xylene, and mounting with Permount. Imaging was with a Leica SCN400. Adipocytes sizes were calculated using Image J and Adiposoft plugin software [89]. The CD68 and F4/80 staining area was quantified using Fiji/Image J[90].

Nile red staining

Day 14 or Day 21 differentiation ASC hTERT adipocytes were fixed in 2% PFA then stained with 0.5uM Nile Red (Invitrogen N1142) and Hoechst 33342 (Thermo Fisher) for 30mins. Images were acquired with epifluorescence Nikon Ti-S inverted microscope at 20x resolution. Nile Red Area/ Nuclei quantification used triangle or otsu thresholding algorithms in Fiji/ Image J.[90]

RNA scope immunofluorescence (IF) assay

ASC hTERT human adipocytes are plated on 24 well dishes containing glass coverslips and differentiated according to the protocol above. After 14 days differentiation, cells are dehydrated with 50, 70 and 100% ethanol incubations (5mins), then rehydrated with 100, 70 and 50% ethanol incubations (5mins). Cells were permeabilized for 5mins using pre-treatment #3 from ACD Bio RNA-scope Multiplex Fluorescent Reagent Kit v2 (ACD Bio #323100) then processed with manufacturers RNA scope protocol. ACD Bio designed custom RNA scope probes for *linc-ADAIN*. Using ACD Bio protocol for RNA Scope and combined IF, antibodies for IGF2BP2 (Thermo Fisher #712137) and HuR (Millipore 07-468) with secondary antibodies Opal 570 and Opal 520 (Akoya Biosciences, FP1488001KT, FP1487001KT) were used to probe adipocytes. Slides were imaged using a scanning confocal: Nikon A1 with GaAsP spectral detector and Nikon Ti Eclipse inverted microscope for Z-stack images, at 20x and 40x objective magnifications.

Ingenuity pathway (IPA) and database for annotation visualization and Integrate discovery (DAVID) gene ontology analysis

The list of differentially expressed genes (DEGs) from RNA-sequencing was uploaded to Qiagen's IPA system for core analysis and overlaid with the global molecular network in the IPA knowledge base (https://digitalinsights.qiagen.com/citation-guidelines/). Molecular and Cellular Functions were explored to interpret the effects of *linc-ADAIN* KD compared to scramble control on adipocyte functions. DEGs were also uploaded to DAVID database to search for top enriched gene ontology (GO) biological pathways[91].

QUANTIFICATION AND STATISTICAL ANALYSIS

All statistics are outlined in each figure legend. In brief, Mann Whitney U non-parametric test was applied to analysis with two groups, One-Way ANOVA was applied to three or more groups, and a Two-Way ANOVA was applied when there were two or more quantitative variables with two or more categorical variables. Pearson correlations were used to assess the correlation of human *linc-ADAIN* expression to other variables in the GENE study. GraphPad Prism 9 (GraphPad Software Inc, San Diego, CA) was used for statistical analysis. Data in graphs are presented as mean± standard error of the mean.

Cell Reports, Volume *43*

Supplemental information

linc-ADAIN, a human adipose lincRNA, regulates adipogenesis by modulating KLF5 and IL-8 mRNA stability

Marcella E. O'Reilly, Sebastian Ho, Johana Coronel, Lucie Zhu, Wen Liu, Chenyi Xue, Eunyoung Kim, Esther Cynn, Caio V. Matias, Rajesh Kumar Soni, Chen Wang, Iuliana Ionita-Laza, Robert C. Bauer, Leila Ross, Yiying Zhang, Silvia Corvera, Susan K. Fried, and Muredach P. Reilly

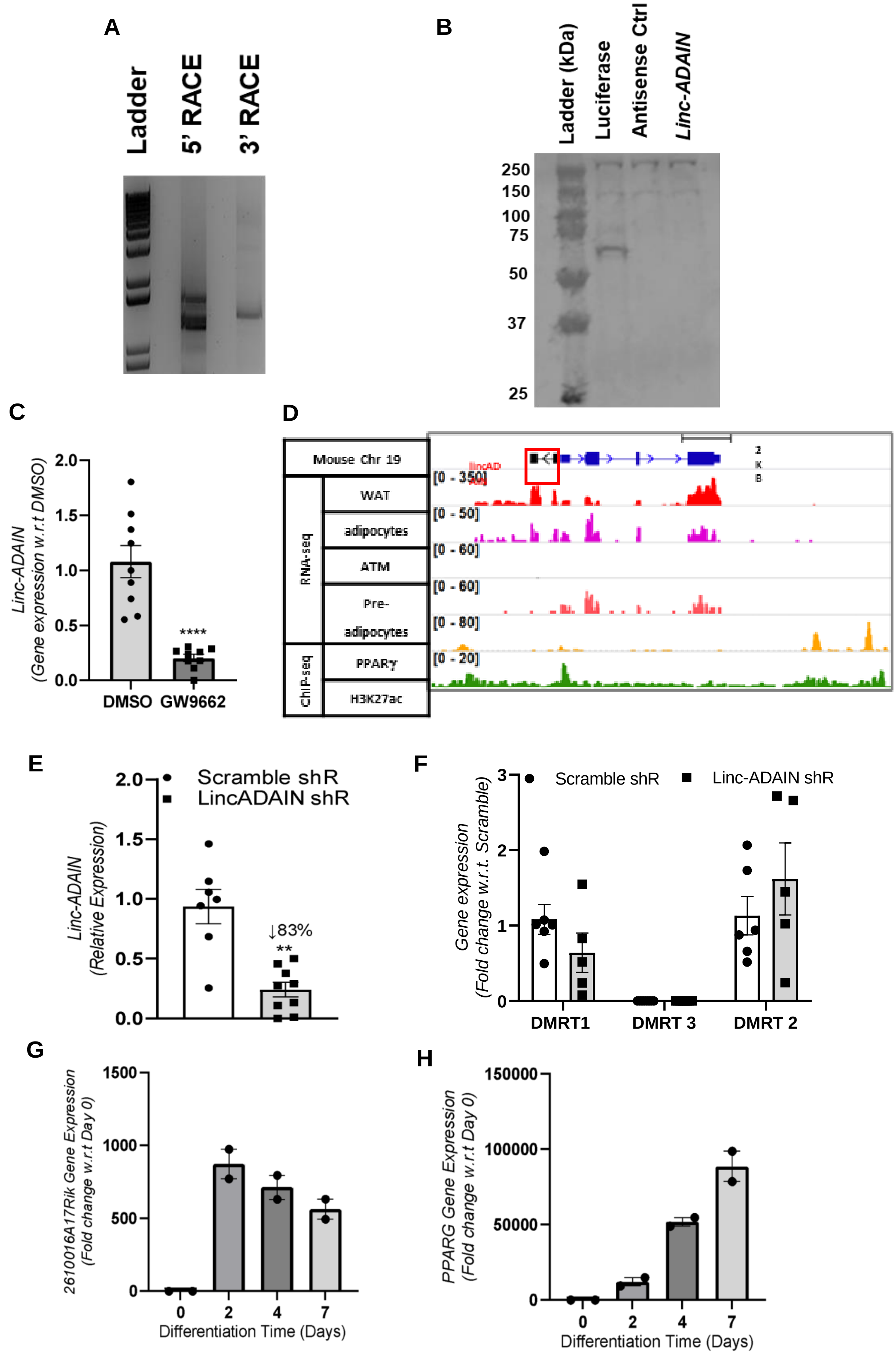
A
Ladder
5' RACE
3' RACE
B
Ladder (kDa)
Luciferase
Antisense Ctrl
Linc-ADAIN
250
150
100
75
50
37
25
C
Linc-ADAIN
(Gene expression w.r.t DMSO)
2.0
1.5
1.0
0.5
0.0
****
DMSO
GW9662
D
Mouse Chr 19
RNA-seq
WAT
adipocytes
ATM
Pre-adipocytes
ChIP-seq
PPARγ
H3K27ac
lincAD
[0 - 350]
[0 - 50]
[0 - 60]
[0 - 60]
[0 - 80]
[0 - 20]
2 KB
E
Scramble shR
LincADAIN shR
Linc-ADAIN
(Relative Expression)
2.0
1.5
1.0
0.5
0.0
↓83%
**
F
Scramble shR
Linc-ADAIN shR
Gene expression
(Fold change w.r.t. Scramble)
3
2
1
0
DMRT1
DMRT 3
DMRT 2
G
2610016A17Rik Gene Expression
(Fold change w.r.t Day 0)
1500
1000
500
0
0
2
4
7
Differentiation Time (Days)
H
PPARG Gene Expression
(Fold change w.r.t Day 0)
150000
100000
50000
0
0
2
4
7
Differentiation Time (Days)

Supplement Figure 1. *Linc-ADAIN* does not code for protein and the characteristics of *linc-ADAIN's* syntenic locus in mouse. The 3' RACE products of *linc-ADAIN* in human ASC-adipocytes (A), An *in vitro* transcription/translation assay using a pcDNA3.1 expressing *linc-ADAIN* or antisense *linc-ADAIN* and a luciferase vector as the positive control (B). Suppression of linc-ADAIN by PPARγ antagonist (GW9662 10μM (C). Regulatory features in the mouse syntenic region of *linc-ADAIN*, RNA-seq coverage (mouse adipose and adipocytes), transcription factor binding and active histone modification markers (D). qPCR of linc-ADAIN (E) and nearby protein coding genes, DMRT1, DMRT3, DMRT2 (F) upon linc-ADAIN KD via shRNA vs Scramble control in mature ASC hTERT adipocytes (N=3) **p<0.01 w.r.t Scramble by Two Mann Whitney U Test. 3T3-L1 adipocytes were differentiated in vitro and RNA extracted at the times indicated. Gene expression for mouse 2610016A17Rik. (G) and PPARG (H) was measured by qPCR at the times indicated. (N=2) Data presented as ± SEM.

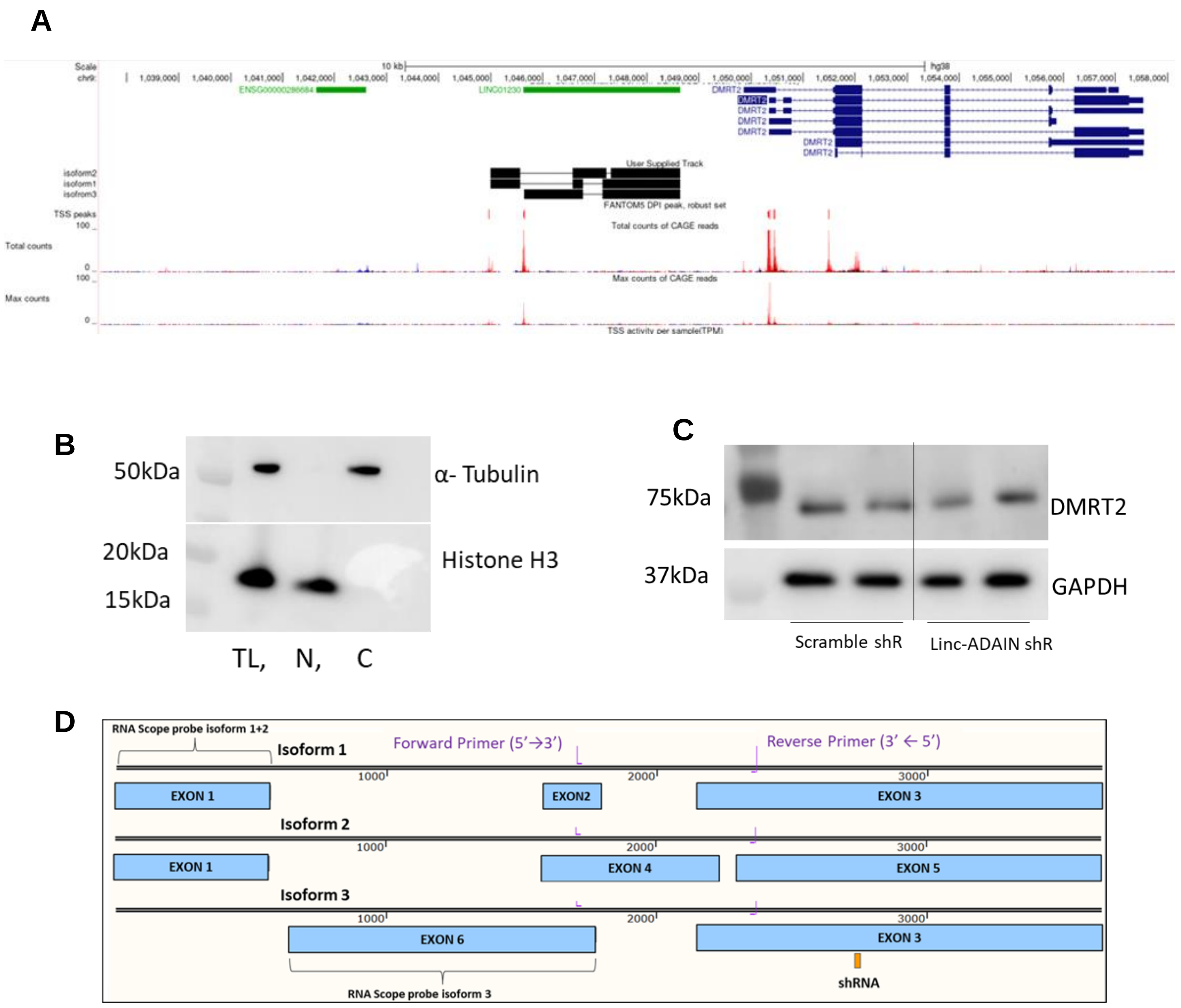

Supplement Figure 2. *Linc-ADAIN’s* genome locus and map of isoforms. Genome Browser view of *linc-ADAIN* locus showing 5’TSS and total CAGE reads (A). WB showing fractionation of adipocytes into nuclear [N] and cytoplasm [B] fractions, probing for α-tubulin (cytoplasm) and histone H3 (nuclear) (B). WB of DMRT2 expression upon *linc-ADAIN* KD via shRNA in adipocytes (C). Map of *linc-ADAIN Isoforms*, exons, qPCR primer location, RNA scope probe location and shRNA target sequence (D).

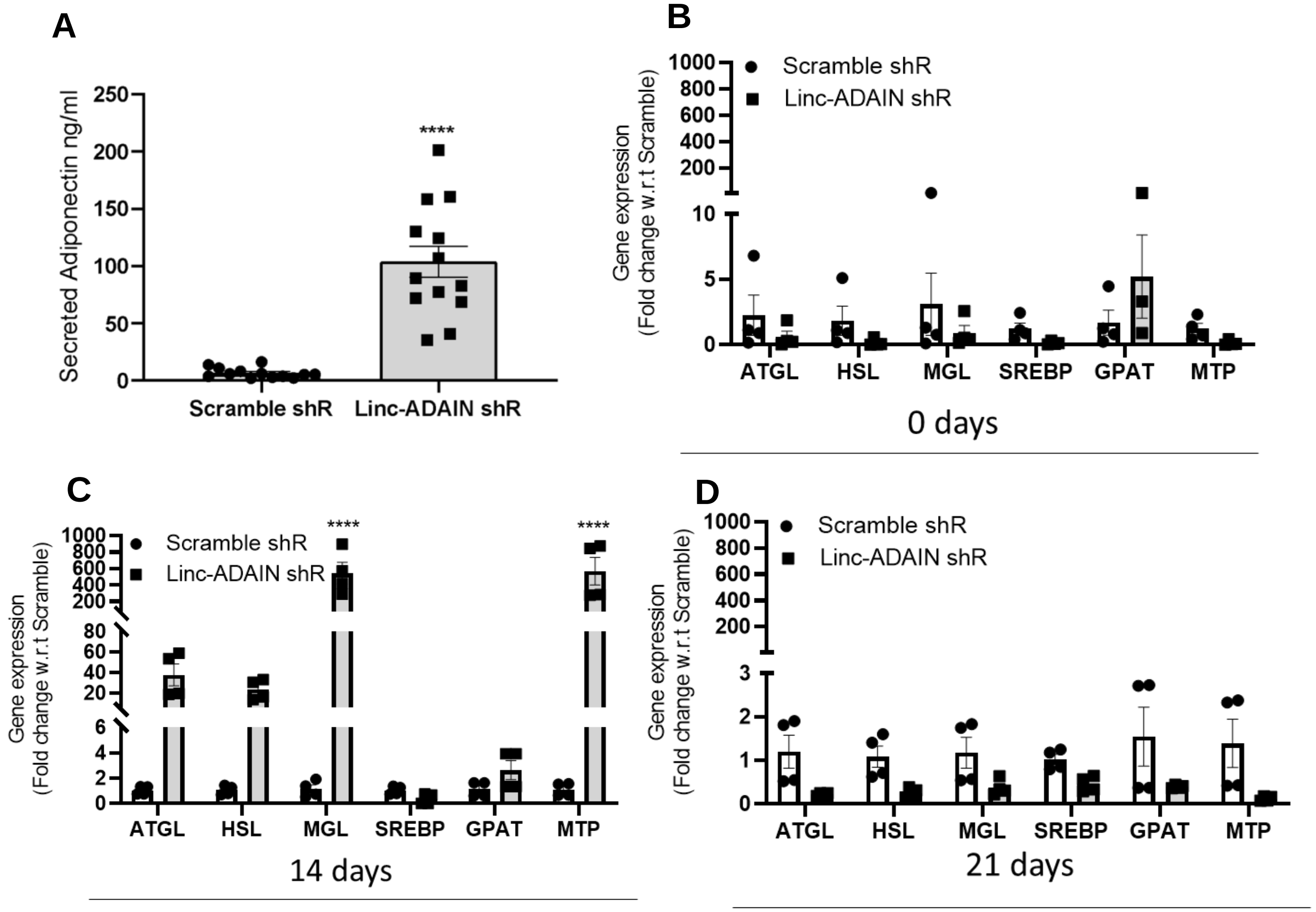

<u>Human cytokine array</u>

E

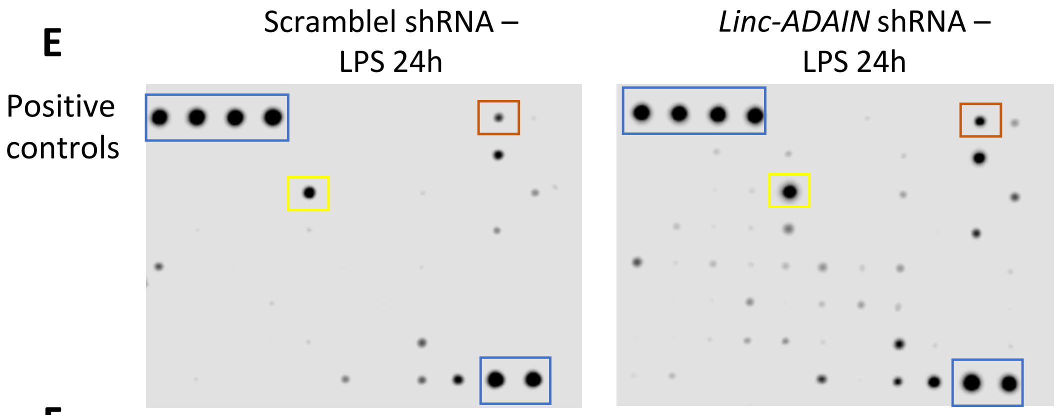

F

	A	B	C	D	E	F	G	H	I	J	K
1	POS	POS	POS	POS	NEG	NEG	ENA-78 (CXCL5)	G-CSF	GM-CSF	GRO a/b/g	GRO alpha (CXCL1)
2	I-309 (CCL1)	IL-1 alpha (IL-1 F1)	IL-1 beta (IL-1 F2)	IL-2	IL-3	IL-4	IL-5	IL-6	IL-7	IL-8 (CXCL8)	IL-10
3	IL-12 p40/p70	IL-13	IL-15	IFN-gamma	MCP-1 (CCL2)	MCP-2 (CCL8)	MCP-3 (CCL7)	M-CSF	MDC (CCL22)	MIG (CXCL9)	MIP-1 beta (CCL4)
4	MIP-1 delta (CCL15)	RANTES (CCL5)	SCF	SDF-1 alpha	TARC (CCL17)	TGF beta 1	TNF alpha	TNF beta (TNFSF1B)	EGF	IGF-1	Angiogenin
5	OSM	TPO	VEGF-A	PDGF-BB	Leptin	BDNF	BLC (CXCL13)	Ck beta 8-1 (CCL23)	Eotaxin-1 (CCL11)	Eotaxin-2 (CCL24)	Eotaxin-3 (CCL26)
6	FGF-4	FGF-6	FGF-7 (KGF)	FGF-9	FLT-3 Ligand	Fractalkine (CX3CL1)	GCP-2 (CXCL6)	GDNF	HGF	IGFBP-1	IGFBP-2
7	IGFBP-3	IGFBP-4	IL-16	IP-10 (CXCL10)	LIF	LIGHT (TNFSF14)	MCP-4 (CCL13)	MIF	MIP-3 alpha (CCL20)	NAP-2 (CXCL7)	NT-3
8	NT-4	OPN (SPP1)	OPG (TNFRSF11B)	PARC	PLGF	TGF beta 2	TGF beta 3	TIMP-1	TIMP-2	POS	POS

Supplement Figure 3. Effect of *linc-ADAIN* knock-down on adipogenic genes and secretion of cytokines from adipocytes. ASC hTERT adipocytes with scramble or *linc-ADAIN* shRNA expression with Adiponectin ELISA (A), ****$p<0.0001$ w.r.t to scramble shRNA by Mann Whitney U test. ASC hTERTs expressing Scramble or *linc-ADAIN* shRNA were grown in culture and RNA samples extracted at Day 0 (B), before differentiation or cells were differentiated to adipocytes and RNA extracted at Day 14 (C) and Day 21 (D) and genes ATGL, HSL, MGL, SREBP, GPAT and MTP measured by qPCR. ***$p<0.0001$ w.r.t Scramble shR (N=4). ASC hTERTs expressing Scramble or linc-ADAIN shRNA were differentiated to adipocytes and at day 14, 100ng of LPS was added to each group for 24hrs. Media was collected and applied to a Human Cytokine Antibody Array C5 (RayBio C Series) to detect changes in adipocyte cytokine secretion. Yellow box highlighs MCP-1, the orange box highlights IL-8 (E) Map of Cytokine Array (F). Data presented as ± SEM.

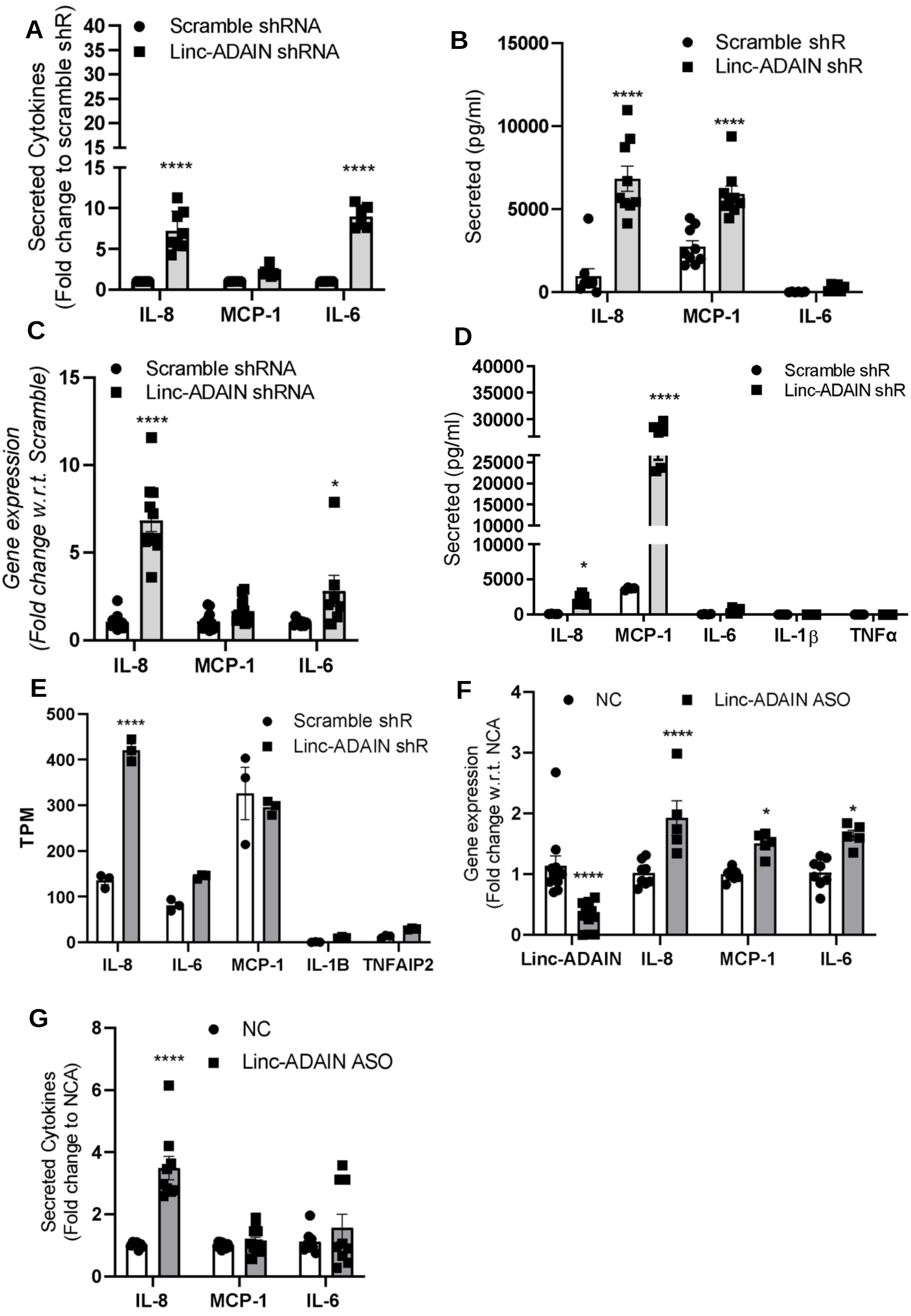
A
Scramble shRNA
Linc-ADAIN shRNA
Secreted Cytokines
(Fold change to scramble shR)
IL-8
MCP-1
IL-6
B
Scramble shR
Linc-ADAIN shR
Secreted (pg/ml)
IL-8
MCP-1
IL-6
C
Scramble shRNA
Linc-ADAIN shRNA
Gene expression
(Fold change w.r.t. Scramble)
IL-8
MCP-1
IL-6
D
Scramble shR
Linc-ADAIN shR
Secreted (pg/ml)
IL-8
MCP-1
IL-6
IL-1β
TNFα
E
Scramble shR
Linc-ADAIN shR
TPM
IL-8
IL-6
MCP-1
IL-1B
TNFAIP2
F
NC
Linc-ADAIN ASO
Gene expression
(Fold change w.r.t. NCA)
Linc-ADAIN
IL-8
MCP-1
IL-6
G
NC
Linc-ADAIN ASO
Secreted Cytokines
(Fold change to NCA)
IL-8
MCP-1
IL-6

Supplement Figure 4. Effect of *linc-ADAIN* knock-down in adipocytes on cytokine expression by ELISA, qPCR and RNA-sequencing. *Linc-ADAIN* knockdown increases cytokine secretion. Fold change of MCP-1, IL-8 and IL-6 via secretion ELISA (A) and expression of mRNA levels by qPCR (C) (N=3 in triplicate. Protein secretion of MCP-1, IL-8 and IL-6 via ELISA as pg/ml (D). **p<0.01, ****p<0.0001 w.r.t. scramble shR by Two-Way ANOVA (B) Luminex adipocyte panel of adipocyte media as pg/ml (N=3 in duplicate) (D). Transcripts per million (TPM) of cytokine mRNAs via RNA-seq upon *linc-ADAIN* KD (N=1 in triplicate) (E). Expression of *linc-ADAIN*, *IL-8*, *MCP-1* and *IL-6* by qPCR (F) and corresponding secretion via ELISA (G) upon knockdown of *linc-ADAIN* using an Antisense Oligonucleotide (ASO) throughout differentiation in primary human ASC adipocytes (N=3 in duplicate) ****p<0.0001, *p<0.05 w.r.t. NC (negative control) by Two-Way ANOVA. Data presented as ± SEM.

A

Molecular and Cellular Functions		
Name	p-value range	# Molecules
Cellular Movement	1.71E-07 - 7.96E-53	608
Cell Death and Survival	1.25E-07 - 2.34E-30	698
Cellular Development	1.02E-07 - 2.02E-22	641
Cellular Growth and Proliferation	5.82E-08 - 2.02E-22	618
Cell-To-Cell Signaling and Interaction	1.51E-07 - 2.44E-19	346

B

Term	Count	P-Value	Fold Enrichment	Bonferroni
GO:0007155~cell adhesion	145	2.63E-21	2.24	1.74E-17
GO:0001525~angiogenesis	72	8.31E-13	2.44	5.50E-09
GO:0006954~inflammatory response	94	2.40E-10	1.95	1.59E-06
GO:0030335~positive regulation of cell migration	66	3.06E-09	2.14	2.02E-05
GO:0007165~signal transduction	216	5.73E-09	1.46	3.79E-05

Supplement Figure 5: Pathway analyses of RNA-seq data of scramble vs linc-ADAIN shRNA ASC adipocytes. Top molecular and cellular functions analyzed by Ingenuity Pathway Analysis (A) Top enriched gene ontology (GO) biological process pathways analyzed by DAVID (B).

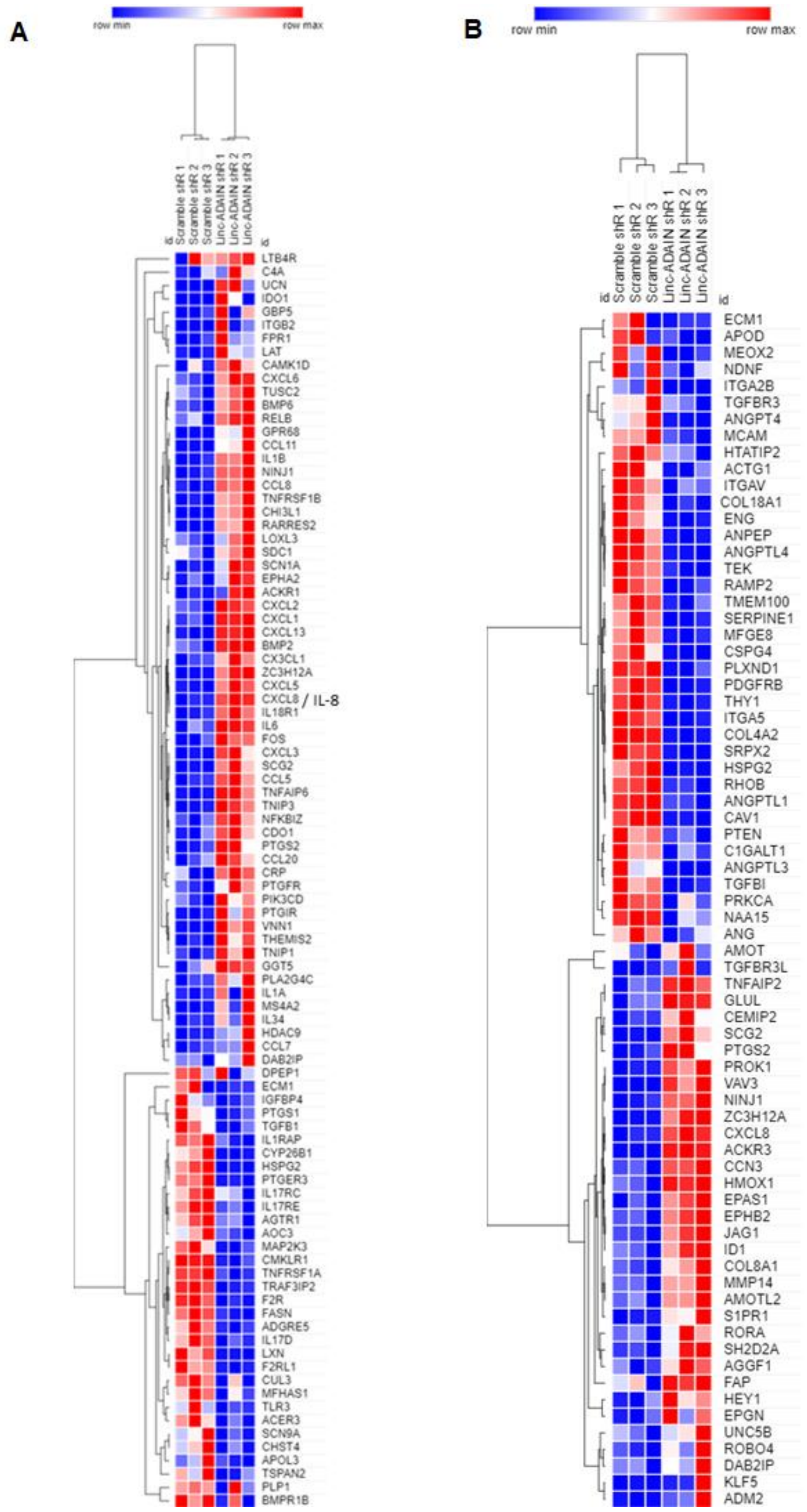

Supplement Figure 6: Differential gene expression analyses of RNA-seq data of scramble vs *linc-ADAIN* shRNA ASC hTERT adipocytes. Heatmap of genes in GO:0006954-inflammatory response (A) and GO:0001525- angiogenesis (B) pathways.

A

		Predicted by RBP map											
		IL8			MCP1			IL6			KLF5		
**Protein**	**Protein Location**	**Z-score**	**P-value**	**# of binding motifs**	**Z-score**	**P-value**	**# of binding motifs**	**Z-score**	**P-value**	**# of binding motifs**	**Z-score**	**P-value**	**# of binding motifs**
**HuR**	Nucleus and Cytoplasm	3.923	0.000	63	3.593	0.000	33	3.418	0.000	40	3.89	0.000	86
**IGF2BP2**	Cytoplasm	3.914	0.000	87	3.086	0.001	21	3.557	0.000	37	4.438	0.000	97
**G3BP2**	Cytoplasm	3.147	0.000	8	2.733	0.003	4	3.04	0.001	9	2.48	0.006	18
**FXR2**	Cytoplasm	3.2	0.000	6	2.657	0.003	1	2.614	0.004	7	2.9	0.001	13
**MATR3**	Nucleus	3	0.001	48	2.554	0.005	16	2.581	0.004	22	3.5	0.000	22
**FMR1**	Cytoplasm	3.403	0.000	6	3	0.001	2	3.194	0.000	8	3.625	0.000	9
**SFPQ**	Nucleus	3.518	0.000	63	3.494	0.0002	22	3.611	0.0001	44	3.723	0.000	86

B

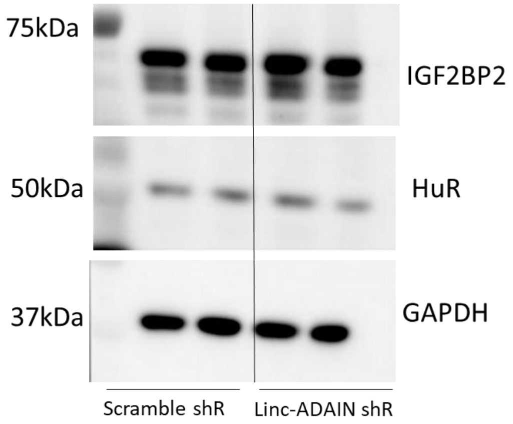

Supplement Figure 7. Predicted RNA binding proteins to *linc-ADAIN, IL-8, MCP-1, IL-6* and *KLF5* RNA. Predicted binding of HuR, IGF2BP2, G3BP2, FXR2, MATR3, FMR1 and SFPQ with *IL-8*, *MCP-1*, *IL-6* and *KLF5* mRNA, using RBPmap (A). Western blot showing total protein expression of IGF2BP2 (top band) and HuR in scramble and *linc-ADAIN* shRNA ASC hTERT adipocytes at day 14 (B).

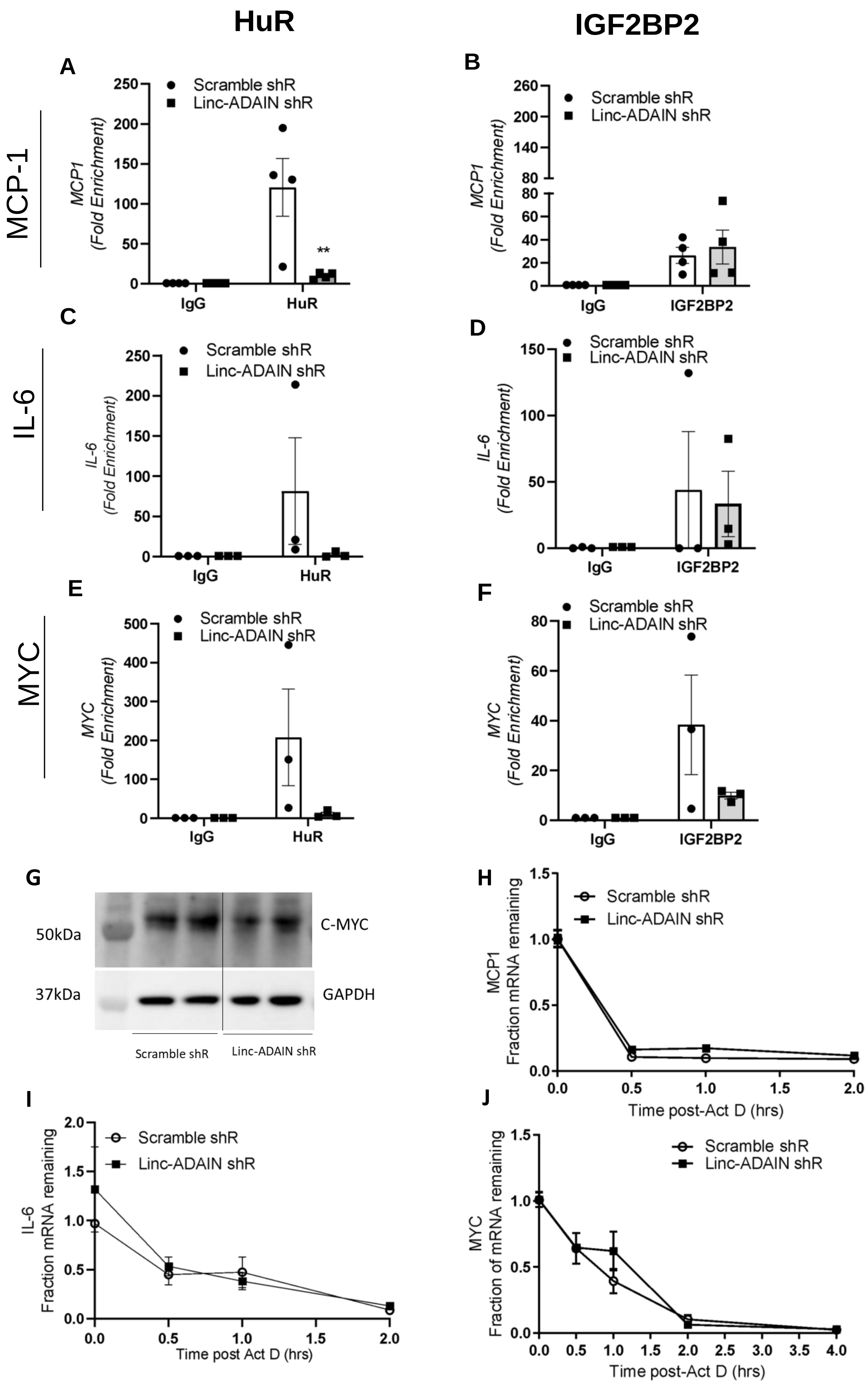
HuR
IGF2BP2
A
B
C
D
E
F
G
H
I
J
MCP-1
IL-6
MYC
Scramble shR
Linc-ADAIN shR
MCP1 (Fold Enrichment)
IL-6 (Fold Enrichment)
MYC (Fold Enrichment)
IgG
HuR
IGF2BP2
**
50kDa
37kDa
C-MYC
GAPDH
Scramble shR
Linc-ADAIN shR
MCP1 Fraction mRNA remaining
IL-6 Fraction mRNA remaining
MYC Fraction of mRNA remaining
Time post-Act D (hrs)
Time post Act D (hrs)

Supplement Figure 8. Knockdown of *linc-ADAIN* has no effect on MCP-1, IL-6 or MYC mRNA stability in adipocytes. RIP assays measure interaction of *MCP-1, IL6* and *MYC* RNA with HuR (A,C,E) and IGF2PBP2 (B,D,F) (N=3). Western blot of c-MYC and GAPDH (G) in total cell lysate of scramble and *linc-ADAIN* shRNA expressing ASC hTERT adipocytes. Scramble and *linc-ADAIN* shRNA expressing ASC adipocytes are treated with Actinomycin-D to halt transcription and gene expression measured at 0.5, 1,2,4 hrs post treatment of *MCP-1* (H), *IL-6* (I) and MYC (J) (N=3). *$p<0.05$, ****$p<0.0001$ w.r.t. Scramble shRNA by Two-Way ANOVA. Data presented as ± SEM.

A

IGF2BP2 RIP -seq HEK293T (Huang et al., 2018)				RNA-seq (O'Reilly)	
	input_fpkm	IP_fpkm	log2_fold_change	log2FoldChange	padj
KLF5	2.17207	266.459	6.9387	2.342851569	2.45E-12
CKB	6.79268	731.398	6.75053	-1.338784679	0.002436
SHC3	0.980199	86.3956	6.46174	2.765955332	2.82E-63
MDK	1.30221	103.159	6.30776	1.623701433	0.010746
MARCKSL1	0.735022	50.7861	6.1105	-1.546004986	2.46E-21
PEMT	0.906179	47.6256	5.7158	-1.279895348	1.35E-16
KIF7	0.554491	24.9756	5.49321	-1.473954504	9.03E-40
SESN2	0.647789	25.3862	5.29238	1.84900647	1.64E-30
SLC7A5	1.20549	37.9904	4.97795	1.485927587	1.4E-07
HOXB9	0.788867	24.6233	4.9641	2.773095577	0.021855
AMOT	3.02988	88.2888	4.8649	2.080302909	0.013361
UCHL1	1.70578	44.2523	4.69725	2.308569022	3.08E-09
PLK1	2.68766	65.6484	4.61034	1.36567595	0.004373
SAMD11	2.07017	48.3633	4.54609	-1.445887132	1.28E-17
COL4A2	1.84746	42.4879	4.52343	-1.451249202	1.29E-94
PGD	1.9669	43.4925	4.46677	-1.41934195	1.32E-39
PTPRF	6.90417	152.596	4.46611	-1.826218547	2.2E-22
COL4A1	0.523423	10.2679	4.29402	-1.540018336	4.66E-96

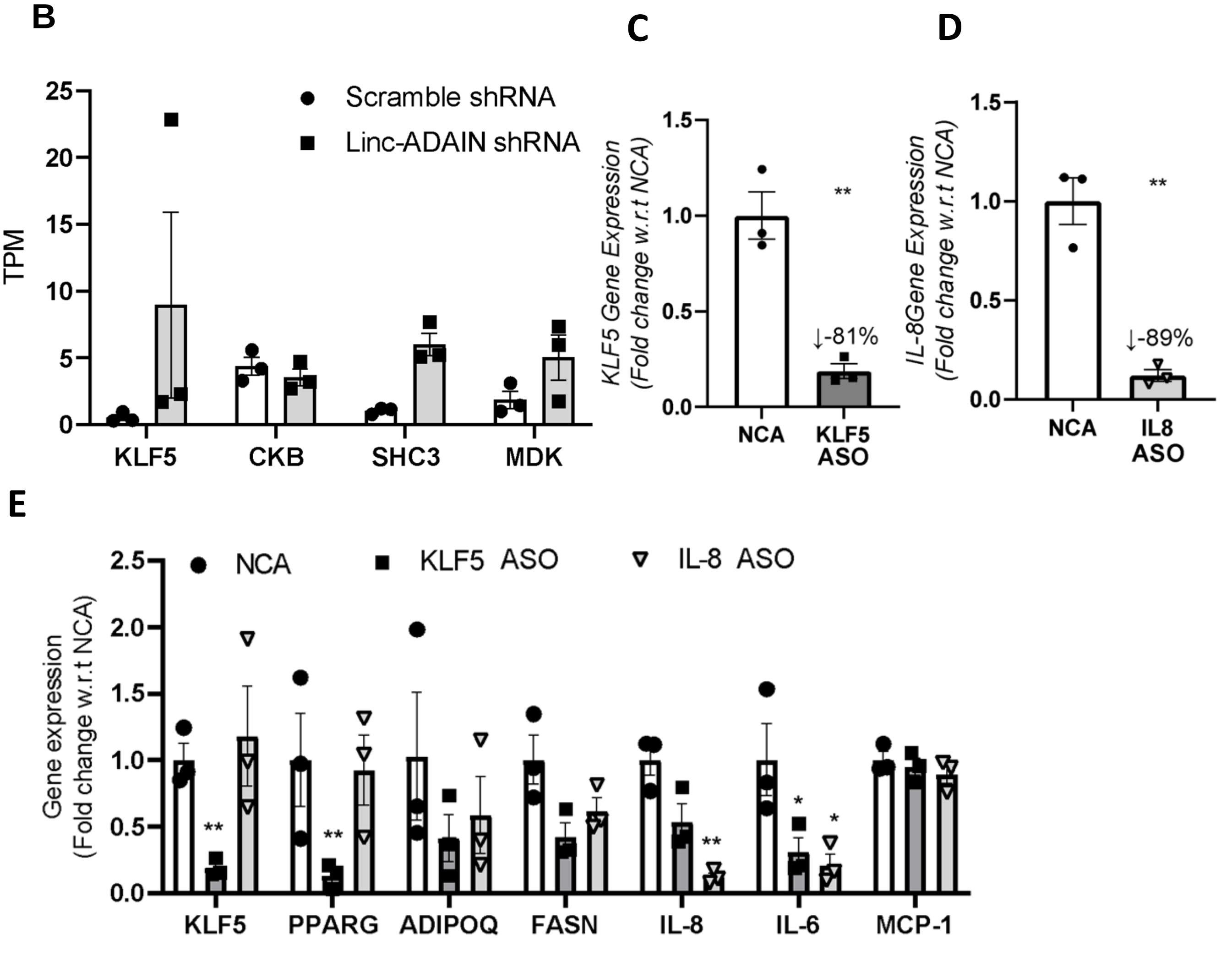

Supplement Figure 9. KLF5 interacts IGF2BP2 and its expressed increased upon *linc-ADAIN* knock-down in adipocytes. We cross referenced IGF2BP2 –RIP seq data set from HEK293T cells differential gene expression data from *linc-ADAIN* vs Scramble shRNA bulk RNA-seq data. Table shows the mRNAs with biggest fold change in IGF2BP2 vs IgG in HEK cells and biggest differential expression in bulk RNA-seq data from *linc-ADAIN* KD experiment. KLF5 was the top target (A). Transcripts per million (TPM) of top 4 mRNAs, *KLF5, CKB, SHC3, MDK* expression in RNA-seq data from scramble vs *linc-ADAIN* shRNA KD in ASC adipocytes (B). Adipocyte Stromal Cell hTERTs (ASC-hTERTs) were differentiated to day 6 then treated with ASO Gapmers designed against IL-8 and KLF5 and a Negative Control A (NCA), at day 6,9 and 12 of adipocyte differentiation. RNA was extracted at Day 14 and genes measured by qPCR KLF5, (C) IL-8, (D) KLF5, PPARG, FASN, IL-8, IL-6 (E) (N=3). **p<0.01, *p>0.05 w.r.t. NCA. Data presented as ± SEM.

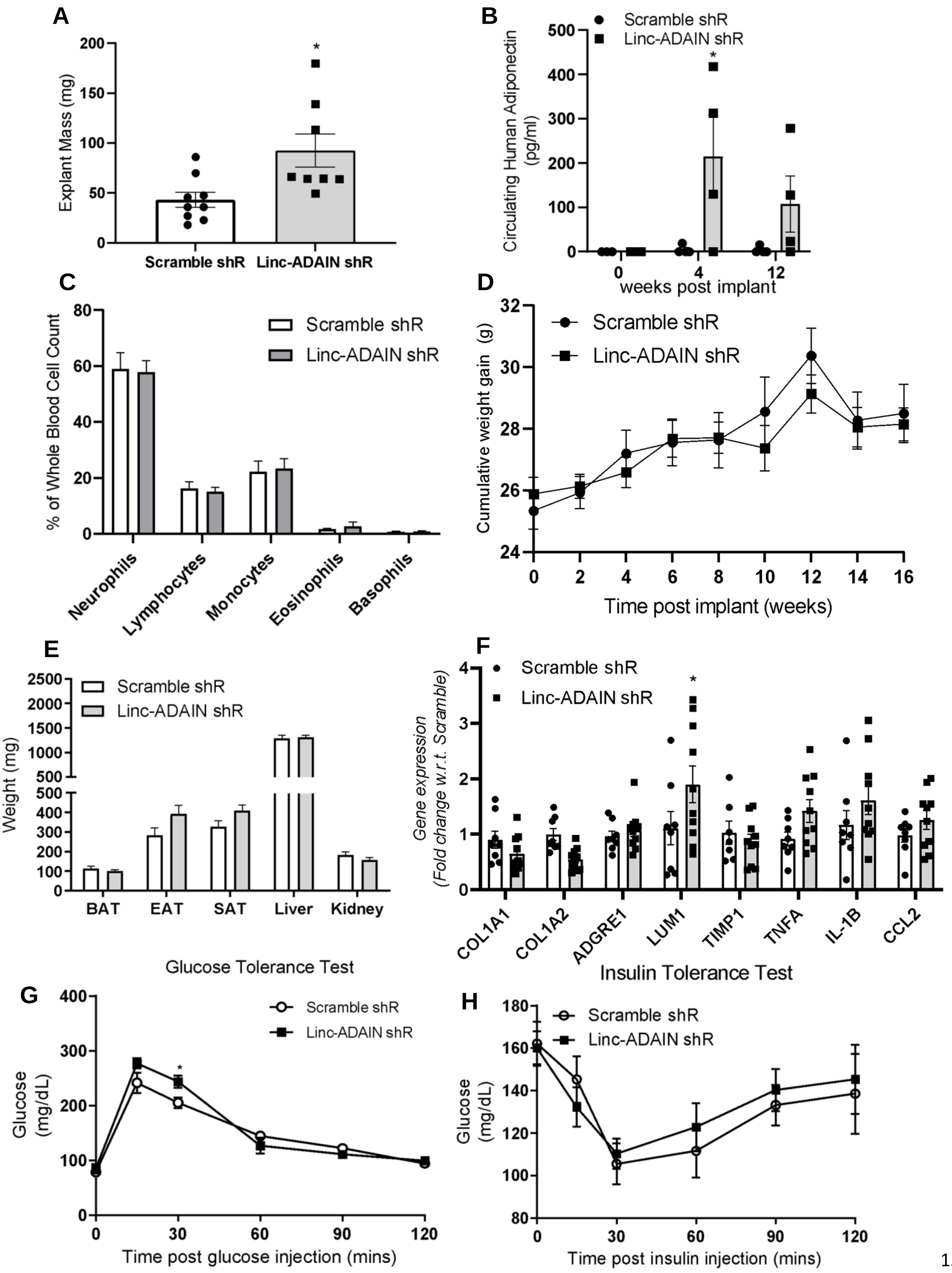
A
Explant Mass (mg)
Scramble shR
Linc-ADAIN shR
B
Scramble shR
Linc-ADAIN shR
Circulating Human Adiponectin (pg/ml)
weeks post implant
C
% of Whole Blood Cell Count
Neurophils
Lymphocytes
Monocytes
Eosinophils
Basophils
D
Cumulative weight gain (g)
Time post implant (weeks)
E
Weight (mg)
BAT
EAT
SAT
Liver
Kidney
F
Gene expression (Fold change w.r.t. Scramble)
COL1A1
COL1A2
ADGRE1
LUM1
TIMP1
TNFA
IL-1B
CCL2
Glucose Tolerance Test
G
Glucose (mg/dL)
Time post glucose injection (mins)
Insulin Tolerance Test
H
Glucose (mg/dL)
Time post insulin injection (mins)

Supplement Figure 10. *Linc-ADAIN* shRNA adipocyte implants into NSG mice had little effect on metabolic markers or fatty liver markers compared to Scramble shRNA. Human adipose explant weight after 16 weeks *$p<0.05$ w.r.t. scramble by Mann Whitney U test (A). Circulating human Adiponectin in plasma from mice at 4- and 12-weeks post implant *$p<0.05$ w.r.t. scramble by Two-Way ANOVA (B) (Pooled 4 mice from 4 batch's). Complete Blood cell count (C), Cumulative weight gain (D), Organ weights (E). 16 weeks after mice were implanted, mice were euthanized, and livers collected. RNA was extracted from livers and gene expression of multiple markers (COL1A1, COL1A2, ADGRE1, LUM1, TIMP1, TNFA, IL-1B and CCL2) of fatty liver was measured by qPCR, (N=9 per group), $p<0.05$ w.r.t. scramble shR. (F) 14 weeks after mice were implanted, Mice fasted overnight then received an i.p injection of 1.5g/kg glucose and blood samples collected at times indicated to measure blood glucose. (G). Mice fasted for 5 hours then received an i.p injection of 0.5U/kg of insulin and blood samples collected at times indicated to measure blood glucose. (H) N= 6 for scramble shR, N=4 for Linc-ADAIN shR. Data presented as ± SEM.
